# Recycling of Post-Consumer Waste Polystyrene Using Commercial Plastic Additives

**DOI:** 10.1021/acscentsci.4c01317

**Published:** 2024-11-25

**Authors:** Sewon Oh, Hanning Jiang, Liat H. Kugelmass, Erin E. Stache

**Affiliations:** † Department of Chemistry and Chemical Biology, Cornell University, Ithaca, New York 14850, United States; ‡ Department of Chemistry, Princeton University, Princeton, New Jersey 08544, United States

## Abstract

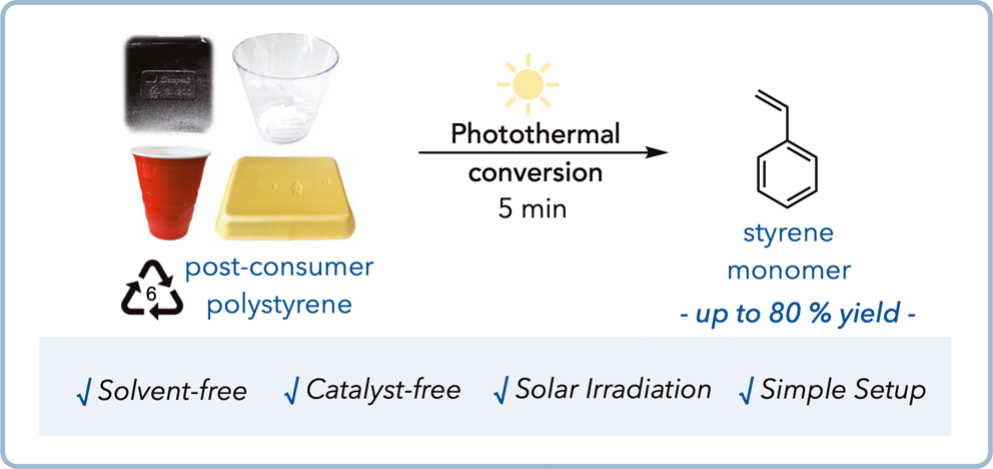

Photothermal conversion can promote plastic depolymerization (chemical recycling to a monomer) through light-to-heat conversion. The highly localized temperature gradient near the photothermal agent surface allows selective heating with spatial control not observed with bulk pyrolysis. However, identifying and incorporating practical photothermal agents into plastics for end-of-life depolymerization have not been realized. Interestingly, plastics containing carbon black as a pigment present an ideal opportunity for photothermal conversion recycling. Herein, we use visible light to depolymerize polystyrene plastics into styrene monomers by using the dye in commercial black plastics. A model system is evaluated by synthesizing polystyrene–carbon black composites and depolymerizing under white LED light irradiation, producing styrene monomer in up to 60% yield. Excitingly, unmodified postconsumer black polystyrene samples are successfully depolymerized to a styrene monomer without adding catalysts or solvents. Using focused solar irradiation, yields up to 80% are observed in just 5 min. Furthermore, combining multiple types of polystyrene plastics with a small percentage of black polystyrene plastic enables full depolymerization of the mixture. This simple method leverages existing plastic additives to actualize a closed-loop economy of all-colored plastics.

## Abstract 2

Simple light irradiation transforms postconsumer polystyrene products to a styrene monomer, using heat from the additives already found in many commercial products.

## Introduction

**Figure 1:**
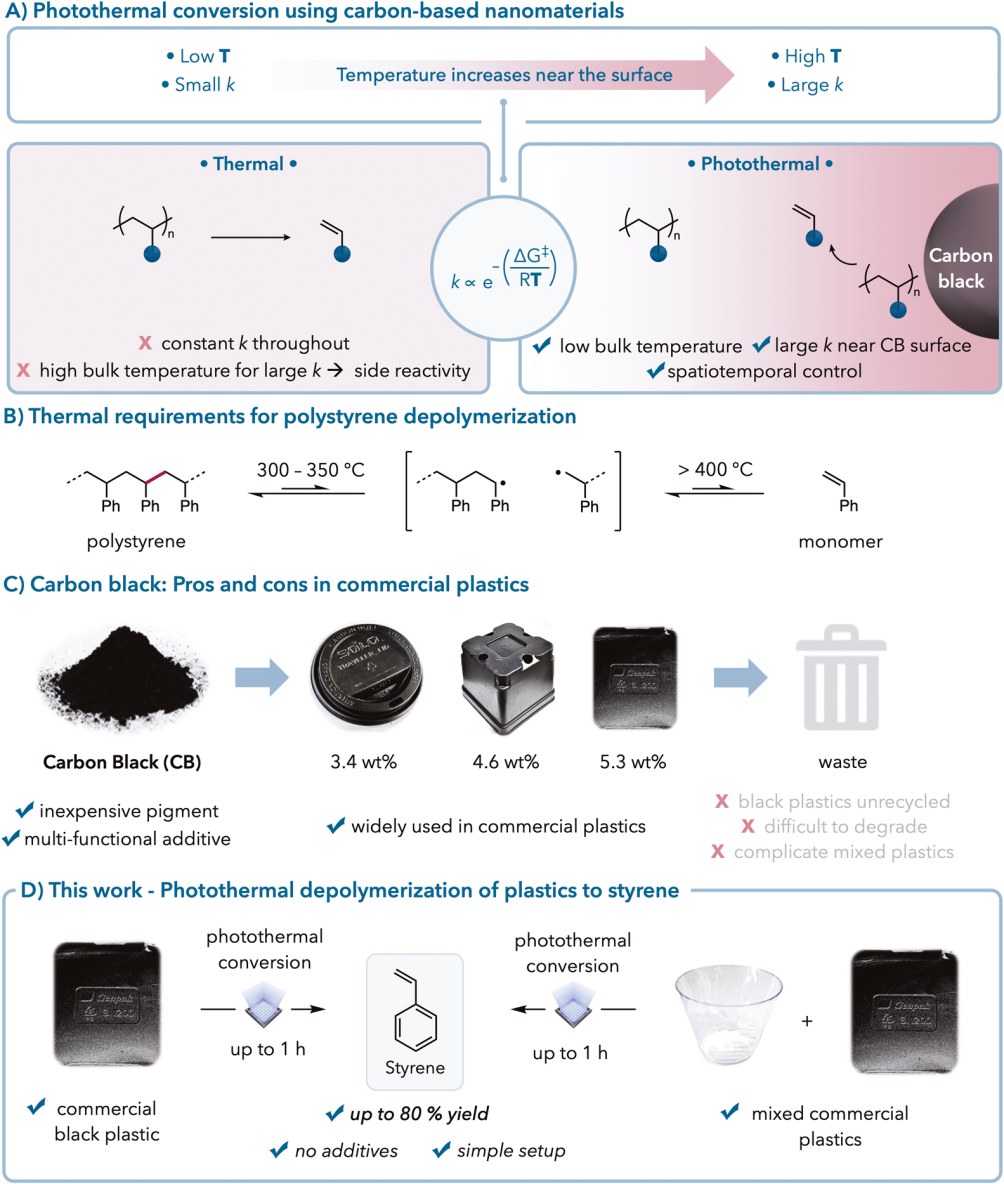
Using photothermal conversion to overcome existing recycling limitations. (A) Bulk heating versus photothermal conversion with localized heating. (B) Minimum polystyrene pyrolysis requirements. (C) Use of carbon black in existing plastics. (D) Photothermal conversion for chemical recycling to monomers of black and mixed commercial polystyrene plastics.

Photothermal conversion is a phenomenon where nanomaterials or chromophores can convert light to thermal energy.^1−6^ When irradiated, photothermal conversion agents are excited and undergo nonradiative decay, releasing heat localized to the nanoparticle surface while maintaining relatively low bulk temperatures.^7,8^ In contrast to bulk heating, where a rate constant is uniform across the reaction media, reaction rates are fastest near the particle surface and decrease with distance (Figure 1A).^9^ As a result, heat can be applied with spatial and temporal control, increasing reaction rates while providing highly selective reactions and avoiding byproduct formation.^1^

Heating under an inert atmosphere, or pyrolysis, has emerged as a strategy for chemical recycling to monomer (CRM) to alleviate plastic pollution.^10−25^ The increasing plastic production, short usage lifetimes of plastics, and their inherent chemical inertness have resulted in the massive accumulation of waste in landfills and the environment, causing tremendous environmental and health threats.^25−28^ For the chemical recycling of commercial plastics, high temperatures are required to surpass the degradation temperature (*T*_d_) of polymers and monomer ceiling temperature (*T*_c_), at which the rate of polymerization and depolymerization are equal.^29,30^ Upon homolytic chain cleavage, polymeric radicals afford monomers through depropagation at elevated temperatures (Figure 1B).^1−6^ This process usually requires bulk temperatures above 300 °C to achieve depolymerizations and often features competing side reactions.^13,14,26,31−34^ In comparison, photothermal conversion provides localized heating through simple light irradiation, where the systems reach high temperatures at the surface of the photothermal agents in picoseconds to nanoseconds but maintain lower bulk temperatures.^7^ Moreover, depolymerization mostly occurs near the photothermal agent surface under irradiation, while the temperature decreases exponentially as monomers are released from the polymer mixture, minimizing side reactions.

Photothermal conversion is an emergent approach to commercial plastic depolymerization due to the highly selective application of heat. However, challenges exist in identifying practical photothermal agents that are easily incorporated and reusable, hampering implementation.^1−6,35,36^ Carbon black is a suitable candidate to resolve these limitations, as it is an inexpensive carbon-based material and has been shown to promote photothermal conversion for organic reactions.^9,37−40^ Additionally, it is frequently used as a black pigment or an additive in black plastics (1–40%), enhancing the physical properties of commercial plastics.^27,41^ Historically, black plastics have presented a unique recycling challenge due to black filler materials that lead to ineffective sorting. With a recycling rate at nearly 0%, black plastics ultimately accumulate in landfills or undergo incineration, resulting in additional environmental pollution.^27^ Fortunately, efforts to enhance the sorting of black plastics have shown considerable progress, yet solutions for recycling methods remain largely unexplored.^41,42^ As a result, there is a lack of comprehensive recycling strategies as the pigment increases inertness to various stimuli (including mechanical force, light, and heat; Figure 1C).^27,41−43^

Herein, we demonstrate a unique strategy to mitigate black plastic pollution and facilitate a circular economy for polystyrene (PS). By taking advantage of existing photothermally active additives in black plastics, we use light to depolymerize commercial plastics back to monomers (Figure 1D). We first establish the feasibility of photothermal depolymerization of PS by carbon black using a model system of polystyrene–carbon black (PS-CB) composites with known carbon black concentrations. Subsequent visible light irradiation enables the depolymerization of black PS and styrene copolymers to monomers. Notably, our method proved effective for depolymerizing postconsumer waste black polystyrene plastics without additional carbon black or other catalysts. By leveraging the black pigment in plastics, we can also facilitate the depolymerization of black and nonblack materials. Photothermal depolymerization using existing plastic additives presents a new pathway for chemical recycling, allowing mild reaction conditions, lower overall energy costs, and high selectivity for monomer recovery.

## Results and Discussion

**Figure 2:**
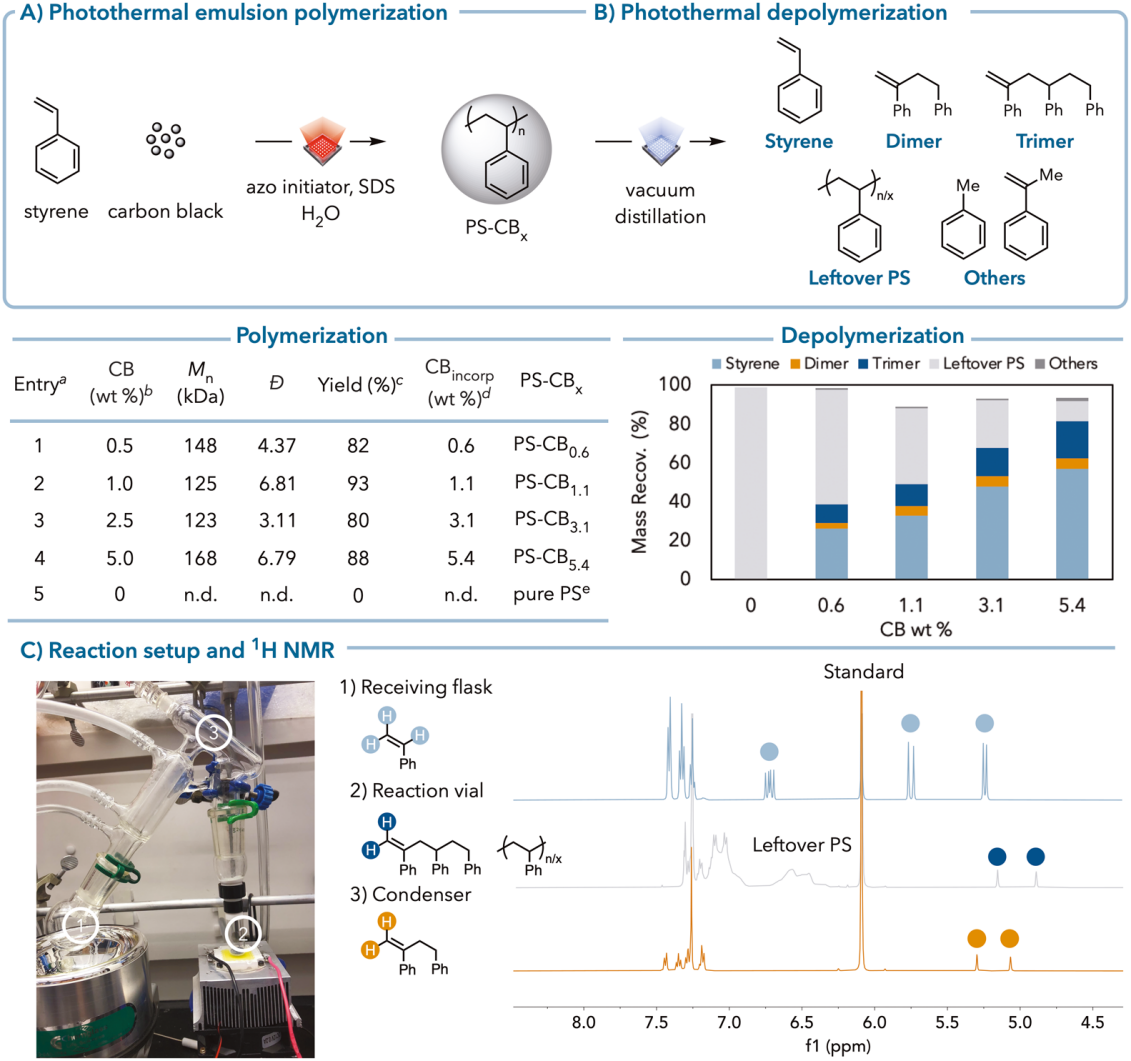
Photothermal polymerization and depolymerization. (A) Black polystyrene is synthesized via photothermal emulsion polymerization. (a) Detailed reaction conditions are provided in the Supporting Information. (b) CB wt % used in polymerization is calculated using the mass of CB/the mass of styrene. (c) Isolated yield of PS-CB_*x*_. (d) The calculation method for CB incorporated in PS is given in the Supporting Information. (e) Pure PS was synthesized via thermal emulsion polymerization at 80 °C. (B) PS-CB_*x*_ (50 mg) is photothermally depolymerized to small molecules. The calculation method for mass recovery is in the Supporting Information. The values are based on a styrene repeat unit basis. (C) (Left) Picture of photothermal depolymerization setup. (Right) ^1^H NMR spectrum of the receiving flask, reaction vial, and condenser after photothermal depolymerization of PS-CB_1.1_.

We first examined the feasibility of carbon black-mediated photothermal depolymerization in a controlled system without commercial additives beyond carbon black. We chose a commercially relevant emulsion polymerization method to prepare PS embedded with carbon black to easily access high molecular weight polymers.^44−47^ Because carbon black is an efficient photothermal agent, we hypothesized that low-intensity 660 nm LEDs could thermally activate the radical initiator for polymerization.^37,38,48^ We observed near quantitative styrene conversion to high molecular weight PS in the presence of 0.5 to 5 wt % carbon black (Figure 2A, entries 1–4). Pure PS was synthesized via thermal emulsion polymerization (Figure 2A, entry 5). By filtering the polymer precipitates, we obtained >80% yield of PS-CB material in all cases. Carbon black was recovered quantitatively with the polymer particles, providing the isolated polymer material ranging from 0.6 to 5.4 wt % carbon black (denoted PS-CB_*x*_ where *x* is the wt %, Figure 2A).

After synthesizing the PS-CB samples, we proceeded to investigate photothermal depolymerization studies. To achieve efficient depolymerization, local temperatures of carbon black should exceed the *T*_c_. To exceed 395 °C (*T*_c_ for styrene), we chose high-intensity white LED light to maximize heat transfer from carbon black to PS.^1^ We screened the PS-CB samples with different carbon black loadings to test the effect of weight loading on the photothermal depolymerization efficiency (Figure 2B). The primary product from depolymerization was styrene monomer, with dimer, trimer, toluene, and α-methylstyrene (AMS) also detected. We observed increasing amounts of styrene monomer with increasing carbon black wt %, reaching a maximum of 57% styrene monomer at 5 wt % loading (Figure 2B). Pure PS did not undergo depolymerization without carbon black under white LED irradiation, indicating that photothermal conversion facilitated the process. Notably, measured bulk temperatures did not exceed 150 °C, which is far below the *T*_d_ for polystyrene (see Supporting Information, Figure S119).

Analytically pure styrene, the dimer, and the trimer were successfully separated in the receiving flask, the condenser, and the reaction vial (Figure 2C). We assessed these samples with gas chromatography–mass spectrometry (GC-MS) to confirm the formation of all of the products (see Supporting Information, Figures S40–S47). We accounted for the loss of mass recovery to a small amount of styrene escaping the receiving flask and a minute amount of other byproducts. Despite these, after photothermal depolymerization, ^1^H NMR and GC-MS spectra showed a clean product distribution of the monomer, dimer, and trimer. After optimizing our photothermal depolymerization conditions, we demonstrated the recyclability of all system components (monomers and carbon black), supporting the circularity of our method (see Supporting Information, pages S62–S75).

**Figure 3:**
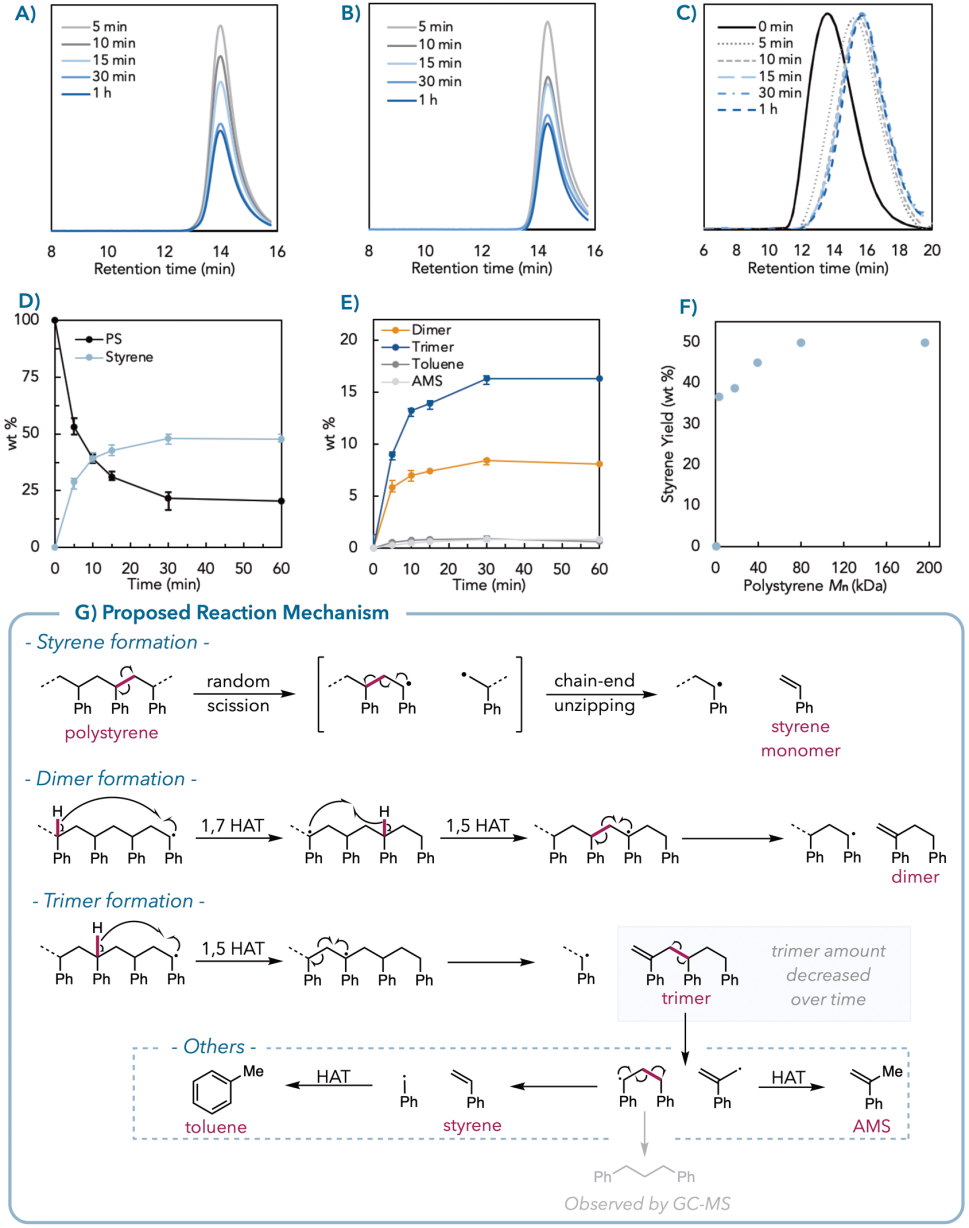
Photothermal depolymerization kinetics and proposed mechanism. Un-normalized GPC traces after photothermal depolymerization of (A) TEMPO-chain-end polystyrene and (B) de-TEMPO-chain-end polystyrene. (C) Normalized GPC traces of commercial polystyrene. (D,E) Scatter plot for commercial polystyrene–carbon black photothermal depolymerization products over time. (F) Styrene yields over different molecular weight polystyrene. (G) Proposed mechanism for polystyrene depolymerization.

To investigate the mechanism of polystyrene depolymerization in our system, we conducted time course studies on both lab-synthesized and commercial polystyrene. Using low molecular weight polystyrene (*M*_n_ = 18 kDa) with or without TEMPO chain ends, the leftover polystyrene molecular weight did not decrease throughout the reaction time despite the formation of the styrene monomer (Figure 3A,B). Commercial polystyrene (*M*_n_ = 84 kDa) displayed similar depolymerization properties where the molecular weight of the polymer decreased initially but then remained constant while the styrene yield increased (Figure 3C,D). Styrene dimers and trimers were formed simultaneously with styrene, indicating their formation from polystyrene rather than the oligomerization of styrene monomers (Figure 3E). Upon heating a mixture of trimers and dimers, the trimer amount decreased, while the α-methylstyrene (AMS), styrene, and toluene amounts increased, likely resulting from cleavage of the trimer (Figure 3G, see Supporting Information, Figure S140). To study the influence of molecular weight on photothermal depolymerization efficiency, we investigated polystyrene depolymerization with varied molecular weight (*M*_n_ = 0.5–196 kDa; Figure 3F). Larger polymers depolymerized more efficiently than smaller ones (also supported by the slightly skewed GPC traces in Figure 3C), which could be due to weaker C–C bonds from larger polystyrene and the increased entropy gain through depolymerization. Nonetheless, polystyrene with *M*_n_ greater than 2.7 kDa all depolymerized with a styrene yield of 37–50%, highlighting the applicability of the system over a broad range of molecular weights.

From these experiments, the mechanism of small molecule formation from polystyrene via photothermal depolymerization is shown in Figure 3G.^52^ We propose that photothermal depolymerization occurs through random chain scission of the polystyrene backbone followed by full depropagation (chain-end unzipping) to styrene monomers.^49−52^ This hypothesis is supported by nearly identical depolymerization behavior of TEMPO-terminated versus H-terminated polystyrene samples. Dimers and trimers arise from intramolecular hydrogen atom transfer (HAT) events concurrent with depropagation. Over time, the trimer is further cleaved to styrene, toluene, and AMS. This is further evidenced by the observation of 1,3-diphenyl propane in GC-MS.

**Figure 4:**
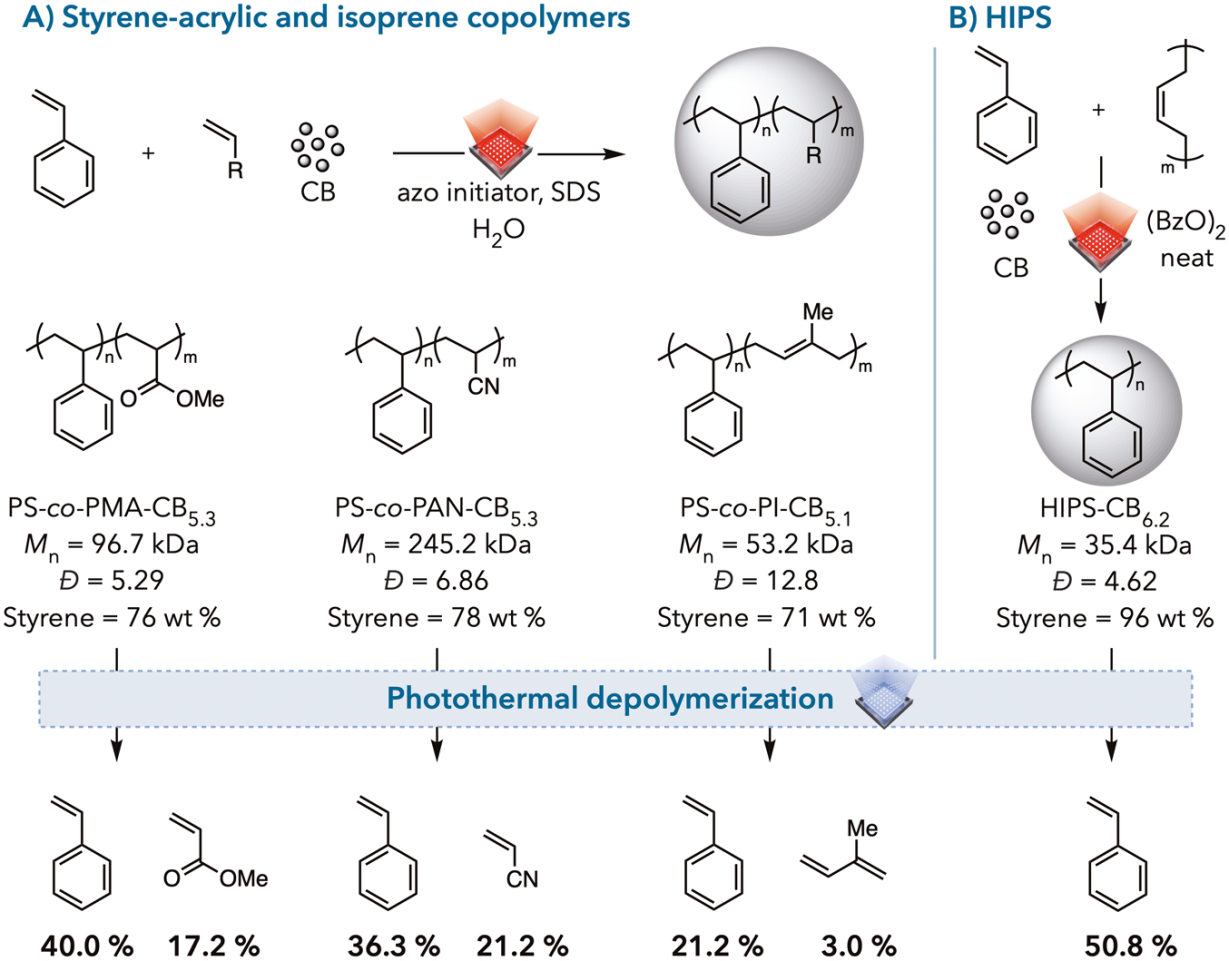
Synthesis and depolymerization of various copolymers. Detailed reaction conditions are provided in the Supporting Information for both synthesis and depolymerization. (A) PS-*co*-PMA-CB_5.3_, PS-*co*-PAN-CB_5.3_, and PS-*co*-PI-CB_5.1_ are synthesized via photothermal emulsion polymerization and depolymerized photothermally. (B) HIPS-CB_6.2_ is synthesized via photothermal bulk polymerization and depolymerized photothermally.

Styrene copolymers are of interest in depolymerization, as styrene is frequently used in copolymer synthesis to create new materials.^53−59^ We hypothesized that our system would be amenable to copolymer depolymerization. We first copolymerized styrene with methyl acrylate (PS-*co*-PMA-CB_5.3_), acrylonitrile (PS-*co*-PAN-CB_5.3_), and isoprene (PS-*co*-PI-CB_5.1_) via photothermal emulsion syntheses (Figure 4A). We also synthesized high impact polystyrene (HIPS-CB_6.2_) through graft copolymerization of styrene on polybutadiene (PB) by using photothermal bulk polymerization (Figure 4B).

We then subjected these copolymers to photothermal depolymerization for 1 h using the distillation protocol (Figure 4). A moderate amount of styrene and small amounts of comonomers, like methyl acrylate, acrylonitrile, and isoprene, were detected. We expected that copolymer depolymerization would be more challenging for the following reasons. Since these comonomers have higher ceiling temperatures, the copolymers are less likely to depropagate than pure polystyrene. Moreover, these copolymers would be more prone to undergo side reactions, especially for PS-*co*-PI-CB_5.1_ (with 71 wt % styrene), owing to the extra alkane and alkene chains on the backbone that other copolymers do not have. After hydrogenating PS-*co*-PI-CB_5.1_, we photothermally depolymerized and obtained a 20% styrene yield. The hydrogenation of the copolymer did not affect the styrene yield, indicating that double bonds did not necessarily hinder the efficiency of our photothermal depolymerization system. We also synthesized another PS-*co*-PI-CB_5.2_ (with 30 wt % styrene) and observed a 14% styrene yield after photothermal depolymerization (see Supporting Information, Table S23). Thus, the higher concentration of hydrocarbons on the backbone causes further reduction of styrene yield, possibly because these extra carbons engender more side reactions to prevent styrene formation. Despite these challenges, the overall results showed the potential for using photothermal depolymerization as a promising avenue for deconstructing styrene copolymers.

**Figure 5:**
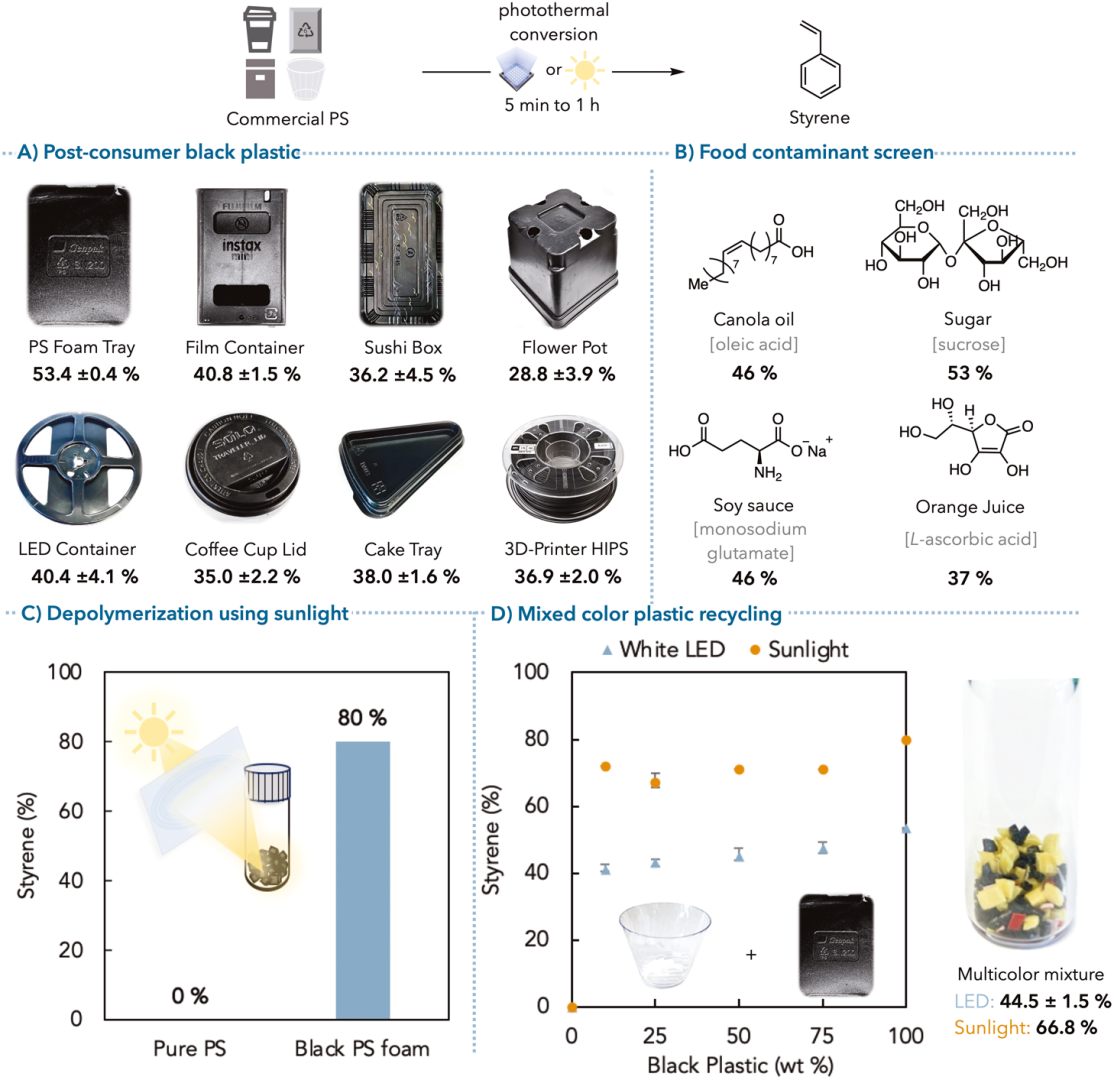
Photothermal depolymerization of commercial PS. Detailed product yield and molecular weight information are listed in the Supporting Information. (A) Black commercial PS samples (50 mg) are photothermally depolymerized with styrene % yield (average of three trials, see full data in the Supporting Information, Table S25). (B) Photothermal depolymerization of black PS foam in the presence of food contaminants (20 wt %) with styrene % yield. (C) Photothermal depolymerization is conducted under focused sunlight with styrene % yield. (D) Mixed-color postconsumer waste PS samples (100 mg) in different black PS ratios are photothermally depolymerized under intense white LED light or sunlight with styrene % yield (error bars from an average of three trials, see full data in the Supporting Information, Tables S33–S35).

Next, we demonstrated the applicability of our photothermal depolymerization method on commercial PS samples and its potential industrial value on postconsumer waste plastic recycling. Postconsumer black polystyrene samples, including foamed polystyrene (PS foam), food containers, coffee cup lids, etc., were subjected to our standard depolymerization conditions without additional carbon black or other metal catalysts. Excitingly, eight black polystyrene plastics were successfully depolymerized to styrene monomers in up to 53% yield (Figure 5A, see Supporting Information for additives wt % determination, Table S24 for a summary). Despite these materials containing variable amounts of carbon black or other additives, we obtained styrene yields > 30%, highlighting the generality of the approach and tolerance to variation. In addition, white or clear polystyrene samples were melt processed with carbon black to achieve 5 wt % carbon black films (see Supporting Information, Figure S114). Under the same depolymerization conditions, we observed similar styrene yields (up to 54%), comparable to the photothermal depolymerization of our as-synthesized PS-CB mixture (Table S26). These results indicated that carbon black could be added postuse to nonblack plastic samples and still perform efficient depolymerization. Furthermore, we performed depolymerization on 3 and 6 g scales of this material and achieved styrene yields of up to 44% (Tables S31).

Based on use, postconsumer PS wastes are often contaminated with food or other impurities.^60^ To mimic these conditions, we conducted photothermal depolymerization of black PS foam in the presence of various food contaminants (20 wt %) to test the robustness of our protocol. Gratifyingly, the monomer recovery of the sample with sucrose remained almost identical with the uncontaminated samples (Figure 5B). Canola oil and soy sauce slightly lowered the styrene yield to 46% (see Supporting Information, Table S28). Additionally, despite having a radical scavenger (vitamin C or ascorbic acid) that could quench radicals on the polymer backbone, the styrene recovery was still 37% in the presence of orange juice (see Supporting Information, Tables S28–S30).

With the successful depolymerization of commercial black plastics, we aimed to examine the potential of sunlight as the sole irradiation source. Using a Fresnel lens, we irradiated a commercial PS with focused sunlight under static vacuum. The black PS foam sample was fully depolymerized and yielded 80% styrene after 5 min, while the pure commercial PS powder stayed intact as a control group (Figure 5C, Table S32, entry 4). Compared with our LEDs, we attributed the higher reaction efficiency and completeness to the greater light intensity achieved by the focused sunlight.

Black plastics complicate recycling, as separating this material is required to recycle nonblack plastics.^27^ We demonstrated photothermal depolymerization of mixed colored postconsumer PS to illustrate that mixed plastic waste streams are compatible with our recycling strategy without separating black plastic (Figure 5D). As low as 10 wt % black PS foam was able to afford >40% styrene under LED irradiation, with increasing styrene yield with higher black plastic loading. We also obtained 44% styrene with a multicolored postconsumer PS mixture containing pieces of a red cup, yellow foam, clear cup, and black foam (Table S35). Ultimately, we subjected mixed colored postconsumer waste PS samples to focused sunlight irradiation, achieving 67–72% styrene yield with 10–75 wt % black plastic in just 5 min (Figure 5D, Table S33).

In conclusion, we successfully demonstrated photothermal conversion to polymerize and depolymerize polystyrene using carbon black as a photothermal conversion agent. We showcased efficient depolymerization using approximately 5 wt % carbon black loading with high-intensity white LEDs. Mechanistic experiments showed that photothermal depolymerization likely occurred via random chain scission and chain-end depropagation. We photothermally depolymerized existing commercial black polystyrene plastics, showing that our process tolerates a wide range of plastic additives and food contaminants. Finally, using sunlight as an energy source for photothermal conversion resulted in high depolymerization efficiencies. Our work addresses the poor recyclability of black plastics and is amenable to mixed plastic waste, and our mild yet powerful technique makes the circular economy of black plastics more viable.

## Supporting Information Available

Materials; methods; experimental details including polymer synthesis and depolymerization; data including GPC, NMR, GC-MS, TGA, DSC, XPS, and photographs of experimental setups (PDF)
30-times-speed recording of the first 5 min of the sunlight depolymerization of black PS foam (see Table S32, entry 3) (MP4)
120-times-speed recording of the 30 min photothermal depolymerization of commercial PS-CB5.0 (see Table S27, entry 4) (MP4)
