# Supplementary material for: Recycling of Post-Consumer Waste Polystyrene Using Commercial Plastic Additives
Source: ACS Cent Sci. 2024 Nov 25;11(1):57–65. doi: 10.1021/acscentsci.4c01317 (PMC11758496; doi:10.1021/acscentsci.4c01317)
Supplement: Supplementary file 1 — oc4c01317_si_001.pdf [file oc4c01317_si_001.pdf]

Supporting Information

Recycling of Post-Consumer Waste Polystyrene using Commercial Plastic Additives

Sewon Oh[1‡], Hanning Jiang[2‡], Liat H. Kugelmass[1], Erin E. Stache[2*]
Corresponding author: Erin E. Stache, estache@princeton.edu

‡S.O. and H.J. contributed equally to this work.
[1]Department of Chemistry and Chemical Biology, Cornell University; Ithaca, New York, USA.
[2]Department of Chemistry, Princeton University; Princeton, New Jersey, 08544, USA.

Pages S1 to S127
Figures S1 to S145
Tables S1 to S37

Table of Contents

Materials and Methods *S10*

General Procedure for Polystyrene Synthesis via Photothermal Conversion with Carbon Black *S12*

Figure S1. Reaction setup of photothermal emulsion polymerization. **S12**

Figure S2. Visual representation of each setup for photothermal emulsion polymerization. S13

Table S1. Results of photothermal emulsion polymerization. **S14**

Figure S3. GPC of PS-CB$_{5.4}$ **S14**

Figure S4. GPC of PS-CB$_{3.1}$ **S15**

Figure S5. GPC of PS-CB$_{1.1}$ **S15**

Figure S6. GPC of PS-CB$_{0.6}$ **S16**

Figure S7. ^{1}H NMR of PS-CB$_{5.4}$ polystyrene sample. **S16**

Procedure for 2 g Polystyrene Synthesis via Photothermal Conversion *S17*

Table S2. Results of photothermal emulsion polymerization (2g scale). **S17**

Figure S8. GPC of PS-CB$_{5.1}$ **S17**

Figure S9. GPC of PS-CB$_{5.5}$ **S18**

Procedure for Polystyrene Synthesis via Conventional Heating *S19*

Figure S10. GPC of pure PS. **S19**

Figure S11. Quantitative ^{1}H NMR of pure PS against 1,3,5-trimethoxybenzene. **S20**

Procedure for TEMPO-lated PS Synthesis via Conventional Heating *S21*

Figure S12. GPC of TEMPO-PS. **S21**

Figure S13. ^{1}H NMR of TEMPO-PS. **S22**

Procedure for Detempolation of TEMPO-lated PS *S23*

Figure S14. GPC of DeTEMPO-PS. **S23**

Figure S15. ^{1}H NMR of DeTEMPO-PS. **S24**

Procedure for Styrene Acrylonitrile Copolymer Synthesis via Photothermal Conversion *S25*

Figure S16. GPC of PS-*co*-PAN-CB$_{5.3}$. **S25**

Figure S17. ^{1}H NMR of PS-*co*-PAN-CB$_{5.3}$. **S26**

Procedure for Styrene Methyl Acrylate Copolymer Synthesis via Photothermal Conversion *S27*

Figure S18. GPC of PS-*co*-PMA-CB$_{5.3}$. **S27**

Figure S19. ^{1}H NMR of PS-*co*-PMA-CB$_{5.3}$ **S28**

Procedure for Styrene Isoprene Copolymer Synthesis via Photothermal Conversion *S29*

Figure S20. GPC of PS-*co*-PI-CB$_{5.1}$. **S29**

Figure S21. ^{1}H NMR of PS-*co*-PI-$CB_{5.1}$ S30

Procedure for Hydrogenation of Styrene Isoprene Copolymer *S31*

Figure S22. GPC of hydrogenated PS-*co*-PI-$CB_{6.6}$ S31

Figure S23. ^{1}H NMR of hydrogenated PS-*co*-PI-$CB_{6.6}$. S32

Figure S24. TGA of PS-*co*-PI (styrene = 71 wt %) before and after hydrogenation S32

Procedure for High Impact Polystyrene (HIPS) via Photothermal Conversion *S33*

Figure S25. GPC of HIPS-$CB_{6.2}$. S33

Figure S26. ^{1}H NMR of HIPS-$CB_{6.2}$. S34

Figure S27. Diffusion NMR of the mixture of pure PS and polybutadiene. S34

Figure S28. Stejskal–Tanner plot of pure PS and polybutadiene S35

Figure S29. Diffusion NMR of HIPS-$CB_{6.2}$. S35

Figure S30. Stejskal–Tanner plot of HIPS-$CB_{6.2}$ S36

Procedure for Photothermal Depolymerization under Air *S37*

Table S3. Results of photothermal depolymerization under air S37

Figure S31. GPC of photothermal depolymerization under air. S37

Figure S32. ^{1}H NMR of photothermal depolymerization under air. S38

Procedure for Photothermal Depolymerization under Nitrogen *S39*

Table S4. Results of PS-$CB_{1.1}$ after photothermal depolymerization under nitrogen. S39

Figure S33. GPC of PS-$CB_{1.1}$ after photothermal depolymerization under nitrogen S39

Figure S34. ^{1}H NMR of PS-$CB_{1.1}$ after photothermal depolymerization under nitrogen. S40

Procedure for Photothermal Depolymerization under Dynamic Vacuum *S41*

Figure S35. Reaction setup of photothermal depolymerization under vacuum S41

Table S5. Results of PS-$CB_{1.1}$ after photothermal depolymerization under vacuum. S42

Figure S36. GPC of PS-$CB_{1.1}$ after photothermal depolymerization under vacuum S42

Figure S37. ^{1}H NMR of PS-$CB_{1.1}$ after photothermal depolymerization under vacuum (reaction vial). S42

Figure S38. ^{1}H NMR of PS-$CB_{1.1}$ after photothermal depolymerization under vacuum (receiving flask). S43

Figure S39. ^{1}H NMR of PS-$CB_{1.1}$ after photothermal depolymerization under vacuum (condenser). S44

Figure S40. GC-MS of PS-$CB_{1.1}$ after photothermal depolymerization under vacuum (reaction vial). S44

Figure S41. Mass spectrum of Fig. S35 at 19.94 min. S45

Figure S42. GC-MS of PS-$CB_{1.1}$ after photothermal depolymerization under vacuum (receiving flask). S45

Figure S43. Mass spectrum of Fig. S37 at 3.02 min. S46

Figure S44. Mass spectrum of Fig. S37 at 5.32 min. S46
Figure S45. Mass spectrum of Fig. S37 at 7.02 min. S47
Figure S46. GC-MS of PS-$CB_{1.1}$ after photothermal depolymerization under vacuum (condenser). S47
Figure S47. Mass spectrum of Fig. S41 at 16.29 min. S48
Procedure for Carbon Black Loading for Photothermal Depolymerization *S49*
Table S6. Results of carbon black loading for photothermal depolymerization S49
Figure S48. GPC of PS-$CB_{0.6}$ after photothermal depolymerization. S49
Figure S49. GPC of PS-$CB_{3.1}$ after photothermal depolymerization. S50
Figure S50. GPC of PS-$CB_{5.4}$ after photothermal depolymerization. S50
Figure S51. GPC of PS-$CB_{5.4}$ + CB (10 wt %) after photothermal depolymerization. S51
Procedure for Metal Catalyst Test for Photothermal Depolymerization *S52*
Table S7. Results of metal catalyst test for photothermal depolymerization. S52
Figure S52. GPC of photothermal depolymerization with metal oxide. S52
Procedure for Control Experiments for Photothermal Depolymerization *S53*
Table S8. Results of controlled experiments for photothermal depolymerization. S53
Figure S53. GPC of controlled experiments after photothermal depolymerization. S53
Procedure for Pure PS & CB Mixture for Photothermal Depolymerization *S54*
Table S9. Results of Pure PS & CB mixture for photothermal depolymerization. S54
Figure S54. GPC of Pure PS & CB mixture after photothermal depolymerization. S54
Procedure for Time Course Study for Photothermal Depolymerization *S55*
Table S10. Results of time course study for photothermal depolymerization. S55
Figure S55. GPC of PS-$CB_{5.5}$ after 5 min photothermal depolymerization. S56
Figure S56. GPC of PS-$CB_{5.5}$ after 10 min photothermal depolymerization. S56
Figure S57. GPC of PS-$CB_{5.5}$ after 15 min photothermal depolymerization. S57
Figure S58. GPC of PS-$CB_{5.5}$ after 30 min photothermal depolymerization. S57
Figure S59. GPC of PS-$CB_{5.5}$ after 60 min photothermal depolymerization. S58
Figure S60. Scatter plot for the results of kinetic study for photothermal depolymerization. S58
Procedure for TEMPO-PS for Photothermal Depolymerization *S59*
Table S11. Results of TEMPO-PS for photothermal depolymerization. S59
Figure S61. GPC of TEMPO-PS after photothermal depolymerization. S59
Procedure for DeTEMPO-PS for Photothermal Depolymerization *S60*
Table S12. Results of DeTEMPO-PS for photothermal depolymerization. S60
Figure S62. GPC of DeTEMPO-PS after photothermal depolymerization. S60

Procedure for Larger Scale Photothermal Depolymerization *S61*

Table S13. Results of photothermal depolymerization with 1 g scale. S61

Figure S63. GPC of PS-$CB_{5.1}$ (1 g) after photothermal depolymerization. S61

Procedure for Repurposing Leftover PS-CB After Depolymerization *S62*

Table S14. Results of repurposing leftover PS-CB. S62

Figure S64. GPC of PS_r-$CB_{r0.6}$. S62

Figure S65. GPC of repurposed PS_r-$CB_{r5.6}$. S63

Figure S66. TGA of repurposed PS_r-CB_{rx}. S63

Figure S67. DSC of repurposed PS_r-CB_{rx}. S64

Procedure for Depolymerizing Repurposed PS_r-CB_{rx} *S65*

Table S15. Results of photothermal depolymerization with repurposed PS_r-CB_{rx}. S65

Figure S69. GPC of PS_r-$CB_{r0.6}$ after photothermal depolymerization. S65

Figure S70. GPC of repurposed PS_r-$CB_{r5.6}$ after photothermal depolymerization. S66

Procedure for Photothermal Depolymerization with Recycled Carbon Black *S67*

Table S16. Results of polymerization with recycled CB. S68

Table S17. Results of photothermal depolymerization with recycled CB. S68

Figure S71. GPC of PS-CB_1 after photothermal depolymerization. S68

Figure S72. GPC of PS-CB_2 after photothermal depolymerization. S69

Figure S73. GPC of PS-CB_3 after photothermal depolymerization. S69

Figure S74. GPC of PS-CB_4 after photothermal depolymerization. S70

Figure S75. GPC of PS-CB_5 after photothermal depolymerization. S70

Figure S76. Isolated styrene yield after photothermal depolymerization at each CB cycle. . S71

Figure S77. TGA of PS-CB_x (x is the number of times CB is used). S71

Figure S78. DSC of PS-CB_x (x is the number of times CB is used). S72

Procedure for Photothermal Depolymerization with Aluminum Foil Control *S73*

Table S18. Results of photothermal depolymerization with aluminum foil control. S73

Figure S79. GPC of PS-CB_1 after photothermal depolymerization with aluminum foil control. S73

Procedure for Photothermal Depolymerization with Recovered Styrene *S74*

Table S19. Results of photothermal depolymerization with recovered styrene. S74

Figure S80. GPC of PS_r-$CB_{6.0}$ after photothermal depolymerization. S74

Figure S81. TGA of PS_r-$CB_{6.0}$. S75

Figure S82. DSC of PS_r-$CB_{6.0}$. S75

Procedure for HIPS-CB Photothermal Depolymerization *S76*

Table S20. Results of photothermal depolymerization with HIPS-$CB_{6.2}$. S76

Figure S83. GPC of HIPS-$CB_{6.2}$ after photothermal depolymerization.................................. S76

Procedure for PS-co-PAN-CB Photothermal Depolymerization *S77*

Table S21. Results of photothermal depolymerization with PS-*co*-PAN-$CB_{5.3}$. S77

Figure S84. GPC of PS-*co*-PAN-$CB_{5.3}$ after photothermal depolymerization......................... S77

Figure S85. Plausible structures based on GC-MS and ^{1}H NMR studies............................... S78

Procedure for PS-co-PMA Photothermal Depolymerization *S79*

Table S22. Results of photothermal depolymerization with PS-*co*-PMA-$CB_{5.3}$. S79

Figure S86. GPC of PS-*co*-PMA-$CB_{5.3}$ after photothermal depolymerization. S79

Procedure for PS-co-PI-CB Photothermal Depolymerization *S80*

Table S23. Results of photothermal depolymerization with PS-*co*-PI-CB............................ S80

Figure S87. GPC of PS-*co*-PI-$CB_{5.2}$ (styrene 30 wt %) after photothermal depolymerization. S80

Figure S88. GPC of PS-*co*-PI-$CB_{5.1}$ (styrene 71 wt %) after photothermal depolymerization. S81

Figure S89. GPC of hydrogenated PS-co-PI-$CB_{6.6}$ after photothermal depolymerization. ... S81

Procedure for Determining the Percent Purity of PS in Post-consumer Waste PS Samples . *S82*

Table S24. Sample purity and molecular weight information of all commercial PS samples. S82

Figure S90. ^{1}H NMR of black PS foam tray. S82

Figure S91. ^{1}H NMR of film container.. S83

Figure S92. ^{1}H NMR of sushi box. S83

Figure S93. ^{1}H NMR of flower pot. S84

Figure S94. ^{1}H NMR of LED container. S84

Figure S95. ^{1}H NMR of black coffee cup lid. S85

Figure S96. ^{1}H NMR of cake tray. S85

Figure S97. ^{1}H NMR of 3D-printer HIPS. S86

Figure S98. ^{1}H NMR of white coffee lid. S86

Figure S99. ^{1}H NMR of white PS foam. S87

Figure S100. ^{1}H NMR of clear PS container lid. S87

Figure S101. ^{1}H NMR of clear PS cup.. S88

Figure S102. ^{1}H NMR of pure commercial PS from Aldrich (M_w = 192 kDa). S88

Figure S103. ^{1}H NMR of red PS cup. S89

Figure S104. ^{1}H NMR of yellow PS foam tray.. S89

Procedure for Further Characterization of Post-consumer Waste PS Samples *S90*

Figure S105. GPC of post-consumer waste black polystyrene samples. S90

Figure S106. GPC of post-consumer waste non-black polystyrene samples. S91

Figure S107. TGA of post-consumer waste black PS samples. S91

Figure S108. TGA of post-consumer waste white PS foam. S92

Figure S109. TGA of pure commercial PS from Aldrich (M_w = 192 kDa). S92

Figure S110. XPS of isolated black solid mixture from black PS foam tray. S93

Figure S111. XPS of isolated black solid mixture from film container. S93

Figure S112. XPS of isolated black solid mixture from black coffee lid. S94

Procedure for Post-Consumer Waste Black PS Photothermal Depolymerization *S95*

Table S25. Results of photothermal depolymerization with post-consumer waste black PS. S95

Figure S113. GPC of post-consumer waste black PS after photothermal depolymerization. S96

Procedure for Post-consumer Waste Non-Black PS Photothermal Depolymerization *S97*

Figure S114. Visual representation of each setup for recycling post-consumer waste PS. S97

Table S26. Results of photothermal depolymerization with post-consumer waste non-black PS. S98

Figure S115. GPC of post-consumer waste non-black PS after photothermal depolymerization. S98

Figure S116. Bar chart of additives wt % and styrene yield of post-consumer waste PS. S99

Procedure for Time Course Study for Commercial PS Photothermal Depolymerization *S100*

Table S27. Results of commercial PS-$CB_{5.0}$ time course study for photothermal depolymerization S100

Figure S117. GPC of un-normalized commercial PS-$CB_{5.0}$ time course. S100

Figure S118. Scatter plot for the results of kinetic study for commercial PS-$CB_{5.0}$ photothermal depolymerization S101

Figure S119. Bulk temperature of commercial PS-$CB_{5.0}$ photothermal depolymerization in 1 hour measured using thermocouple. S101

Figure S120. XPS of carbon black (from Alfa Aesar) isolated after photothermal reaction on PS-$CB_{5.0}$. S102

Procedure for Contaminated Black PS foam Photothermal Depolymerization *S103*

Figure S121. Picture for food contaminants used (canola oil/ sugar/ soy sauce/ orange juice). S103

Table S28. Results of 20 wt % contaminants study on black PS foam tray photothermal depolymerization S103

Figure S122. GPC of 20 wt % contaminants before and after photothermal depolymerization. S104

Table S29. Results of 100 wt % contaminants study on black PS foam tray photothermal depolymerization S104

Table S30. **Results of *L*-ascorbic acid study on black PS foam tray photothermal depolymerization** **S104**

Procedure for Multigram Scale Post-Consumer Waste PS Photothermal Depolymerization ***S105***

Figure S123. **Reaction setup of multigram post-consumer waste PS-CB composite photothermal depolymerization.** **S105**

Figure S124. **Reaction photos of multigram post-consumer waste PS-CB composite photothermal depolymerization (3 g before/ after, 6 g before/ after).** **S106**

Table S31. **Results of multigram post-consumer waste white PS photothermal depolymerization** **S106**

Figure S125. **¹H NMR of photothermal depolymerization of 3 g mixed post-consumer waste white PS-CB composite (receiving flask).** **S107**

Figure S126. **¹H NMR of photothermal depolymerization of 3 g mixed post-consumer waste white PS-CB composite (reaction vial).** **S107**

Figure S127. **¹H NMR of photothermal depolymerization of 3 g mixed post-consumer waste white PS-CB composite (condenser).** **S108**

Figure S128. **GPC of 3 g mixed post-consumer waste white PS-CB composite after photothermal depolymerization.** **S108**

Procedure for Photothermal Depolymerization with Focused Sunlight ***S109***

Figure S129. **Picture for focused sunlight photothermal depolymerization setup** **S109**

Table S32. **Results of black PS foam tray after focused sunlight photothermal depolymerization** **S110**

Figure S130. **GPC of black PS foam tray after focused sunlight photothermal depolymerization after 5 minutes (Table S32, entry 1)** **S110**

Figure S131. **GPC of commercial pure PS from Aldrich (M_w = 192 kDa) after focused sunlight photothermal depolymerization (Table S32, entry 4).** **S111**

Figure S132. **¹H NMR of photothermal depolymerization of black PS foam tray after focused sunlight irradiation (Table S32, entry 1).** **S111**

Figure S133. **¹H NMR of photothermal depolymerization of commercial pure polystyrene (M_w = 192 kDa) under focused sunlight irradiation (Table S32, entry 4).** **S112**

Table S33. **Results of mixed color post-consumer waste polystyrene after focused sunlight photothermal depolymerization.** **S112**

Procedure for Black and Clear Mixed Post-Consumer Waste PS Photothermal Depolymerization ***S113***

Table S34. **Results of black and clear mixed post-consumer waste PS photothermal depolymerization** **S113**

Figure S134. **GPC of black and clear mixed post-consumer waste PS before and after photothermal depolymerization.** **S114**

Procedure for Multicolor Post-Consumer Waste PS Photothermal Depolymerization ***S115***

Figure S135. **Reaction photos of multicolor post-consumer waste PS samples before and after photothermal depolymerization.** **S115**

Table S35. **Results of multicolor post-consumer waste PS photothermal depolymerization. S115**

Figure S136. **GPC of multicolor PS samples before and after photothermal depolymerization. S116**

Procedure for Thermal Study for Commercial PS ***S117***

Figure S137. **Scatter plot for the results of thermal depolymerization (360-420°C) under static vacuum after 30 minutes.** **S117**

Figure S138. **Scatter plot for the results of thermal depolymerization (420-500 °C) under static vacuum after 5 minutes.** **S118**

Figure S139. **^{1}H NMR of depolymerization of PS-$CB_{5.0}$ under 420 °C after 30 min.** **S118**

Procedure for Photothermal Decomposition of Dimer and Trimer Mixture ***S119***

Table S36. **Results of photothermal decomposition of dimer and trimer mixture.** **S119**

Figure S140. **^{1}H NMR of photothermal decomposition of dimer and trimer mixture.** **S120**

Procedure for Different Molecular Weight PS Photothermal Depolymerization ***S121***

Table S37. **Results of photothermal decomposition of dimer and trimer mixture.** **S121**

Photon Flux Calculation using Chemical Actinometry ***S122***

Figure S141. **Iron phenanthroline ($Fe(phen)_3$) standard concentration plot** **S122**

Figure S142. **Stacked UV-Vis spectrum of actinometry solution upon irradiation with high-intensity white LED (2.85 W/cm^2, 6000 k) over time** **S123**

Figure S143. **Fe(II) (complexed as $Fe(phen)_3$) concentration in actinometry solution upon irradiation with high-intensity white LED (2.85 W/cm^2, 6000 k) over time.** **S123**

Figure S144. **Stacked UV-Vis spectrum of actinometry solution with 2.5 mg carbon black upon high-intensity white LED (2.85 W/cm^2, 6000 k) irradiation over time.** **S124**

Figure S145. **Fe(II) (complexed as $Fe(phen)_3$) concentration in actinometry solution with 2.5 mg carbon black upon irradiation with high-intensity white LED (2.85 W/cm^2, 6000 k) over time. S124**

Movies ***S125***

References ***S126***

Materials and Methods

General Reagent Information

Styrene (99%, Sigma Aldrich), acrylonitrile (99%, Sigma Aldrich), isoprene (99%, Sigma Aldrich), and methyl acrylate (MA) (99%, Acros Organics) were filtered through a pack of aluminum oxide (activated, basic, Brockman Graded I, Oakwood Chemical) to remove any stabilizers. Carbon black (amorphous, acetylene 100 % compressed, Alfa Aesar), sodium dodecyl sulfate (SDS) (97 %, Oakwood Chemical), 4,4′-azobis(4-cyanovaleric acid) (ABCVA) (98%, Fluka Chemical), benzoyl peroxide ($(BzO)_2$) (97%, Sigma Aldrich), 1,3,5-trimethoxybenzene (98%, TCI), butylated hydroxytoluene (BHT) (99%, Oakwood), *p*-toluenesulfonyl hydrazide (97%, Sigma Aldrich), polystyrene (M_w = 192 kDa, Sigma Aldrich), polystyrene standard (M_w = 500 Da, Scientific Polymer Products Inc.), polybutadiene (PB) (75 % cis, M_n = 3000 Da, Sigma Aldrich), zinc oxide (ZnO) (<100 nm, Sigma Aldrich), barium oxide (BaO) (97 %, Sigma Aldrich), calcium oxide (CaO) (lab grade, Ward's Science), magnesium oxide (MgO) (analytical reagent, Mallinckrodt), and hydrochloric acid (HCl) (GR ACS 37 %, Sigma Aldrich) were used without any purification. Acetone (ACS 99.5%, Fisher Scientific), methanol (ACS 99.8%, Fisher Scientific), dichloromethane (ACS 99.5%, Fisher Scientific), tetrahydrofuran (99% ACS AR, Macron Fine Chemicals), hexane (ACS 98.5%, Fisher Scientific), xylene (ACS 98.5%, J. T. Baker), toluene (ACS, Fisher Scientific), and deuterated chloroform (99.8%, Cambridge Isotope Laboratories Inc.) were used as received. Styrene unimer was prepared with our previous method.[1] All synthesized polymer samples (except for styrene isoprene copolymer) were finely grounded before depolymerization. 660 nm LED (1.43 W/cm^2), was used for polymerization and low-intensity white LED (2.57 W/cm^2, 6000k) and high-intensity white LED (2.85 W/cm^2, 6000 k) were used for depolymerization. Plastic Fresnel lens (21.1 cm x 29.8 cm) was used to focus sunlight, with focal area 1.77 cm^2 and focused intensity ranging from 23.7 W/cm^2 (January 2024) – 31.9 W/cm^2 (May 2024). Post-consumer commercial polystyrene samples were collected from cafés, restaurants, and markets. Canola oil (Great Value Canola Oil, Walmart), sugar (Great Value Confectioners Powdered Sugar, Walmart), soy sauce (Lee Kum Kee Premium Soy Sauce), L-ascorbic acid (99%, Sigma Aldrich) and orange juice (Trader Joe's California Stem & Leaf, freshly squeezed) were used for contaminant studies.

Characterization Methods

Gel-Permeation Chromatography (GPC) – Number-average molecular weights (M_n) and dispersities ($Đ$, where $Đ = M_w/M_n$) of all lab-made polymer samples were analyzed using a Tosoh EcoSEC HLC 8320 system with two SuperHM-M columns in series at a flow rate of 0.350 mL/min. Tetrahydrofuran was used as the eluent, and the sample data were determined against 12 TSKgel polystyrene standards. For all commercial sample measurements, Agilent 1260 Infinity II system was used. The system was built with Agilent 1260 Infinity II Isocratic Pump, with 1260 Infinity II Refractive Index detector using two SDV linear M columns (8 × 300 mm, 3 µm) with one SDV guard column (8 × 50 mm, 3 µm). Tetrahydrofuran (THF; VWR Chemicals BDH, 99%) stabilized with BHT was used as the eluent at a flow rate of 1 ml/min and temperature of 35 °C. The Agilent GPC system was also calibrated by polystyrene standards.

Nuclear Magnetic Resonance (NMR) – Bruker 400 and 500 MHz NMR instrument were used to obtain ^{1}H and Diffusion ^{1}H NMR spectra at room temperature using $CDCl_3$. For analyzing the quantities of small molecules after depolymerization, 1,3,5-trimethoxybenzene was used as an external standard.

Gas Chromatography-Mass Spectrometry (GC-MS) – GC-MS was equipped with an Agilent J&W capillary column (30 m x 250 μm x 0.25 μm) with helium as a carrier gas. The column temperature started at 50 °C for 3 min, was raised to 150 °C with the ramp rate of 10 °C/min. and was raised to 300 °C with the ramp rate of 20 °C and held at 300 °C for 1 min. Electron Ionization (EI) was used for mass spectrometry (5977B MSD).

Thermogravimetric Analysis (TGA) – A PerkinElmer TGA-8000 was used as the thermogravimetric analyzer to measure the amount of black dye in commercial PS. The samples were heated from 30 ˚C with a ramp rate of 20 °C/min, and held at 500 or 600 ˚C for 1 min.

Differential Scanning Calorimetry (DSC) – A DSC Auto 2500 from TA Instruments was used to measure glass transition temperatures of polymers. Two heating and one cooling cycles (ranging from 20 to 160 °C) were conducted with both heating and cooling rate of 10 °C/min under nitrogen as purge gas. The first heating and cooling cycles were used to eliminate the thermal history, and the second heating cycle to record glass transition temperatures.

X-Ray Photoelectron Spectrometer (XPS) – ThermoFisher K-Alpha XPS was used for elemental analysis of post-consumer PS.

Calculation for CB % Incorporation after Photothermal Emulsion Polymerization

We made two assumptions before calculating CB % incorporation in polystyrene after photothermal emulsion polymerization. 1). After polymerization, CB is quantitatively recovered because CB is insoluble in solvents and very well filtered after vacuum filtration (see Fig. S2). 2). The resulting PS-CB consisted of only polystyrene and carbon black, with SDS removed during filtration. We synthesized pure PS through thermal emulsion polymerization and discovered >99% purity of PS through quantitative ^{1}H NMR spectrum against an internal standard (see Fig. S13).

$$CB\ incorporation\ \% = \frac{initial\ mass\ of\ CB}{mass\ of\ isolated\ PS\text{–}CB} 100\%$$

Calculation Method for Depolymerization

The following equation was used for calculating the mole % of the small molecules after polystyrene depolymerization via ^{1}H NMR:

$$mol\ \% = \frac{a \times b}{c} 100\%$$

a = the mole of 1,3,5-trimethoxybenzene

b = the ratio of the moles of small molecule to 1,3,5-trimethoxybenzene

c = the mole of styrene units in polystyrene (without carbon black mass)

Calculation for Mass Recovery after Photothermal Depolymerization

We used ^{1}H NMR to calculate the % in styrene repeating unit (RPU) of styrene, dimer, trimer, toluene, alpha-methylstyrene (AMS) and leftover PS (see Calculation Method for Depolymerization) and added altogether.

$$Mass\ Recov. = styrene\% + dimer\% + trimer\% + toluene\% + AMS\% + leftover\ PS\%$$

General Procedure for Polystyrene Synthesis via Photothermal Conversion with Carbon Black

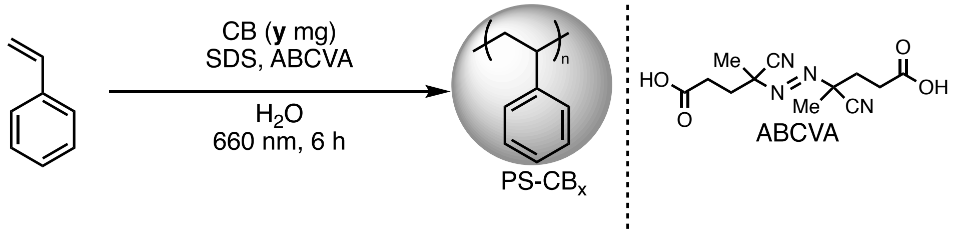

The procedure was modified from the literature.[2] Deionized H_2O (10 mL) was added into a scintillation vial, sealed with a cap equipped with a Teflon septum, and sparged with N_2 for 25 min. While waiting, styrene was degassed under N_2 for 20 min. ABCVA (0.01 mmol, 3 mg, 1 equiv.), SDS (0.76 mmol, 208 mg, 13.2 equiv.), carbon black (**y** mg) were added into the scintillation vial. While stirring the aqueous mixture, degassed styrene (10.0 mmol, 1.15 mL, 1.035 g, 1000 equiv.) was added. The entire mixture was irradiated with 660 nm LED light and stirred for 6 hours. The sample was removed from the light source and cooled to room temperature. The polymer was precipitated using cold MeOH (150 mL). The polymer precipitates were collected through vacuum filtration (repeat filtration one more time if necessary), followed by washing with a copious amount of methanol. The polymer was dried under high vacuum overnight. GPC and ^1H NMR spectra of polystyrene were shown below.

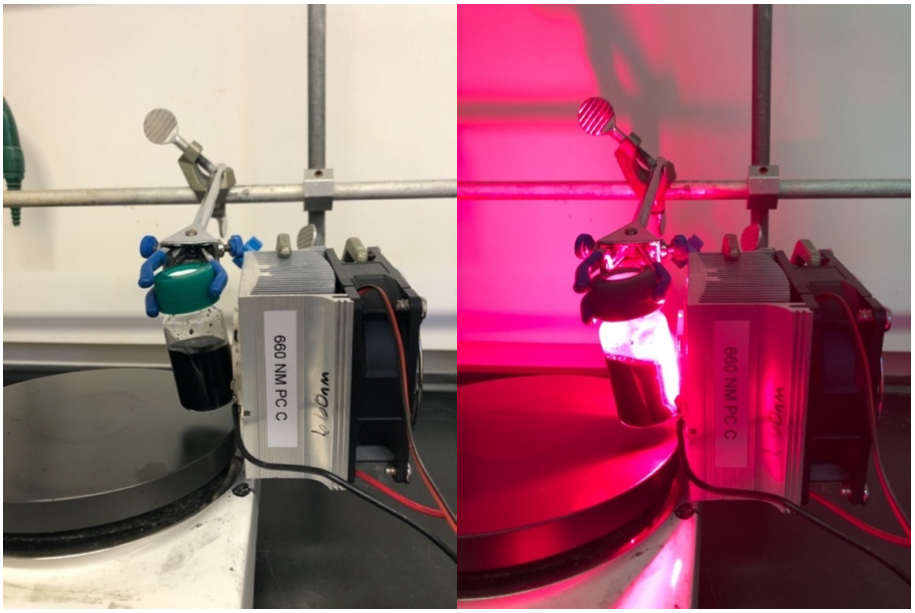

Figure S1. Reaction setup of photothermal emulsion polymerization.

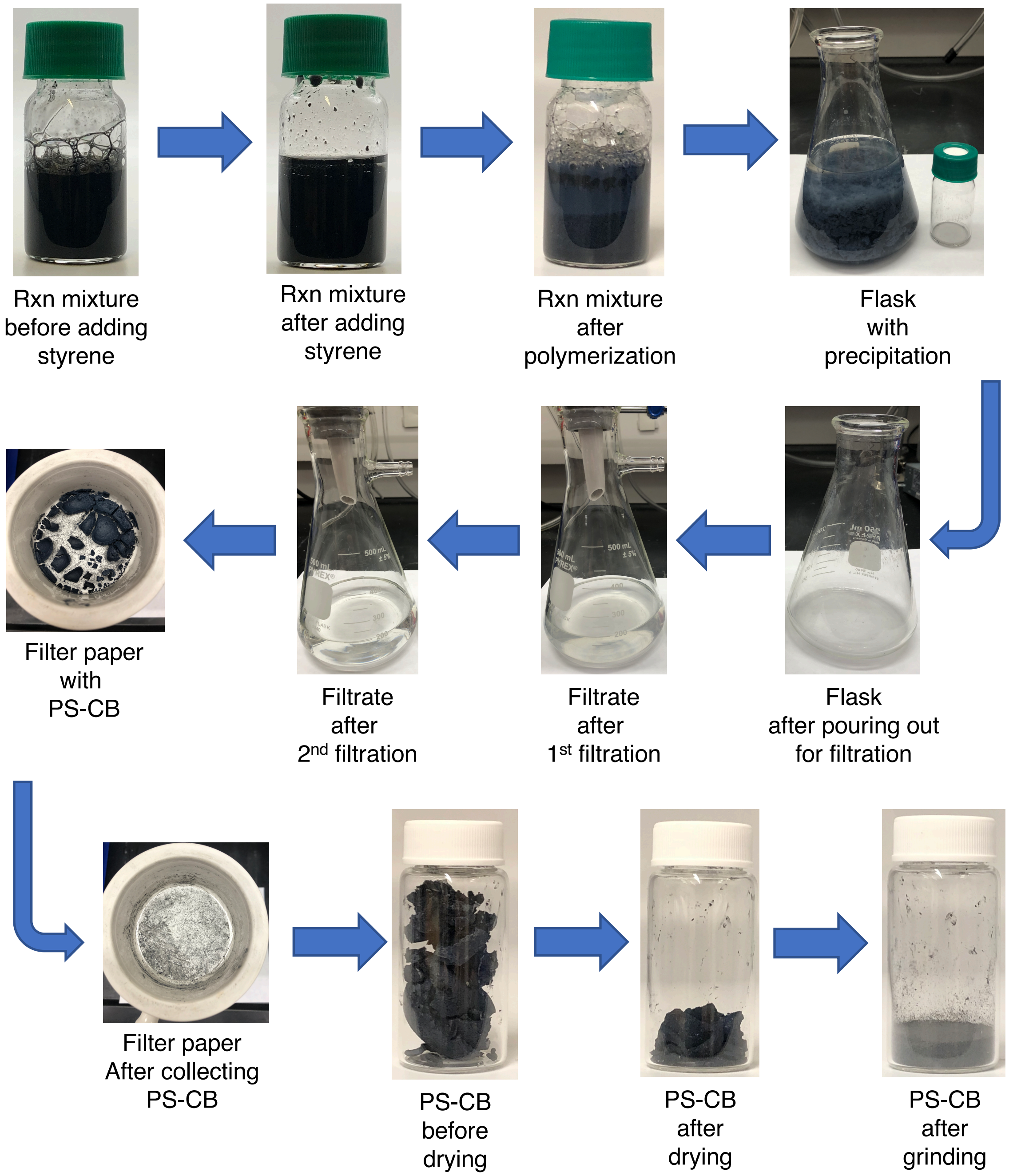

Figure S2. Visual representation of each setup for photothermal emulsion polymerization.

Table S1. Results of photothermal emulsion polymerization.

Entry	**y** (mg)	M_n (kDa)	*Đ*	Yield (%)[a]	CB_{incorp} (wt%)	$PS\text{-}CB_x$
1	51.8	167.8	6.79	88	5.4	$PS\text{-}CB_{5.4}$
2	25.9	122.5	3.11	80	3.1	$PS\text{-}CB_{3.1}$
3	10.4	125.4	6.81	93	1.1	$PS\text{-}CB_{1.1}$
4	5.2	147.8	4.37	82	0.6	$PS\text{-}CB_{0.6}$
5	0	n.d.	n.d.	0	n.d.	n.d.

[a]Calculated by isolated product mass / (initial styrene mass + initial CB mass)

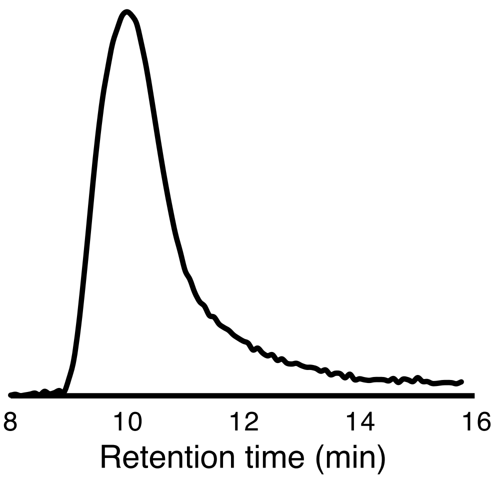

Figure S3. GPC of $PS\text{-}CB_{5.4}$.

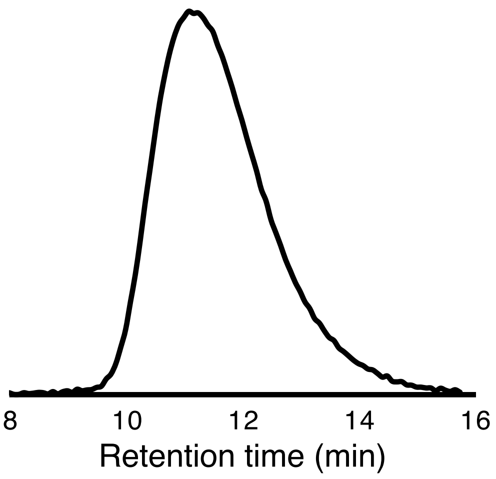

Figure S4. GPC of PS-$CB_{3.1}$.

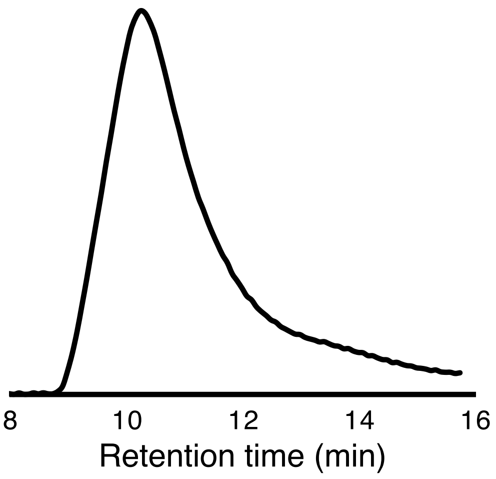

Figure S5. GPC of PS-$CB_{1.1}$.

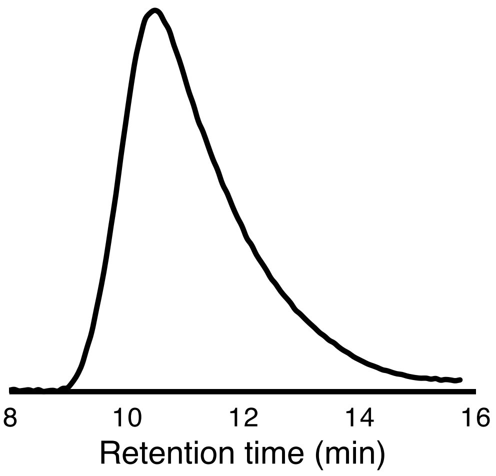

Figure S6. GPC of PS-$CB_{0.6}$.

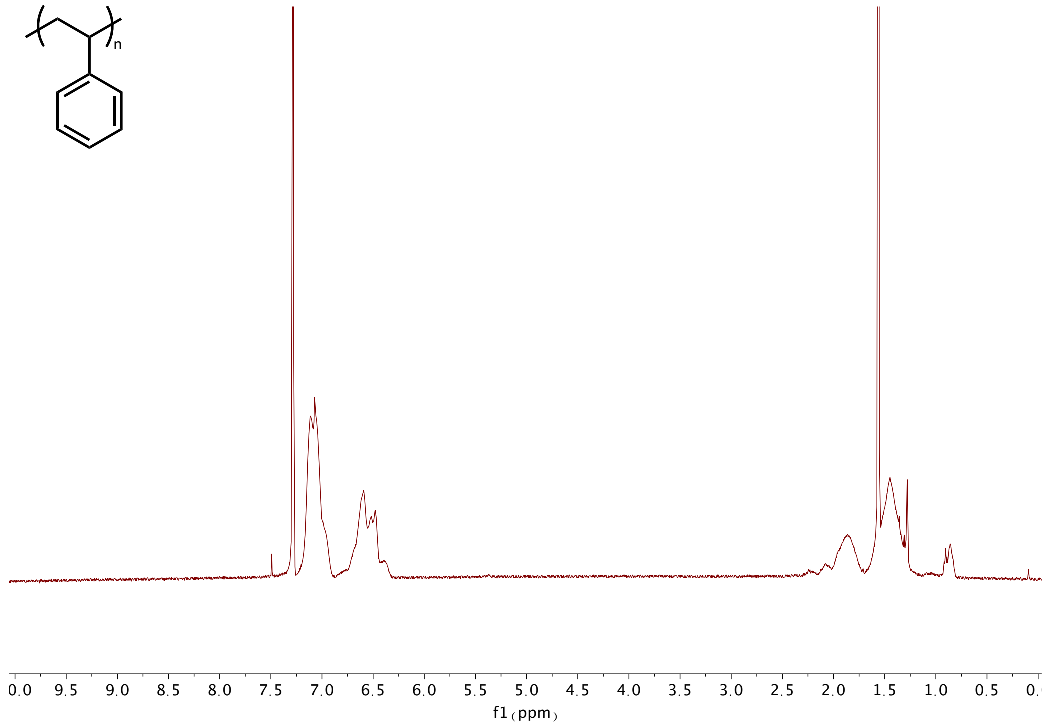

Figure S7. ^{1}H NMR of PS-$CB_{5.4}$ polystyrene sample.

Procedure for 2 g Polystyrene Synthesis via Photothermal Conversion

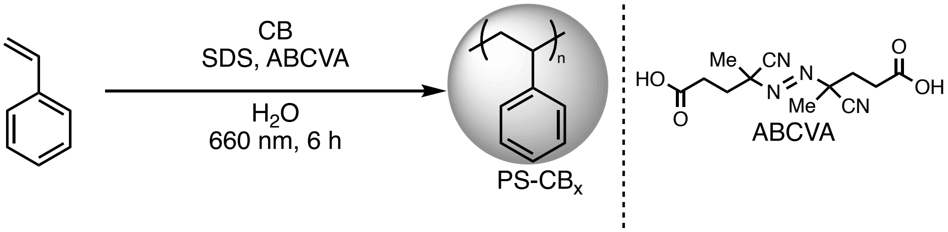

The procedure was slightly modified from the general procedure of polystyrene synthesis via photothermal conversion with carbon black. Two identical samples with carbon black (51.8 mg, each) were prepared, polymerized, and precipitated separately. Two samples were mixed upon vacuum filtration. GPC of polystyrene was shown below.

Table S2. Results of photothermal emulsion polymerization (2g scale).

Entry	M_n (kDa)	$Đ$	Yield (%)[a]	CB$_{incorp}$ (wt%)	PS-CB$_x$
1	109.8	4.62	94	5.1	PS-CB$_{5.1}$
2	136.6	5.95	86	5.5	PS-CB$_{5.5}$

[a]Calculated by isolated product mass / (initial styrene mass + initial CB mass)

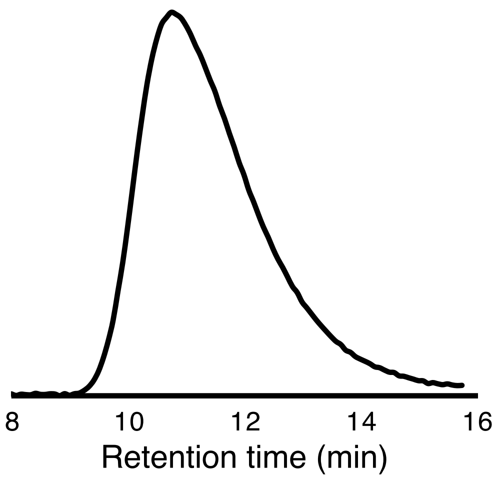

Figure S8. GPC of PS-CB$_{5.1}$.

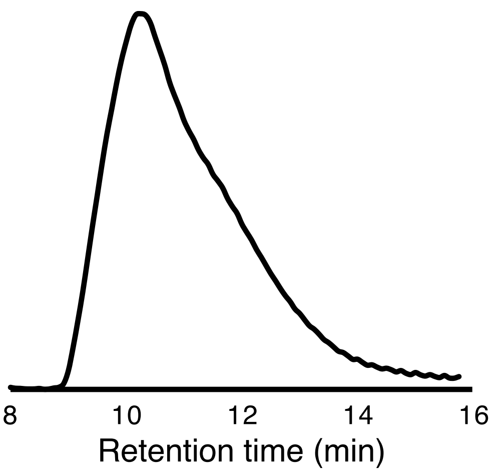

Figure S9. GPC of PS-$CB_{5.5}$.

Procedure for Polystyrene Synthesis via Conventional Heating

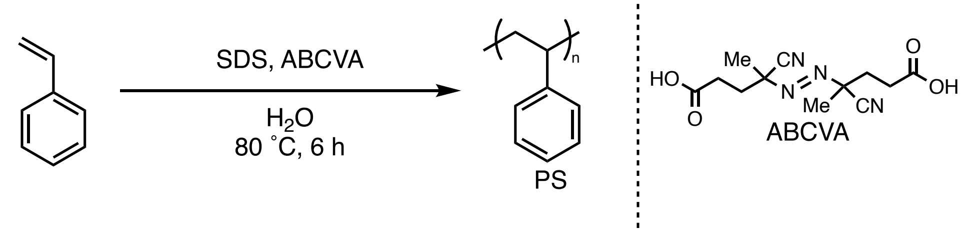

The procedure was slightly modified from the general procedure of polystyrene synthesis via photothermal effect with carbon black. The sample was prepared with the same reagents, except for carbon black. Instead of using 660 nm LED light, the reaction was conducted at 80 ˚C for 6 h. GPC of polystyrene was shown below. (Yield: 88 %, M_n = 105.3 kDa, $Đ$ = 6.12).

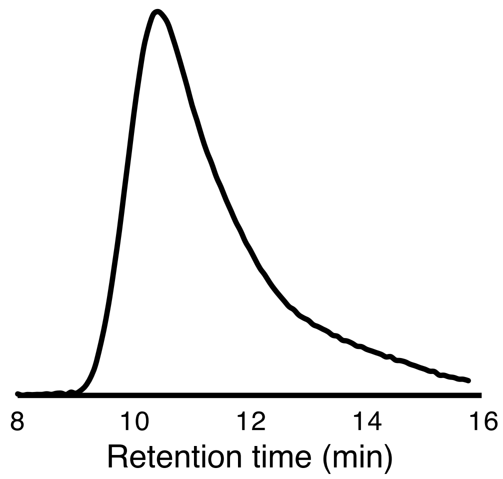

Figure S10. GPC of pure PS.

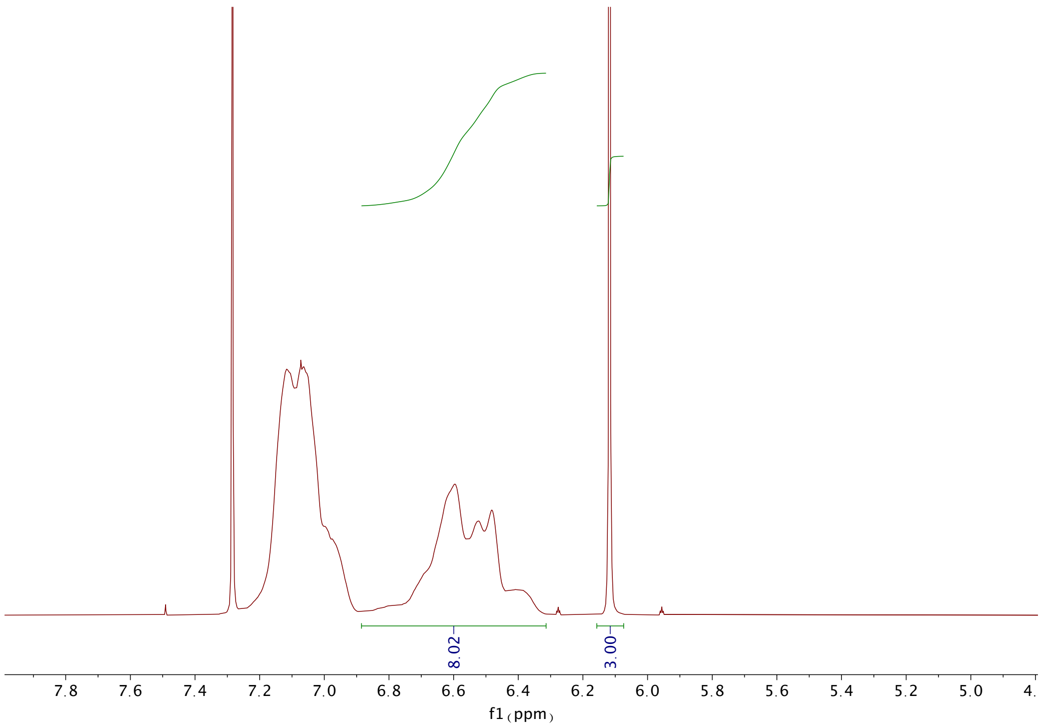

Figure S11. Quantitative ^{1}H NMR of pure PS against 1,3,5-trimethoxybenzene.
*Mole ratio of PS to 1,3,5-trimethoxybenzene is 4 to 1. 1,3,5-trimethoxybenzene integration is 3 for 3 protons (1 for each proton) while PS integration is 8 for 2 protons (4 for each proton). Therefore, this indicates that PS is >99% pure.

Procedure for TEMPO-lated PS Synthesis via Conventional Heating

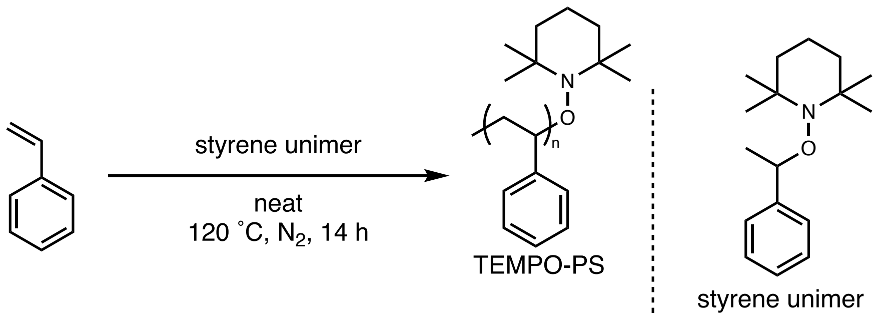

In a 1-dram vial, styrene (10.4 mmol, 1.2 mL, 1.08 g) and styrene unimer (0.02 mmol, 5.2 mg) were added. The mixture was degassed through 3 cycles of freeze-pump-thaw and left under nitrogen. The sample was heated at 120 ˚C and stirred for 2 hours. The sample was removed from the heat source and cooled to room temperature. The crude sample was dissolved in the minimum amount of DCM and precipitated in cold MeOH (50 mL). After vacuum filtration, the sample was dissolved in DCM and precipitated in cold MeOH (50 mL). After vacuum filtration, the sample was dried under high vacuum overnight. GPC of polystyrene was shown below. (Conversion: 53 %, M_n = 17.9 kDa, $Đ$ = 1.23).

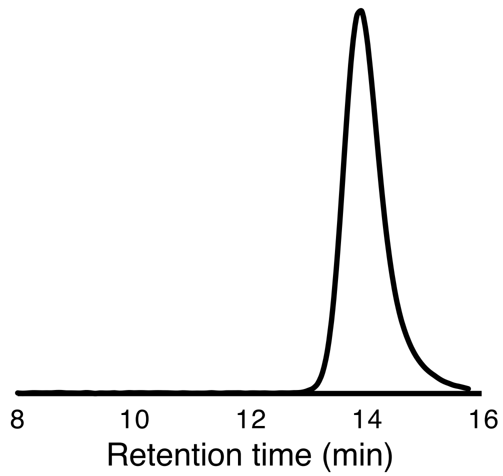

Figure S12. GPC of TEMPO-PS.

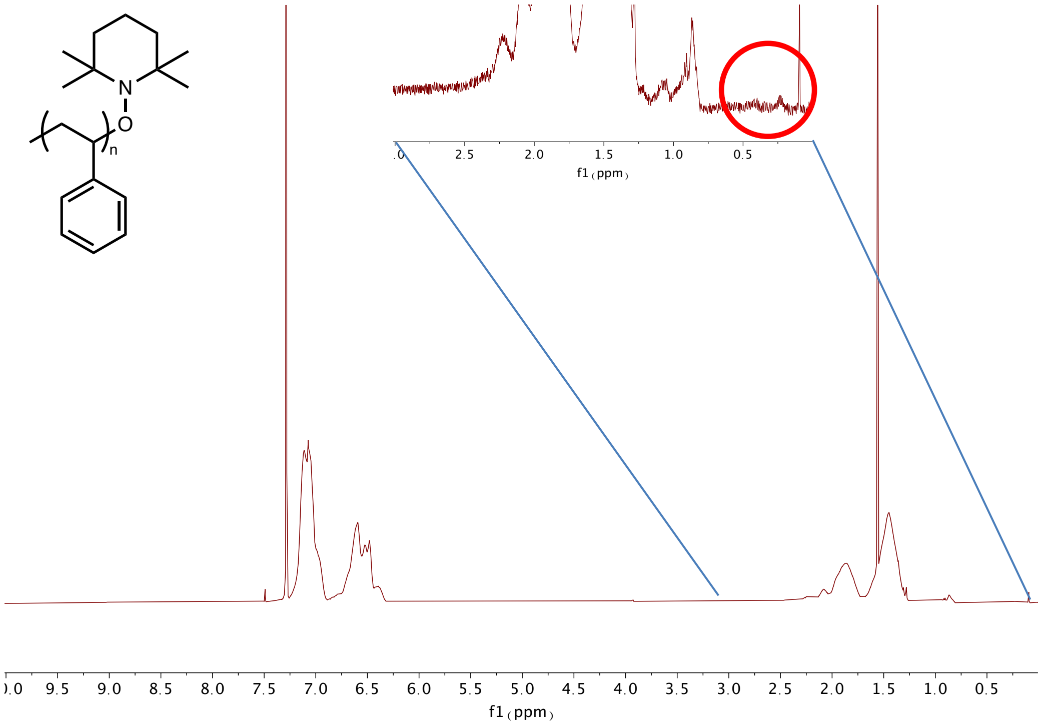

Figure S13. ^{1}H NMR of TEMPO-PS.
*The peaks around 0.2 – 0.5 ppm are the signals from the methyl groups of TEMPO, indicating the presence of TEMPO as a chain-end of polymer.

Procedure for Detempolation of TEMPO-lated PS

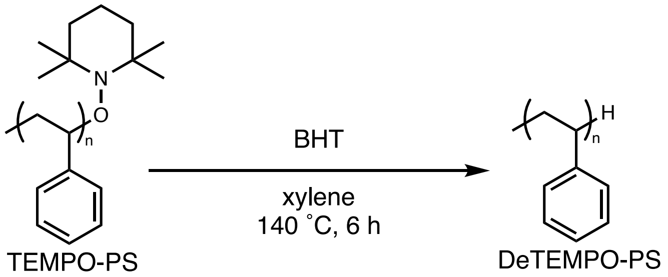

(Synthesis) The TEMPO-lated PS was prepared through the procedure above. (Conversion: 57 %, M_n = 18.4 kDa, $Đ$ = 1.24).

(Detempolation) The procedure was modified from the literature.[3] In a 1-dram vial, TEMPO-PS (200 mg), BHT (0.84 mmol, 158 mg), and xylene (1 mL) were added. The mixture was sparged with N_2 for 20 min. The mixture was heated at 140 ˚C and stirred for 6 h. The sample was removed from the heat source and cooled to room temperature. The crude sample was concentrated in vacuum, dissolved in the minimum amount of DCM, and precipitated in cold MeOH (50 mL). After vacuum filtration, the sample was dissolved in DCM and precipitated in cold MeOH (50 mL). After vacuum filtration, the sample was dried under high vacuum overnight. GPC of polystyrene was shown below. (M_n = 18.0 kDa, $Đ$ = 1.26).

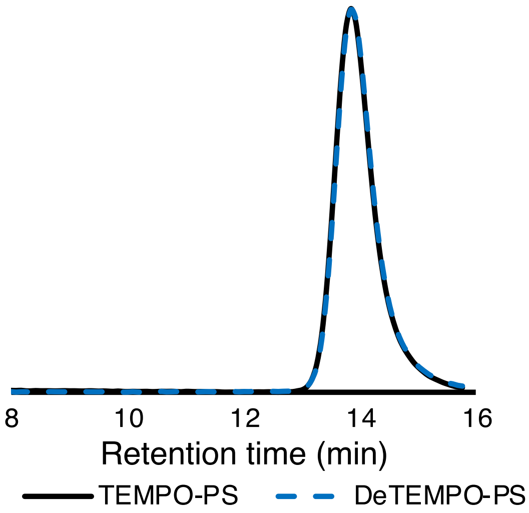

Figure S14. GPC of DeTEMPO-PS.

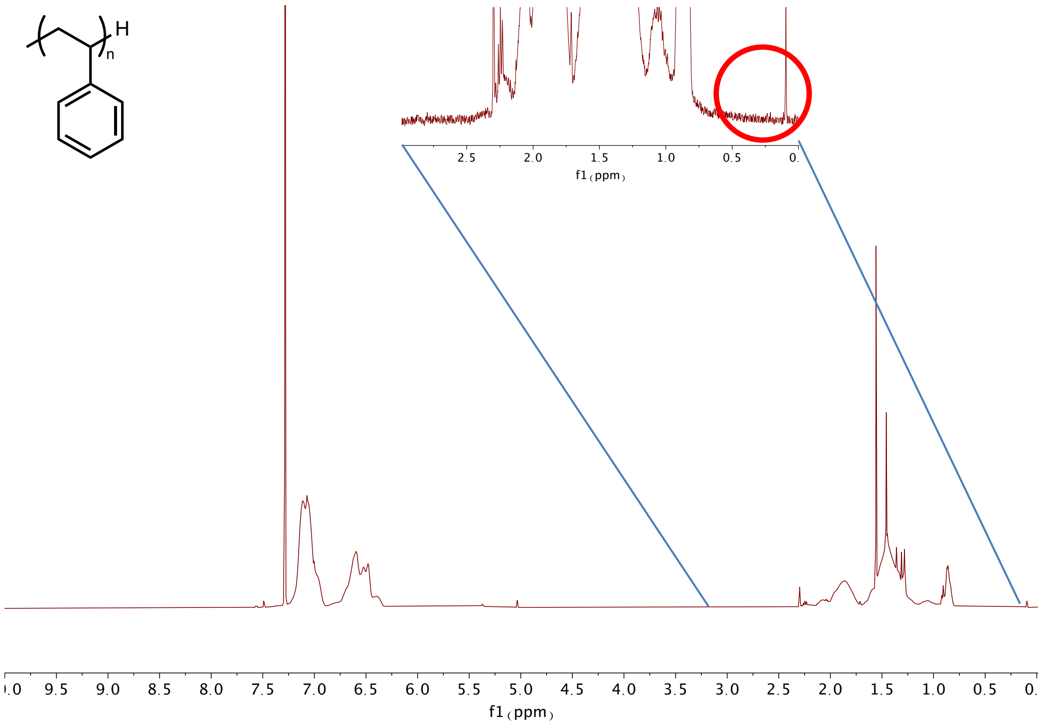

Figure S15. ^{1}H NMR of DeTEMPO-PS.
*The peaks around 0.2 – 0.5 ppm disappeared, indicating the complete removal of TEMPO from the chain-end of polymer.

Procedure for Styrene Acrylonitrile Copolymer Synthesis via Photothermal Conversion

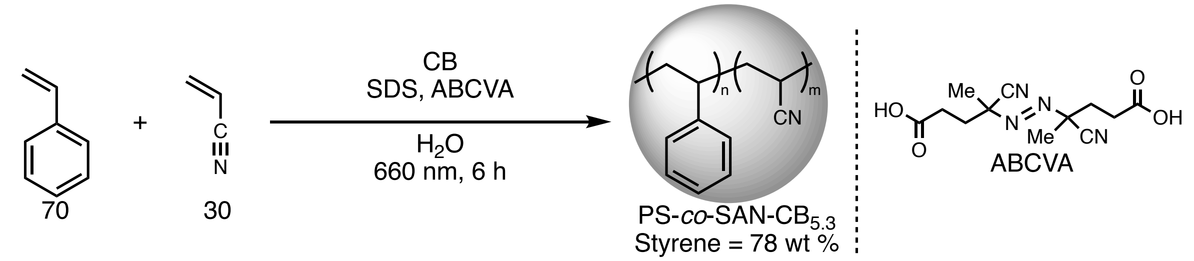

The procedure was slightly modified from the general procedure of polystyrene synthesis via photothermal conversion with carbon black. Carbon black (51.8 mg) was used. For the sample preparation, styrene (7.0 mmol, 805 µL, 724 mg, 70 wt %) and acrylonitrile (5.9 mmol, 385 µL, 311 mg, 30 wt %) were added to the aqueous solution. GPC and ^{1}H NMR spectra of copolymer were shown below. (Yield: 90 %, M_n = 245.2 kDa, $Đ$ = 6.86, Styrene = 78 wt %, Acrylonitrile = 22 wt %, CB_{incorp} = 5.3 wt %).

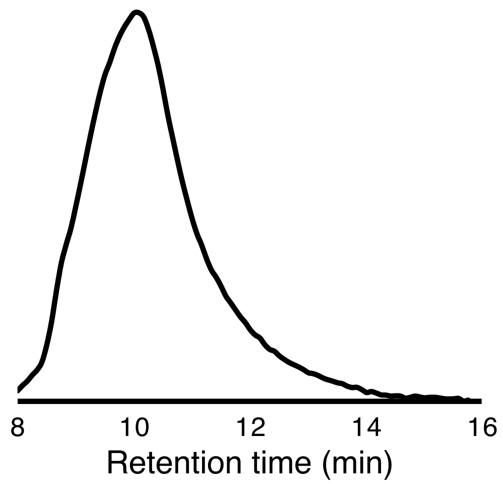

Figure S16. GPC of PS-*co*-PAN-$CB_{5.3}$.

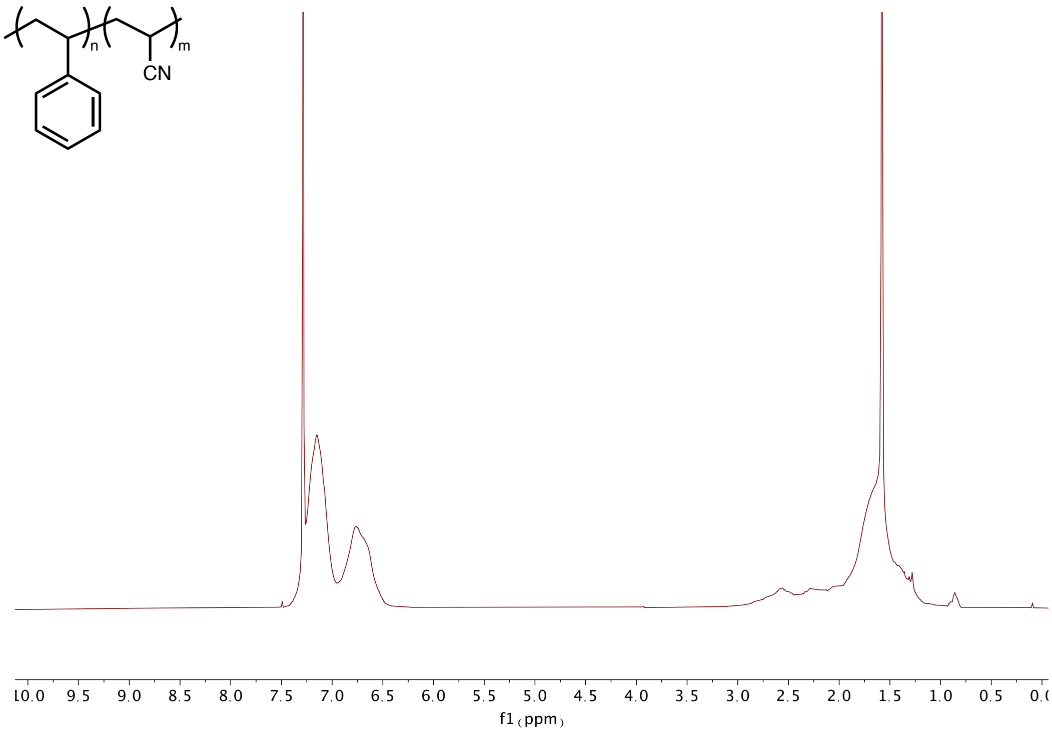

Figure S17. ^{1}H NMR of PS-*co*-PAN-CB$_{5.3}$.

Procedure for Styrene Methyl Acrylate Copolymer Synthesis via Photothermal Conversion

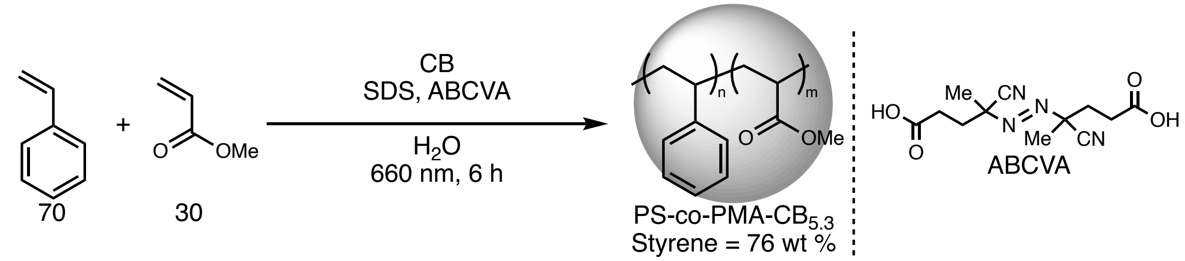

The procedure was slightly modified from the general procedure of polystyrene synthesis via photothermal conversion with carbon black. Carbon black (51.8 mg) was used. For the sample preparation, styrene (7.0 mmol, 805 μL, 724 mg, 70 wt %) and methyl acrylate (3.6 mmol, 325 μL, 30 wt %) were added to the aqueous solution. GPC and ^{1}H NMR spectra of copolymer were shown below. (Yield: 90 %, M_n = 96.7 kDa, $Đ$ = 5.29, Styrene = 76 wt %, MA = 24 wt %, CB_{incorp} = 5.3 wt %).

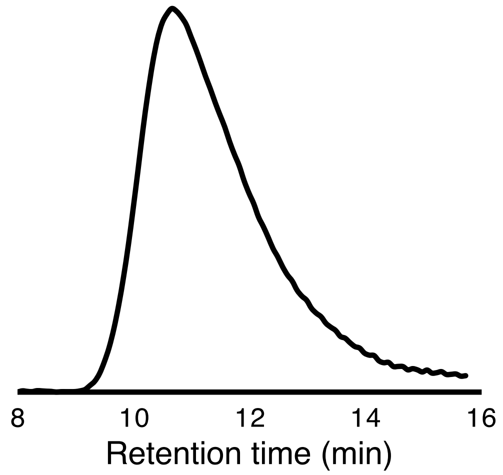

Figure S18. GPC of PS-*co*-PMA-$CB_{5.3}$.

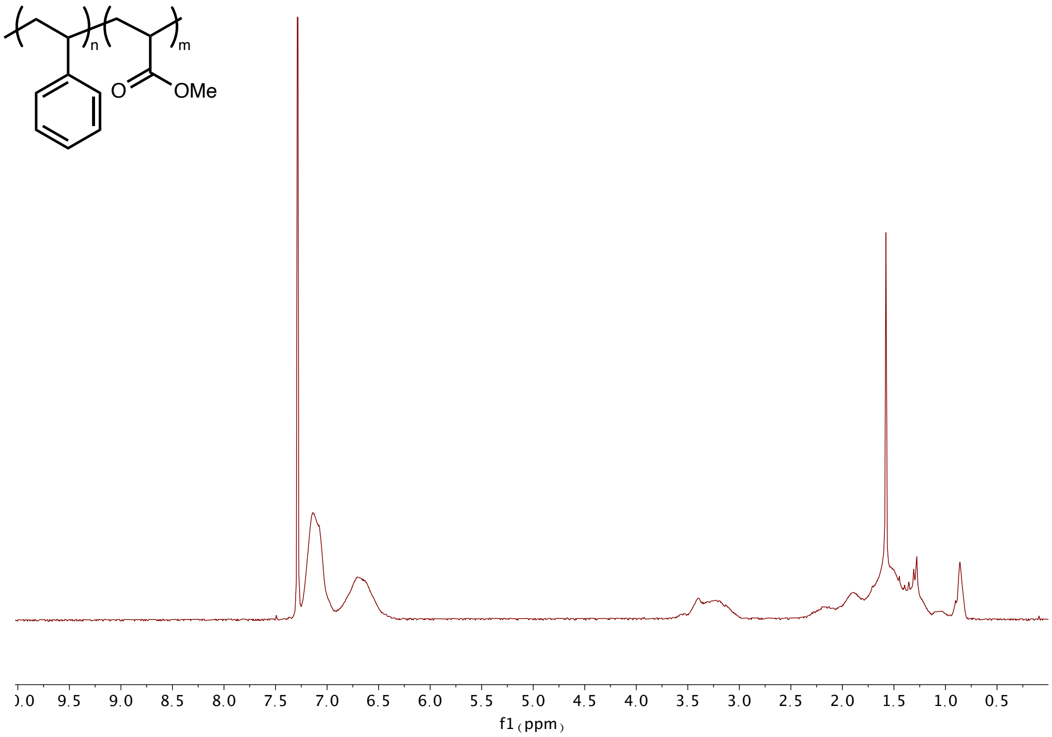

Figure S19. ^{1}H NMR of PS-*co*-PMA-CB$_{5.3}$.

Procedure for Styrene Isoprene Copolymer Synthesis via Photothermal Conversion

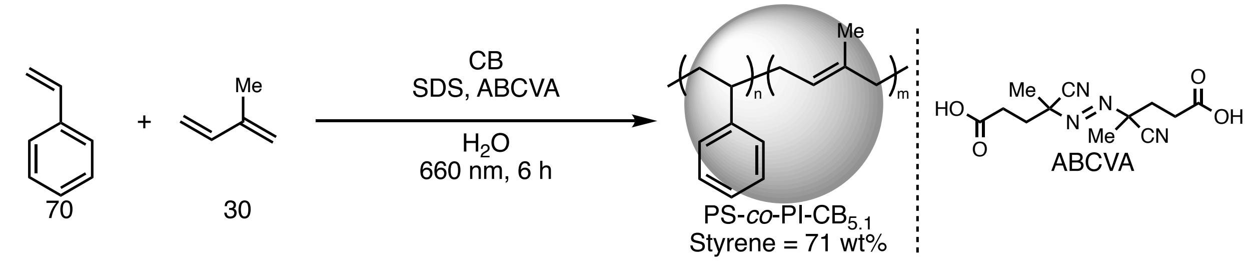

(Styrene 70 wt % in copolymer) The procedure was slightly modified from the general procedure of polystyrene synthesis via photothermal conversion with carbon black. Carbon black (51.8 mg) was used. For the sample preparation, styrene (7.0 mmol, 805 μL, 724 mg, 70 wt %) and isoprene (4.6 mmol, 460 μL, 313 mg, 30 wt %) were added to the aqueous solution. GPC and ^{1}H NMR spectra of copolymer were shown below. T_d of copolymer were obtained from TGA. The data are listed below and spectrum was shown below. (Yield: 90 %, M_n = 53.2 kDa, $Đ$ = 12.8, Styrene = 71 wt %, Isoprene = 29 wt %, CB_{incorp} = 5.1 wt %, T_d = 365.6 ˚C).

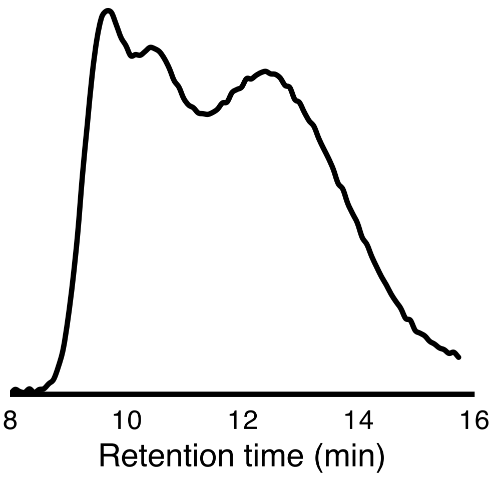

Figure S20. GPC of PS-*co*-PI-$CB_{5.1}$.

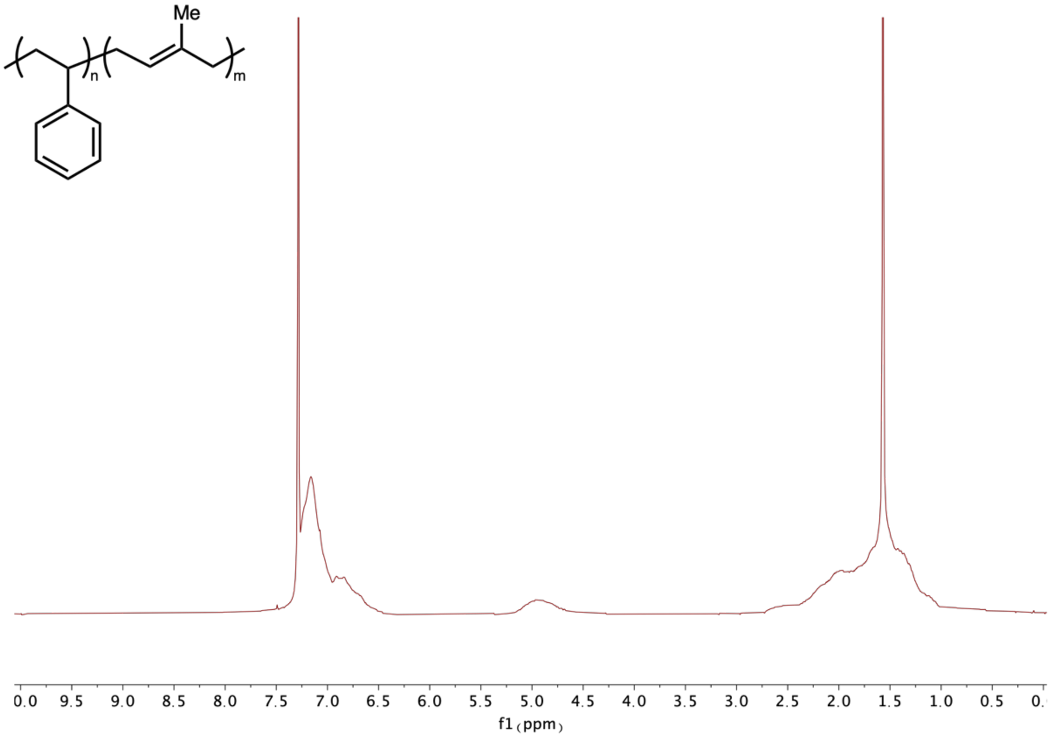

Figure S21. ^{1}H NMR of PS-*co*-PI-CB$_{5.1}$.

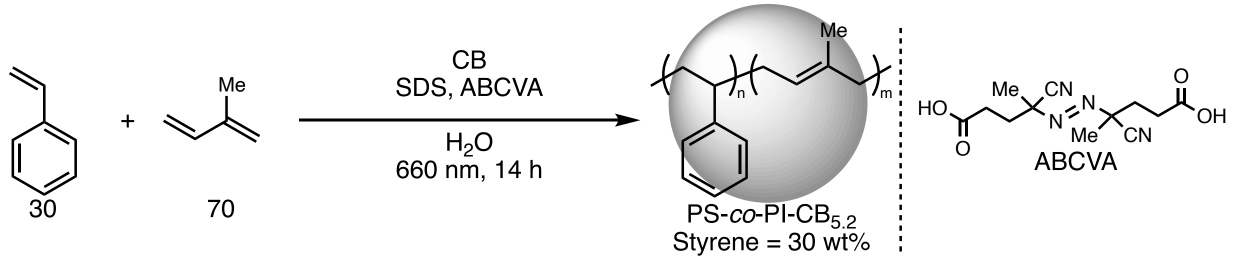

(Styrene 30 wt % in copolymer) The procedure was slightly modified from the general procedure of polystyrene synthesis via photothermal conversion with carbon black. Carbon black (51.8 mg) was used. For the sample preparation, styrene (2.9 mmol, 340 µL, 306 mg, 30 wt %) and isoprene (10.6 mmol, 1.06 mL, 721 mg 70 wt %) were added to the aqueous solution. The reaction was irradiated for 14 h, instead of 6 h. Lastly, cold acidified MeOH (MeOH (130 mL) + deionized H_2O (20 mL) + HCl (1 mL)) was used for polymer precipitation. The resulting polymer was not soluble in organic solvent, so GPC and ^{1}H NMR spectra of styrene isoprene copolymer were not taken. It was assumed that the sample's composition was styrene (30 wt %) and isoprene (70 wt %) (Yield: 91 %, CB_{incorp} = 5.2 wt %).

Procedure for Hydrogenation of Styrene Isoprene Copolymer

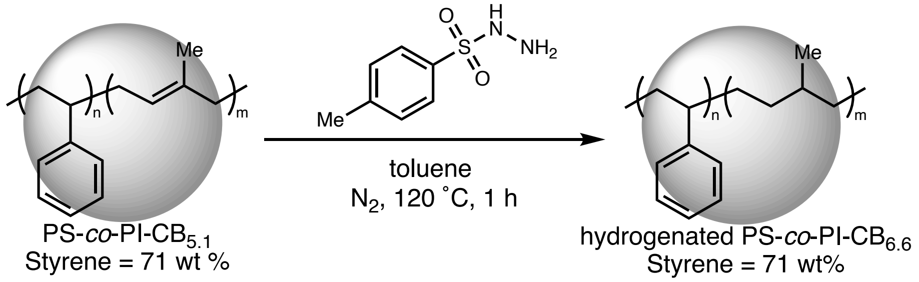

The procedure was modified from the literature.[4] In a flame-dried round bottom flask, PS-*co*-PI-$CB_{5.1}$ (350 mg, carbon black = 5.1 wt %, styrene = 71 wt %) and p-toluenesulfonyl hydrazide (4.38 mmol, 816 mg, 3.0 equiv. based on isoprene repeating units) were added. After 3 cycles of evacuation and backfilling with N_2, the system was placed under N_2. Anhydrous toluene (14 mL) was added. The reaction was stirred at 120 ˚C overnight. The sample was cooled to room temp. The polymer was precipitated in cold MeOH (100 mL) and collected through vacuum filtration. The polymer was dissolved in a minimum amount of DCM and precipitated in cold MeOH (100 mL). After collecting the polymer through vacuum filtration, the polymer was dried under high vacuum overnight. GPC and ^{1}H NMR spectra of hydrogenated copolymer were shown below. T_d of copolymer were obtained from TGA. The data are listed below and spectrum was shown below. (Hydrogenation: >99 %, Yield: 77 %, M_n = 59.1 kDa, $Đ$ = 7.29, CB_{incorp} = 6.6 wt %, T_d = 395.2 ˚C).

*Note CB_{incorp} increased due to losing some polymer (23 %) while quantitatively collecting CB.

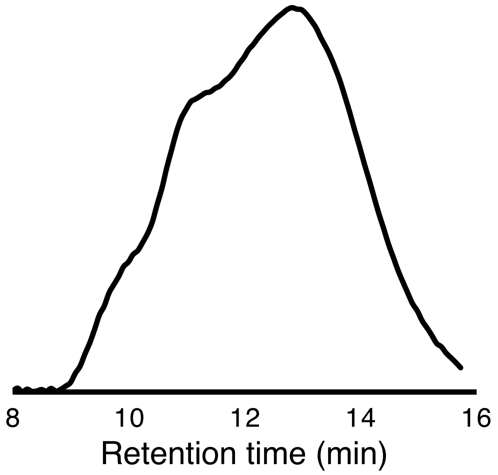

Figure S22. GPC of hydrogenated PS-*co*-PI-$CB_{6.6}$.

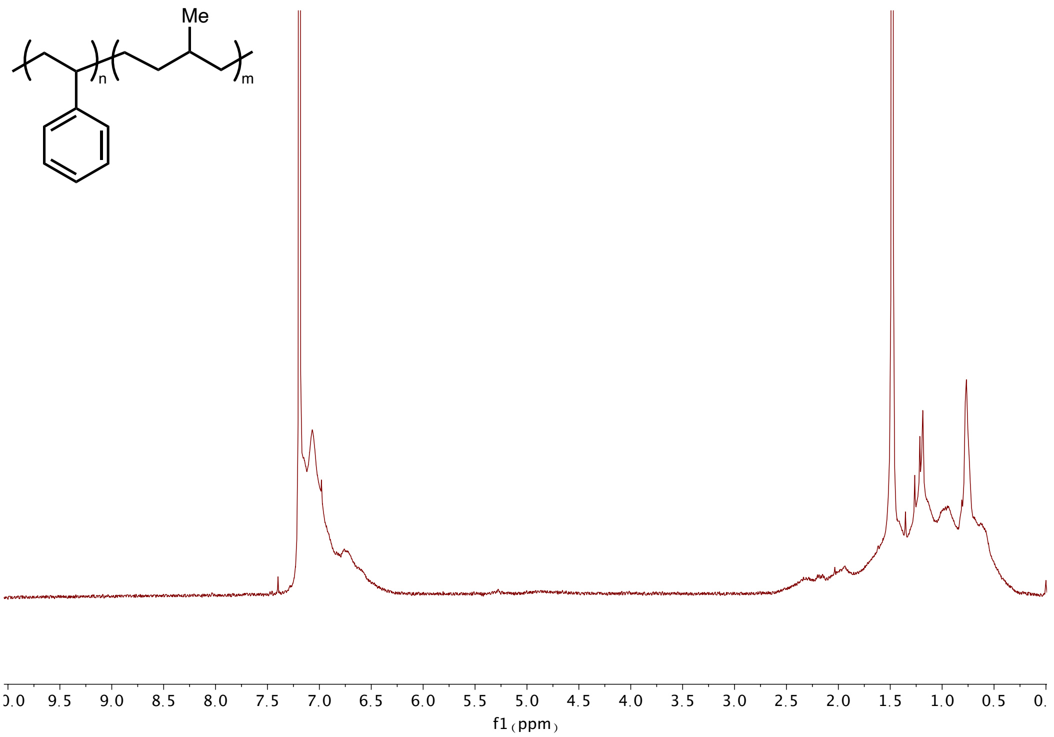

Figure S23. ^{1}H NMR of hydrogenated PS-*co*-PI-CB$_{6.6}$.

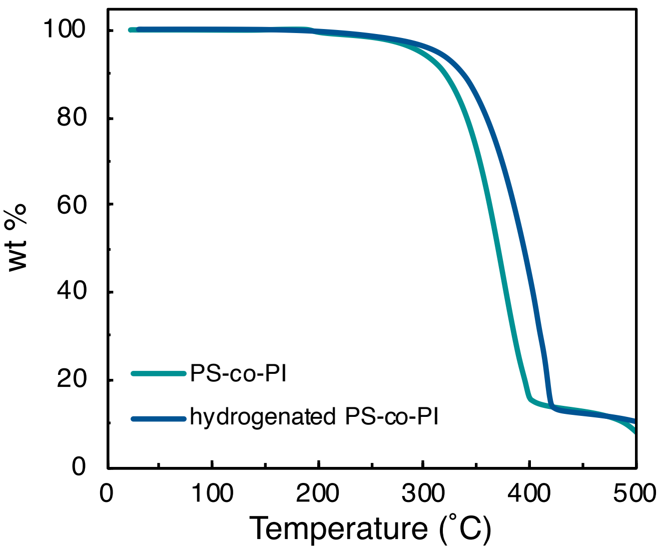

Figure S24. TGA of PS-*co*-PI (styrene = 71 wt %) before and after hydrogenation

Procedure for High Impact Polystyrene (HIPS) via Photothermal Conversion

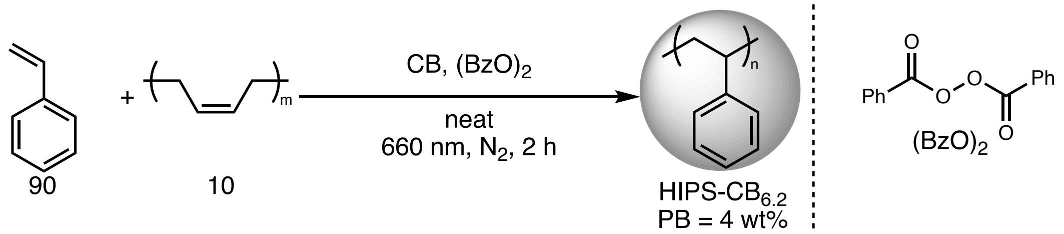

In a 1-dram vial, styrene (9.0 mmol, 1.04 mL, 936 mg, 90 wt %), polybutadiene (99 mg, 10 wt %), $(BzO)_2$ (4.5 μmol, 1.1 mg), and carbon black (31.1 mg) were added. The mixture was stirred until polybutadiene was dissolved. The mixture was degassed through 3 cycles of freeze-pump-thaw and left it under nitrogen. The sample was irradiated with 660 nm LED light and stirred for 2 hours. The sample was removed from the light source and cooled to room temperature. The crude sample was dissolved in the minimum amount of DCM and precipitated in cold MeOH (50 mL). After vacuum filtration, the sample was dissolved in DCM and precipitated in cold MeOH (50 mL). After vacuum filtration, the sample was dried under high vacuum overnight.

Hexane (10 mL) was added to the dried sample and stirred at 70 ˚C for an hour to remove any unreacted polybutadiene. After vacuum filtration, the sample was dried under high vacuum overnight. GPC, ^{1}H NMR, and diffusion NMR spectra of polystyrene were shown below. (Yield: 47 %, M_n = 35.4 kDa, $Đ$ = 4.62, Styrene = 96 wt %, Polybutadiene = 4 wt %, CB_{incorp} = 6.2 wt %).

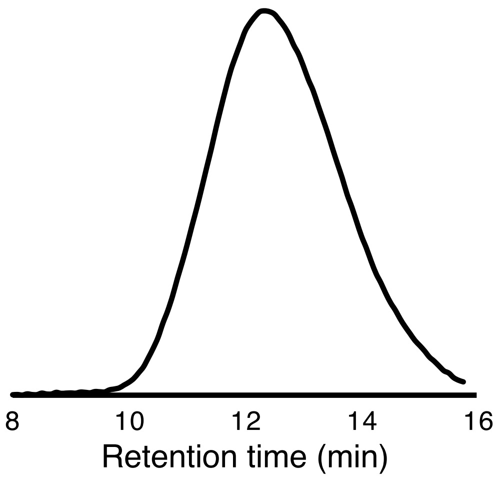

Figure S25. GPC of HIPS-$CB_{6.2}$.

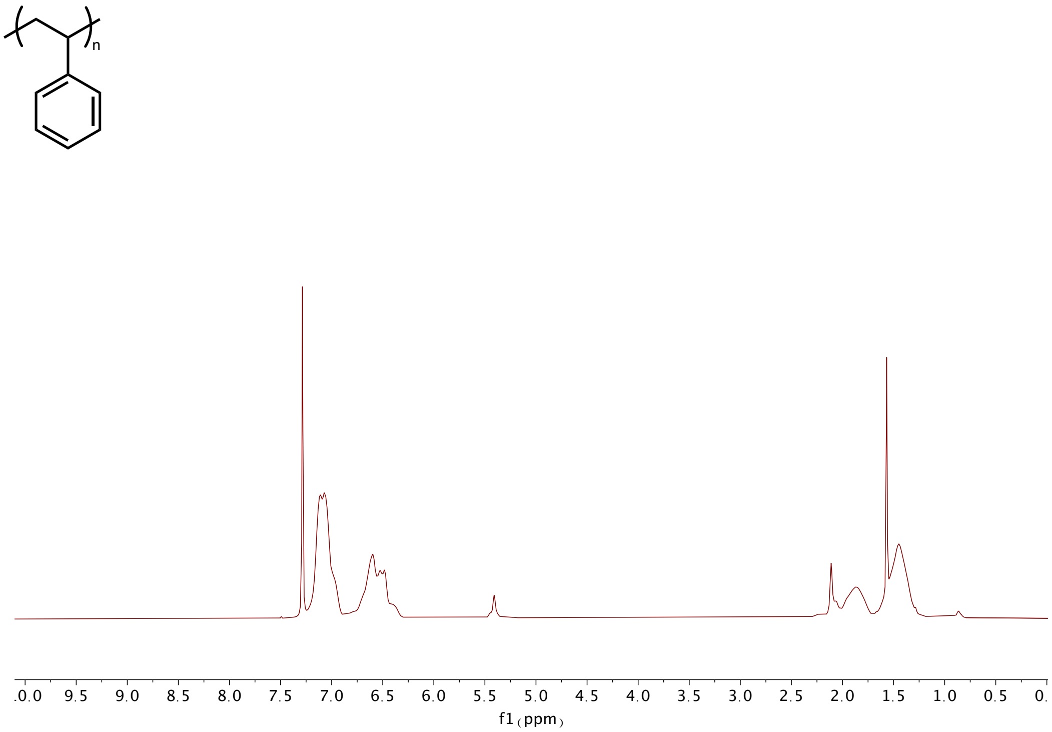

Figure S26. ^{1}H NMR of HIPS-$CB_{6.2}$.

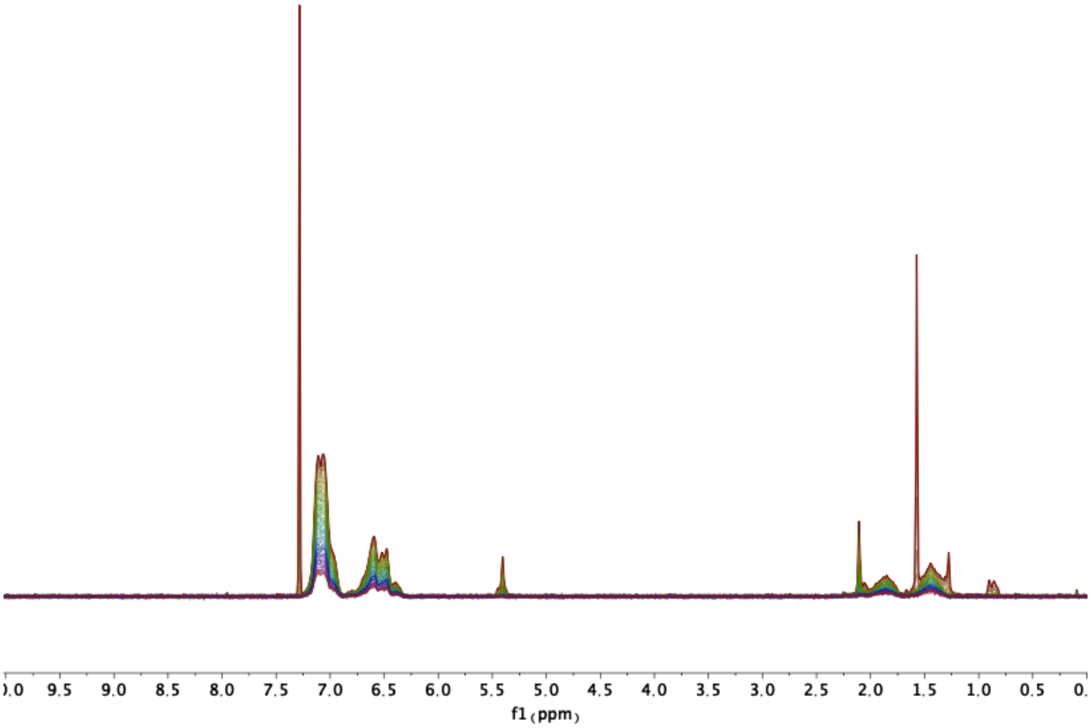

Figure S27. Diffusion NMR of the mixture of pure PS and polybutadiene.

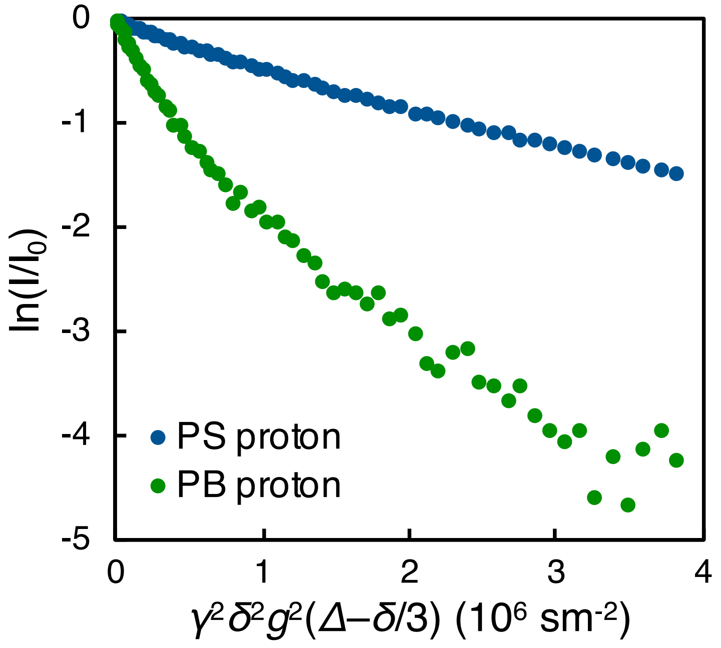

Figure S28. Stejskal–Tanner plot of pure PS and polybutadiene.
PS proton integration is measured at 6.28–6.87 ppm while PB proton is measured at 5.34–5.47.

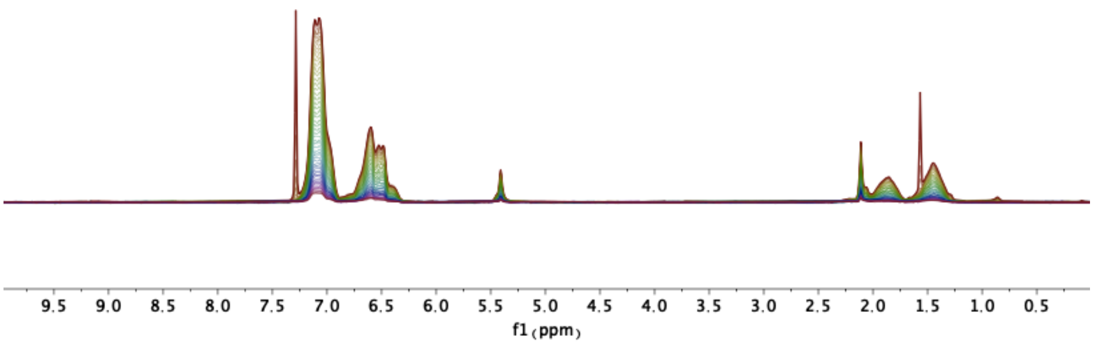

Figure S29. Diffusion NMR of HIPS-$CB_{6.2}$.

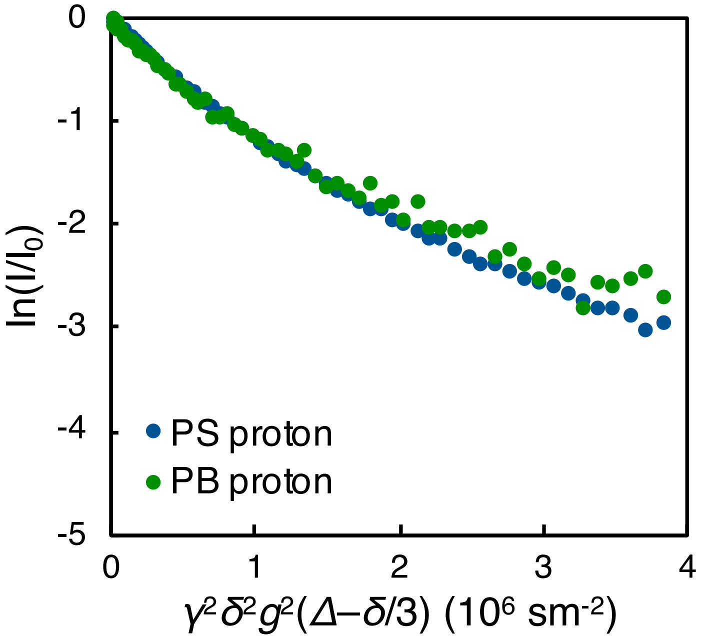

Figure S30. Stejskal–Tanner plot of HIPS-$CB_{6.2}$.
PS proton integration is measured at 6.28–6.87 ppm while PB proton is measured at 5.34–5.47.

Procedure for Photothermal Depolymerization under Air

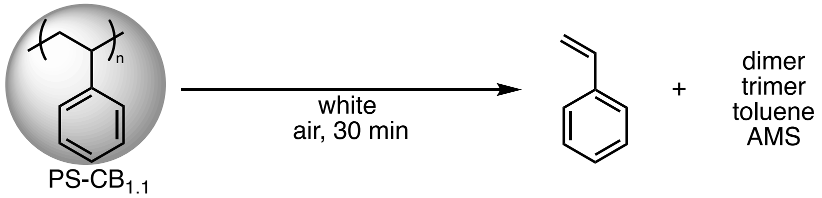

In a 1-dram vial, PS-$CB_{1.1}$ (50 mg, carbon black = 1.1 wt %) was added and sealed with a cap equipped with a Teflon septum. The reaction vial was placed 0.2 mm above 6000 K white LED light (100 W) and irradiated for 30 min. The vial was removed from the light source and cooled to room temperature. The vial was placed in the dry ice acetone bath for 3 min and defrosted to room temperature. $CDCl_3$ (0.5 mL) and a 1,3,5-trimethoxybenzene stock solution (0.5 mL, 0.24 M in $CDCl_3$) were added to the reaction vial. Aliquots were taken for GPC and ^{1}H NMR analysis and shown below.

Table S3. Results of photothermal depolymerization under air.

Entry	M_n (kg/mol)	Đ	styrene (%)	dimer (%)	trimer (%)	toluene (%)	AMS (%)^a	leftover PS (%)	mass recov. (%)
1	5.2	1.66	41.7	7.0	0	3.0	3.0	10.6	65.3

All % with regard to styrene repeating units
^{a}Alpha-methylstyrene (AMS)

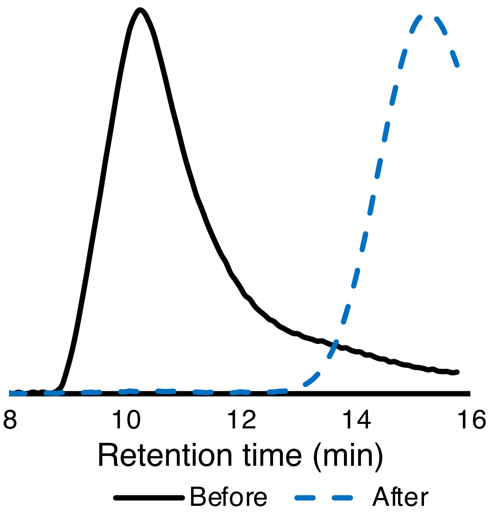

Figure S31. GPC of photothermal depolymerization under air.

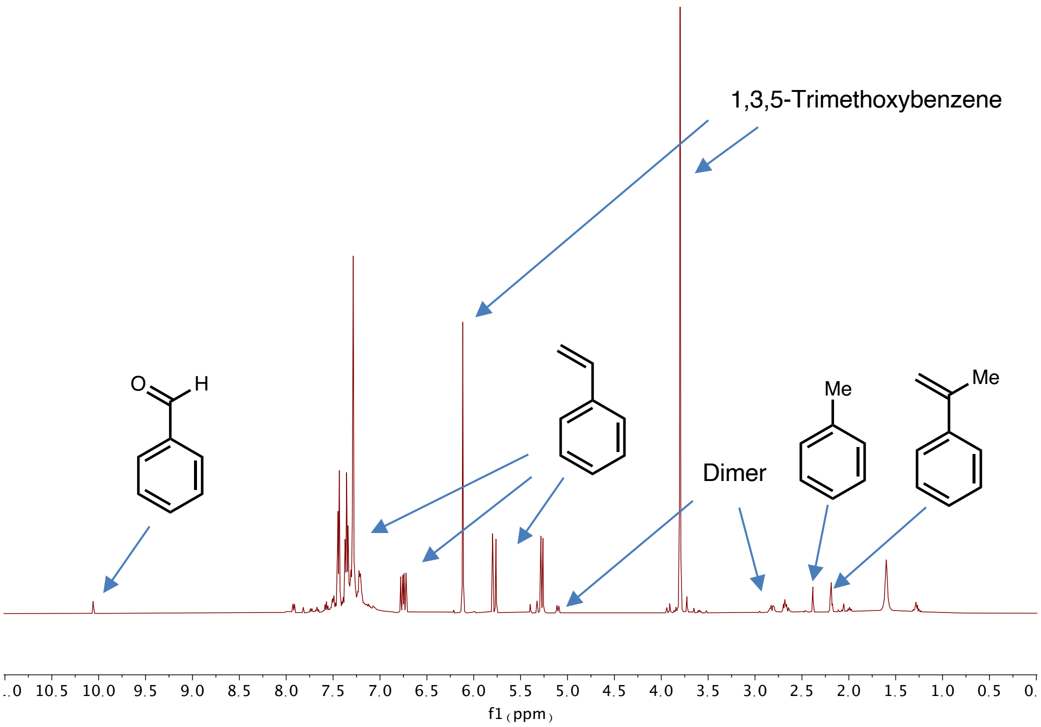

Figure S32. ^{1}H NMR of photothermal depolymerization under air.

Procedure for Photothermal Depolymerization under Nitrogen

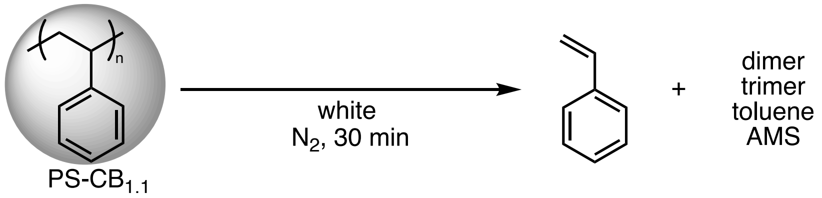

In a 1-dram vial, $PS\text{-}CB_{1.1}$ (50 mg, carbon black = 1.1 wt %) was added and sealed with a cap equipped with a Teflon septum. Oxygen was removed through 3 cycles of freeze-pump-thaw and left it under nitrogen. The reaction vial was placed 0.2 mm above 6000 K white LED light (100 W) and irradiated for 30 min. The vial was removed from the light source and cooled to room temperature. The vial was placed in the dry ice acetone bath for 3 min and defrosted to room temperature. $CDCl_3$ (0.5 mL) and a 1,3,5-trimethoxybenzene stock solution (0.5 mL, 0.24 M in $CDCl_3$) were added to the reaction vial. Aliquots were taken for GPC and ^{1}H NMR analysis and shown below.

Table S4. Results of $PS\text{-}CB_{1.1}$ after photothermal depolymerization under nitrogen.

Entry	M_n (kg/mol)	$Đ$	styrene (%)	dimer (%)	trimer (%)	toluene (%)	AMS (%)[a]	leftover PS (%)	mass recov. (%)
1	15.9	3.65	40.4	9.0	6.0	0.8	1.6	30.3	88.1

All % with regard to styrene repeating units
[a]Alpha-methylstyrene (AMS)

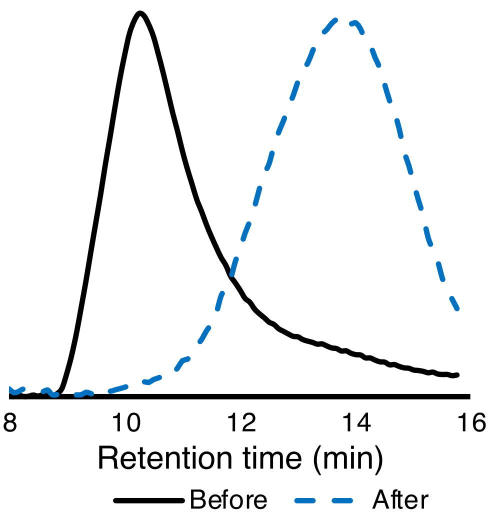

Figure S33. GPC of $PS\text{-}CB_{1.1}$ after photothermal depolymerization under nitrogen.

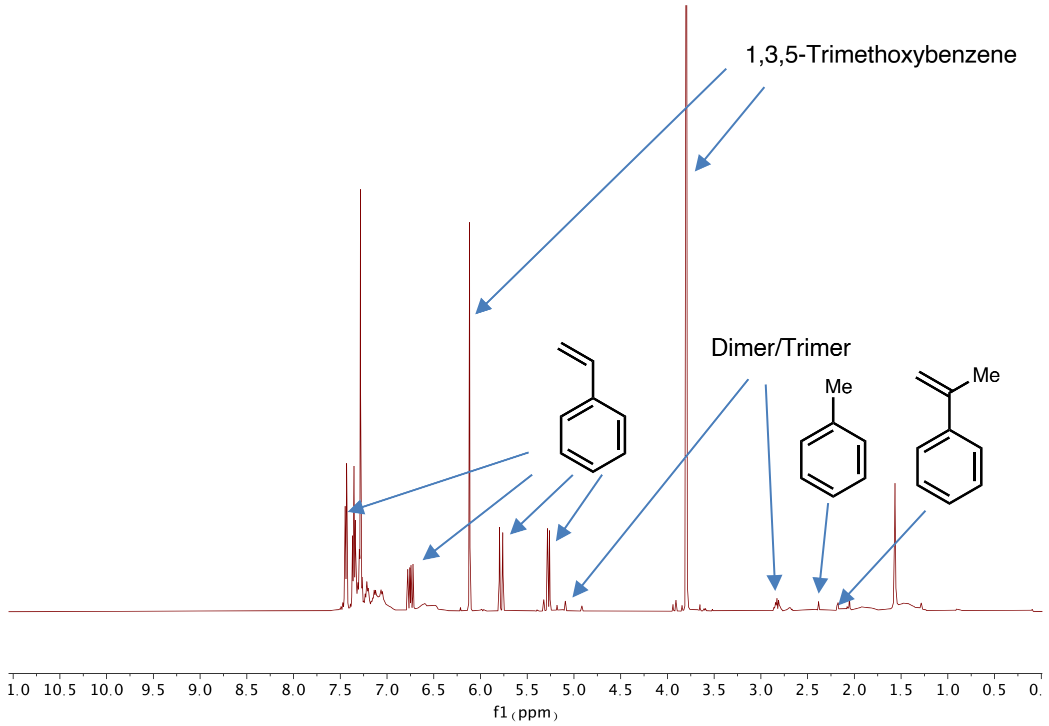

Figure S34. ^{1}H NMR of PS-$CB_{1.1}$ after photothermal depolymerization under nitrogen.

Procedure for Photothermal Depolymerization under Dynamic Vacuum

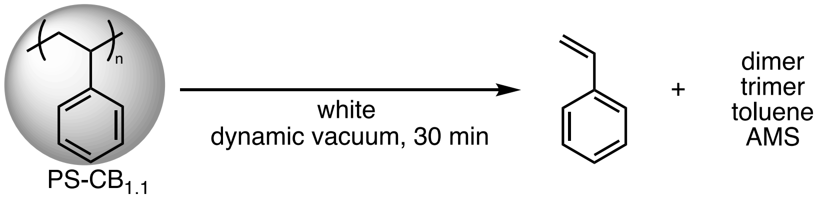

In a 1-dram vial, PS-$CB_{1.1}$ (50 mg, carbon black = 1.1 wt %) was added and PTFE tape was wrapped around the top of the vial. The vial was equipped with a short distillation path and a receiving flask. The setup was placed 0.2 mm above 6000 K white LED light (100 W). Ice cold water was used for the condenser, and dry ice acetone bath was used for the receiving flask. The setup was evacuated and backfilled with nitrogen three times and left it under dynamic vacuum. The reaction was irradiated for 30 min. After the reaction, the light was turned off, and the setup was filled with nitrogen. The setup was left until reaching at room temperature. In the reaction vial and receiving flask, $CDCl_3$ (1 mL) and a 1,3,5-trimethoxybenzene stock solution (0.5 mL, 0.24 M in $CDCl_3$) were added. The condenser was rinsed with $CDCl_3$ (2 mL), and a 1,3,5-trimethoxybenzene stock solution (0.5 mL, 0.24 M in $CDCl_3$) was added to the collected sample. Aliquots of the reaction vial, receiving flask, and condenser were taken for ^{1}H NMR (and GC-MS, if necessary) analysis and an aliquot of the reaction vial was taken for GPC analysis, and shown below.

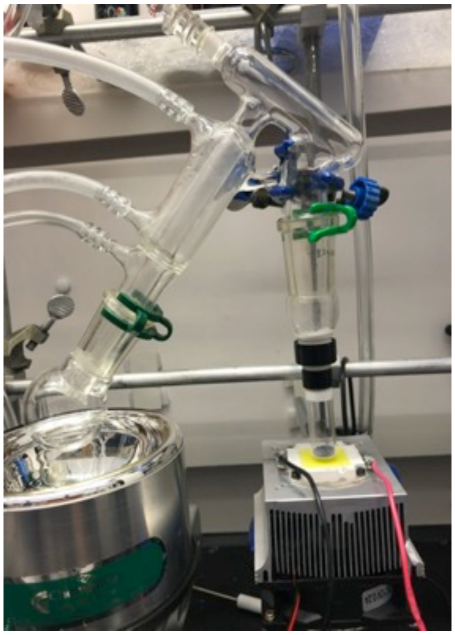

Figure S35. Reaction setup of photothermal depolymerization under vacuum.

Table S5. Results of PS-CB$_{1.1}$ after photothermal depolymerization under vacuum.

Entry	M_n (kg/mol)	$Đ$	styrene (%)	dimer (%)	trimer (%)	toluene (%)	AMS (%)[a]	leftover PS (%)	mass recov. (%)
1	15.4	2.98	32.9	5.0	11.4	0.5	0.3	38.9	89.0

All % with regard to styrene repeating units
[a]Alpha-methylstyrene (AMS)

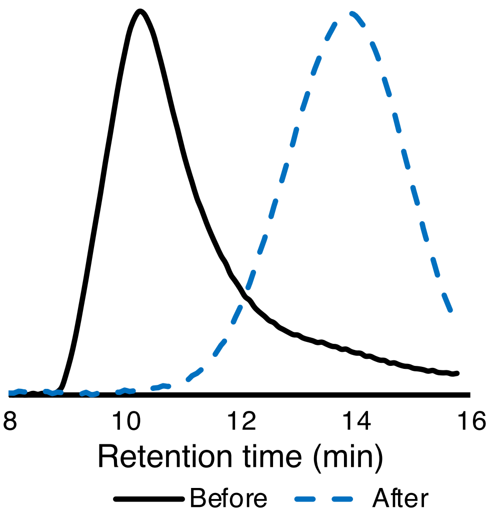

Figure S36. GPC of PS-CB$_{1.1}$ after photothermal depolymerization under vacuum.

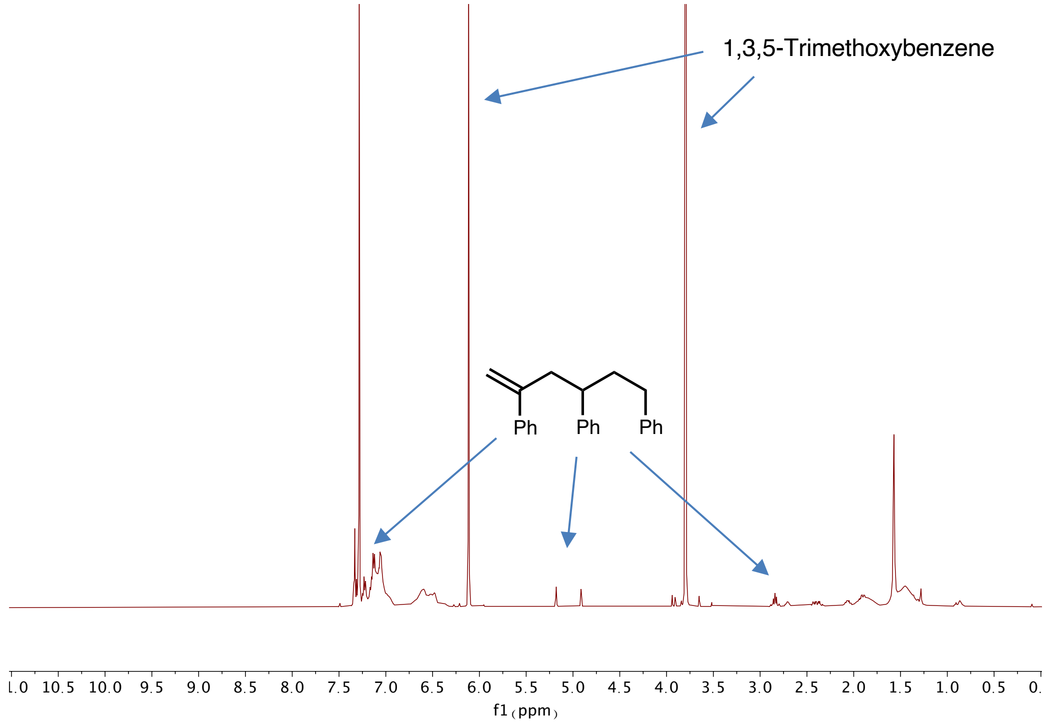

Figure S37. ^{1}H NMR of PS-CB$_{1.1}$ after photothermal depolymerization under vacuum (reaction vial).

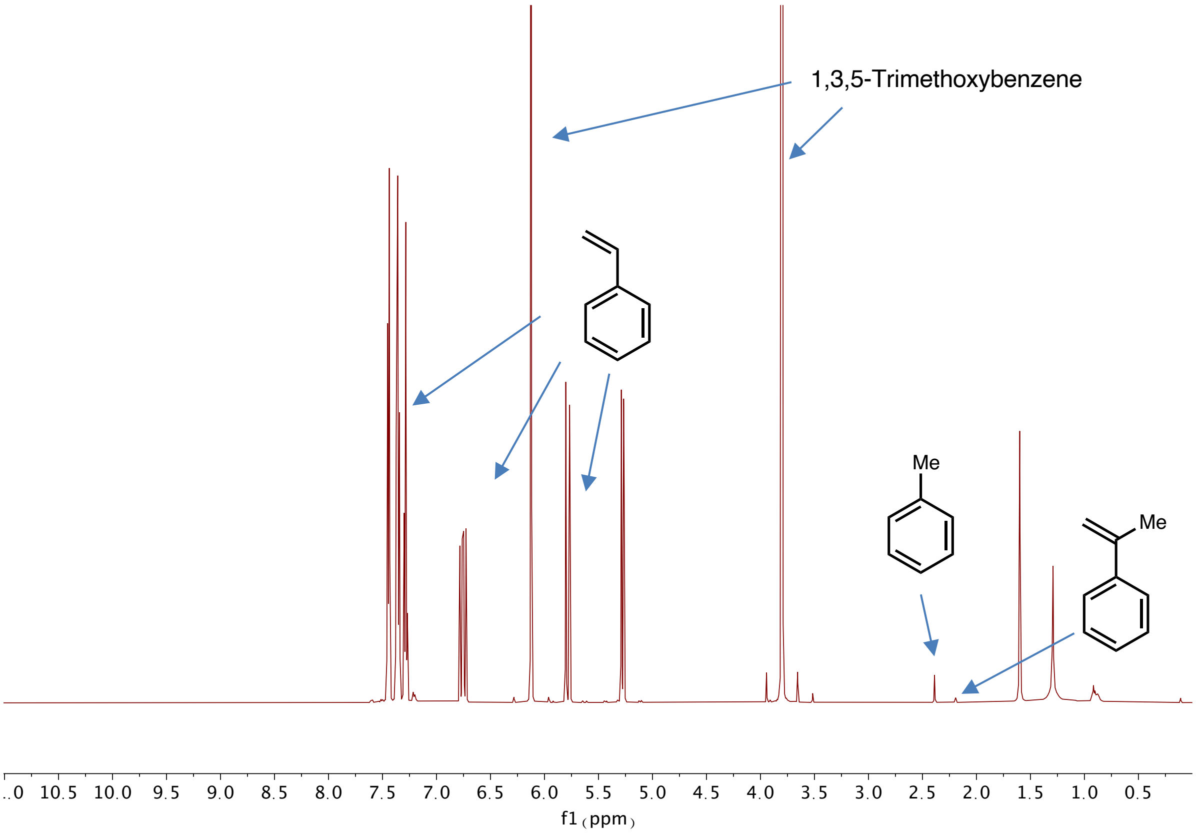

Figure S38. ^{1}H NMR of PS-CB$_{1.1}$ after photothermal depolymerization under vacuum (receiving flask).

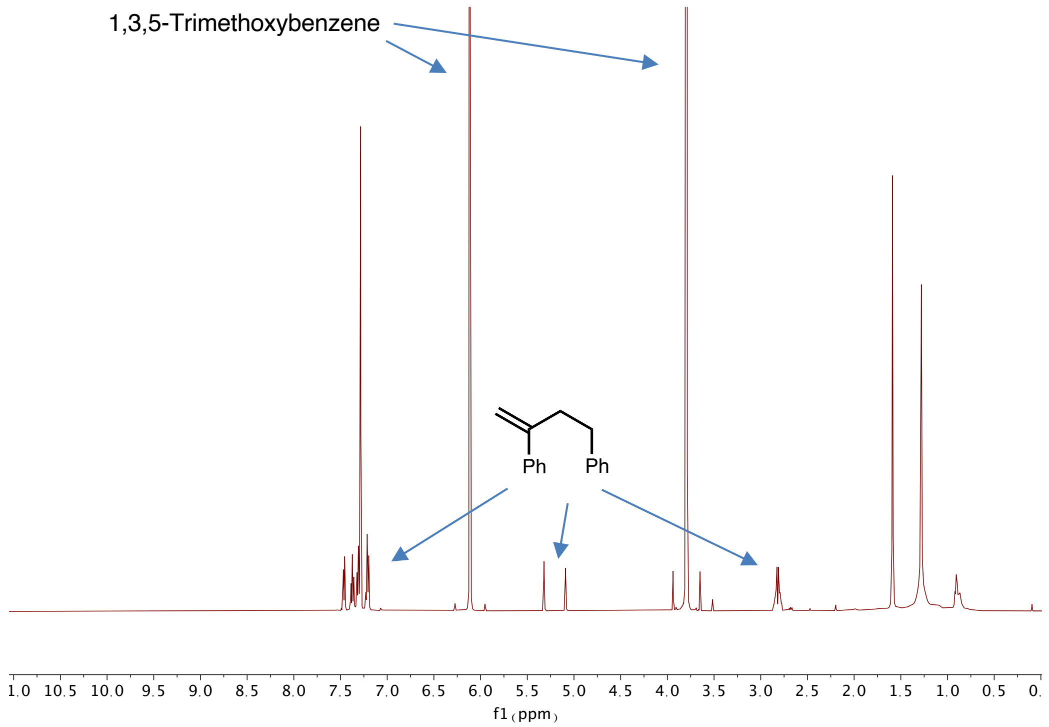

Figure S39. ^{1}H NMR of PS-CB$_{1.1}$ after photothermal depolymerization under vacuum (condenser).

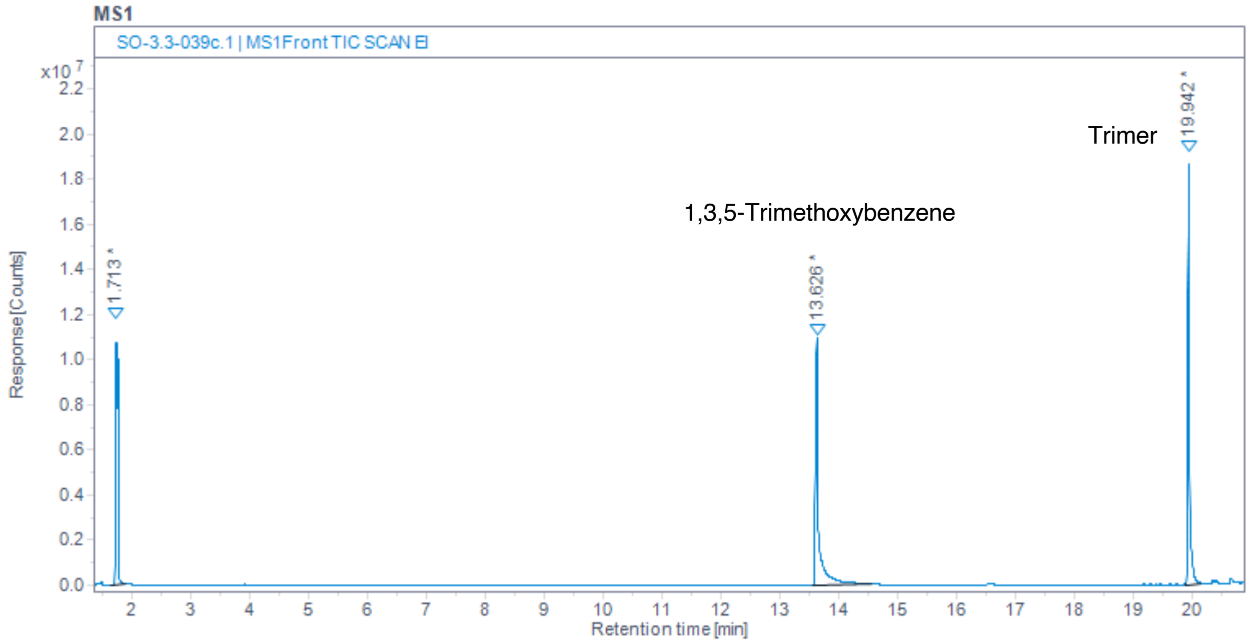

Figure S40. GC-MS of PS-CB$_{1.1}$ after photothermal depolymerization under vacuum (reaction vial).

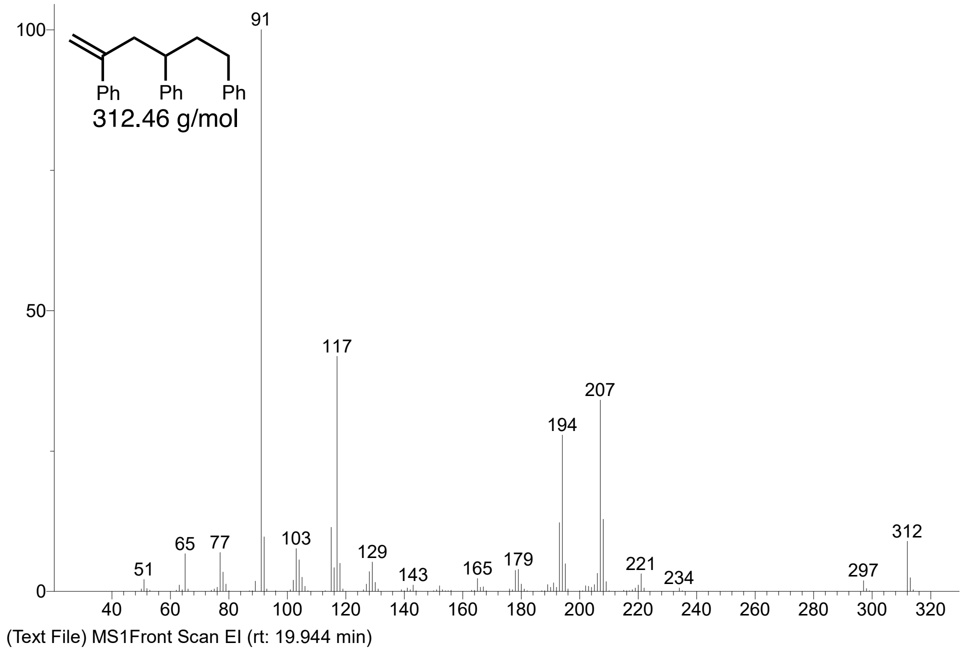

Figure S41. Mass spectrum of Fig. S35 at 19.94 min.

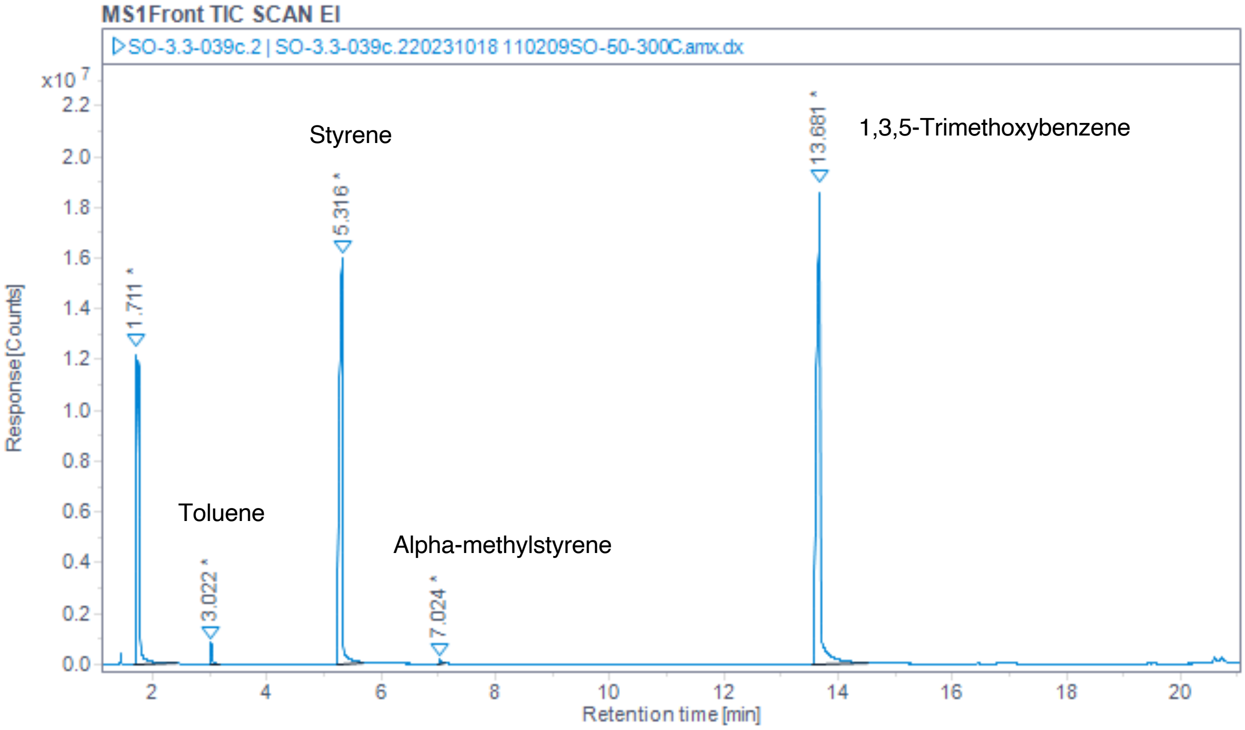

Figure S42. GC-MS of PS-CB$_{1.1}$ after photothermal depolymerization under vacuum (receiving flask).

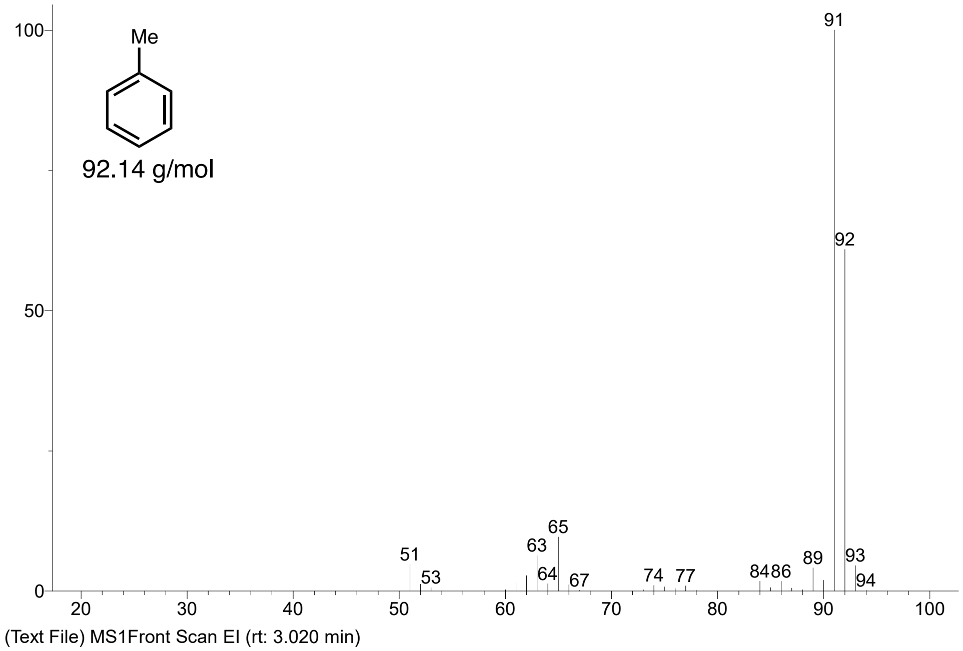

Figure S43. Mass spectrum of Fig. S37 at 3.02 min.

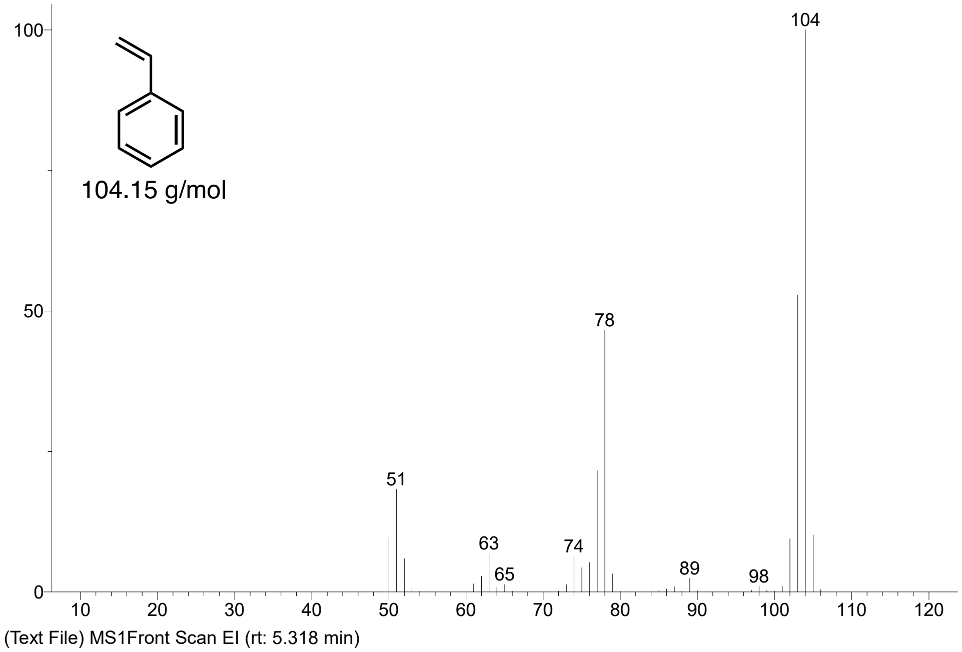

Figure S44. Mass spectrum of Fig. S37 at 5.32 min.

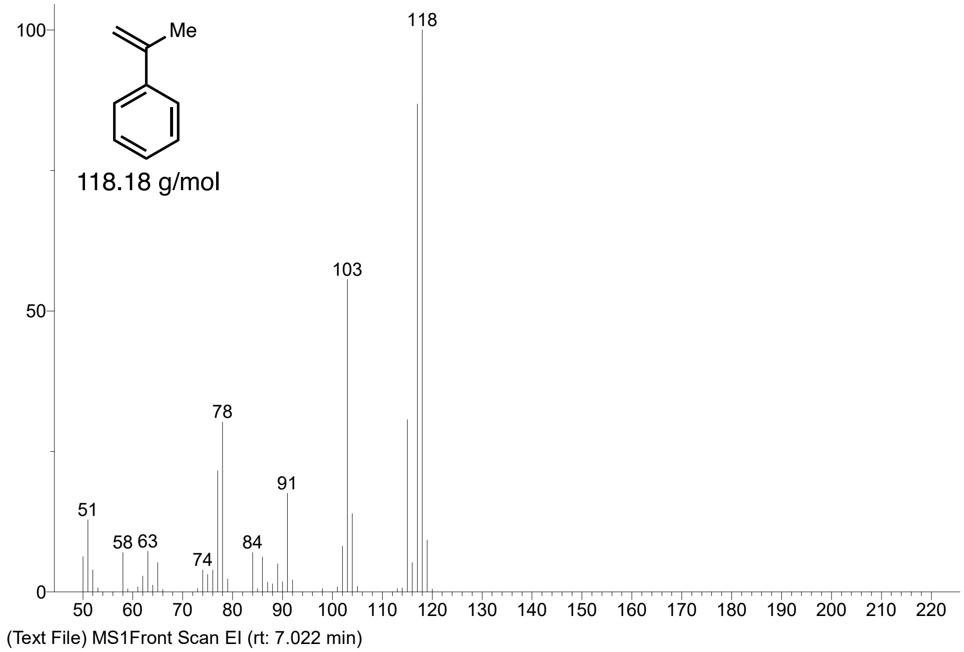

Figure S45. Mass spectrum of Fig. S37 at 7.02 min.

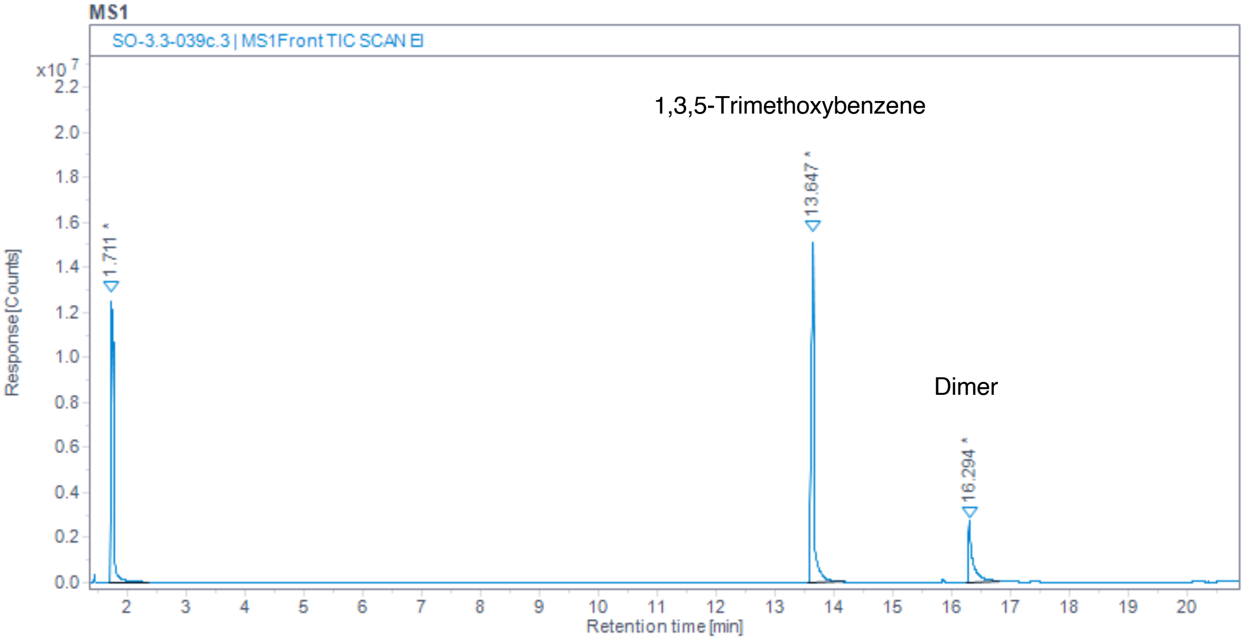

Figure S46. GC-MS of PS-CB$_{1.1}$ after photothermal depolymerization under vacuum (condenser).

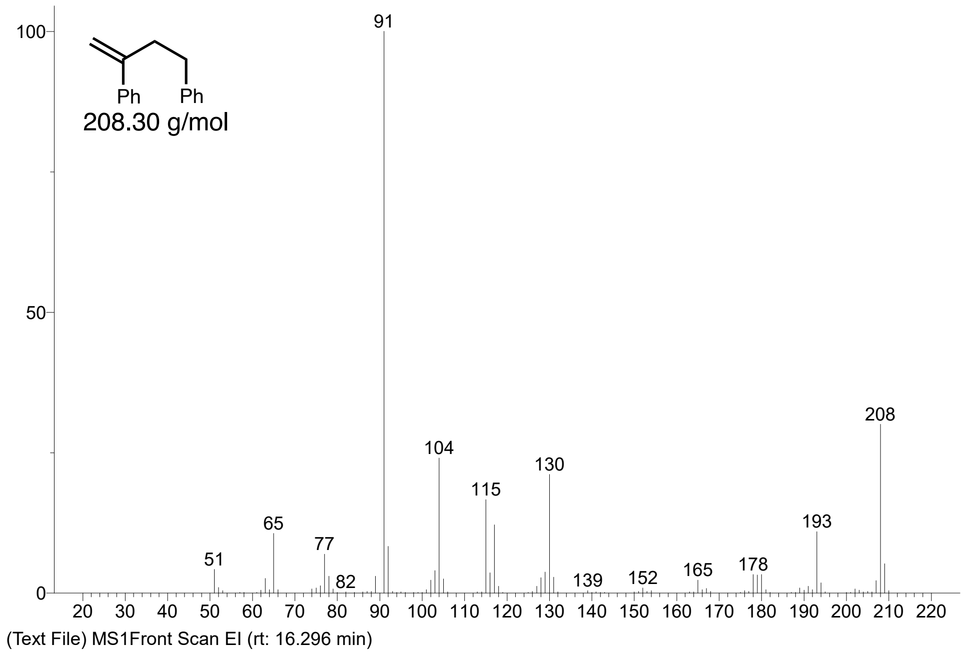

Figure S47. Mass spectrum of Fig. S41 at 16.29 min.

Procedure for Carbon Black Loading for Photothermal Depolymerization

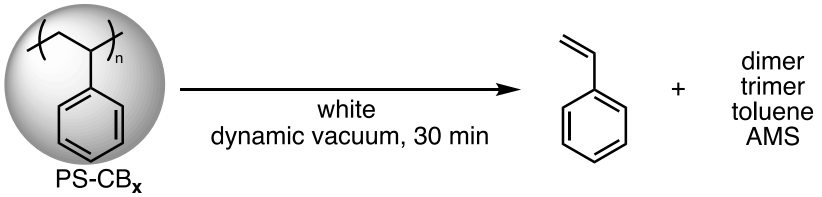

The procedure was same as the procedure for photothermal depolymerization under dynamic vacuum, except using different PS-CB$_x$ samples (50 mg). The depolymerization results were summarized, and GPC of the depolymerized samples were shown below.

Table S6. Results of carbon black loading for photothermal depolymerization.

Entry	PS-CB$_x$	M_n (kDa)	$Đ$	styrene (%)	dimer (%)	trimer (%)	toluene (%)	AMS (%)[a]	leftover PS (%)	mass recov. (%)
1	PS-CB$_{0.6}$	18.0	2.80	26.4	2.6	9.9	0.5	0.3	58.8	98.5
2	PS-CB$_{3.1}$	20.0	4.98	47.7	5.6	14.7	0.8	0.3	24.2	93.3
3	PS-CB$_{5.4}$	24.9	7.52	56.8	5.8	18.9	0.8	0.5	10.6	93.4
4	PS-CB$_{5.4}$	25.0	5.54	39.6	4.8	12.6	0.8	0.8	35.7	94.3

All % with regard to styrene repeating units
[a]Alpha-methylstyrene (AMS)

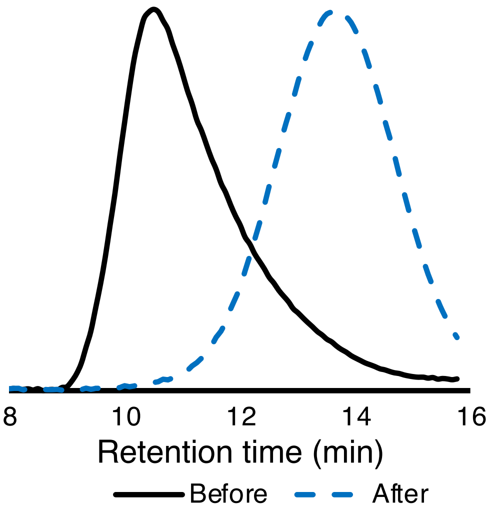

Figure S48. GPC of PS-CB$_{0.6}$ after photothermal depolymerization.

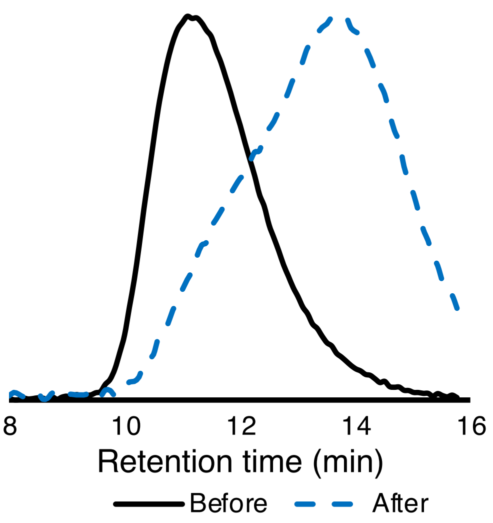

Figure S49. GPC of PS-$CB_{3.1}$ after photothermal depolymerization.

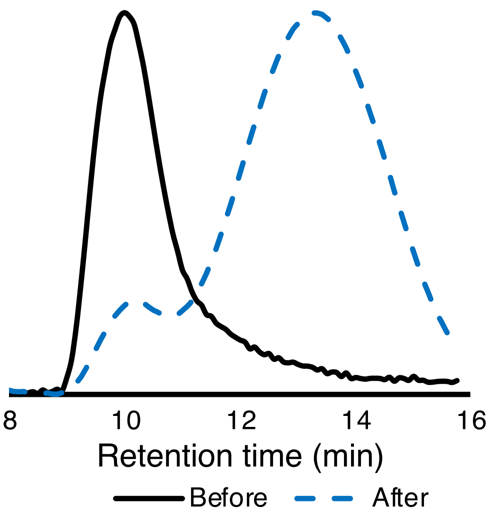

Figure S50. GPC of PS-$CB_{5.4}$ after photothermal depolymerization.

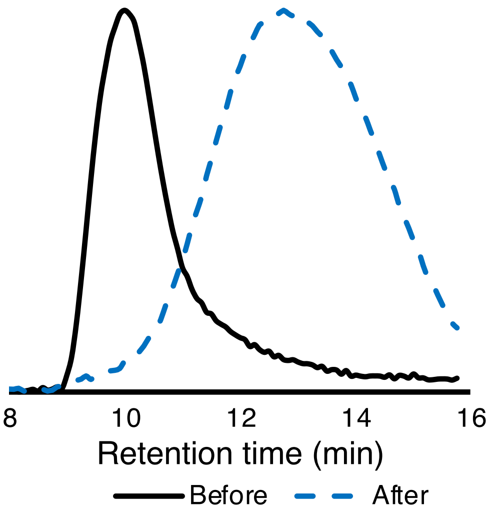

Figure S51. GPC of PS-$CB_{5.4}$ + CB (10 wt %) after photothermal depolymerization.

Procedure for Metal Catalyst Test for Photothermal Depolymerization

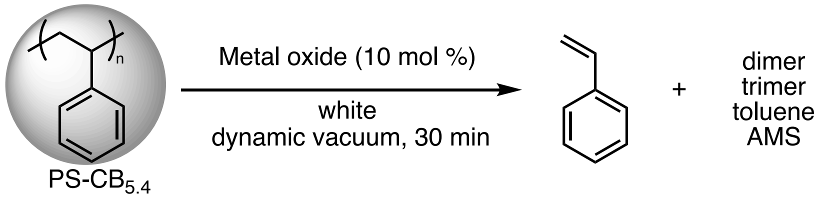

The procedure was slightly modified from the procedure for photothermal depolymerization under dynamic vacuum. For the sample preparation, PS-$CB_{5.4}$ (50 mg, carbon black = 5.4 wt %) and metal oxide (10 mol %) were added to the vial and mixed thoroughly with a spatula. The depolymerization results were summarized, and GPC of the depolymerized samples were shown below.

Table S7. Results of metal catalyst test for photothermal depolymerization.

Entry	Metal oxide	M_n (kDa)	*Đ*	styrene (%)	dimer (%)	trimer (%)	toluene (%)	AMS (%)[a]	leftover PS (%)	mass recov. (%)
1	ZnO	17.2	6.16	50.5	4.2	15.0	1.1	0.5	18.0	89.3
2	BaO	41.4	13.06	35.4	3.6	12.0	0.5	0.3	28.0	79.8
3	CaO	38.9	9.48	34.9	2.6	9.6	0.8	0.3	39.6	87.8
4	MgO	39.4	7.52	45.2	4.2	15.9	0.8	0.5	22.5	89.1

All % with regard to styrene repeating units
[a]Alpha-methylstyrene (AMS)

*These metal co-catalysts (ZnO, BaO, CaO, and MgO) are frequently used in catalytic pyrolysis of polystyrene to lower the bulk temperature.[5–8] However, these co-catalysts worsened the yield of styrene under photothermal depolymerization conditions. We hypothesize these metals may scatter the light and interfere with carbon black light absorption, thereby making the photothermal conversion of carbon black less effective.

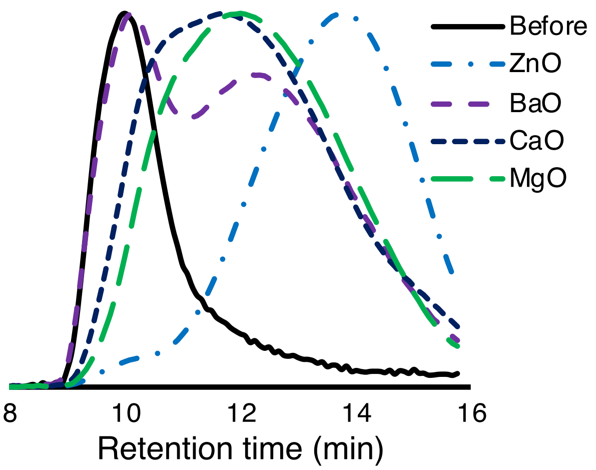

Figure S52. GPC of photothermal depolymerization with metal oxide.

Procedure for Control Experiments for Photothermal Depolymerization

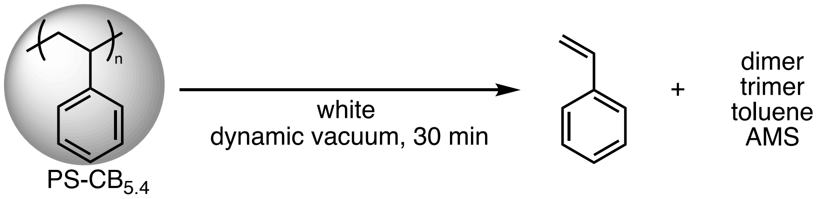

The procedure was slightly modified from the procedure for photothermal depolymerization under dynamic vacuum. PS-$CB_{5.4}$ (50 mg, carbon black = 5.4 wt %) was used, and depolymerization time was 30 min. The deviations were listed below. The depolymerization results were summarized, and GPC of the depolymerized samples were shown below.

Table S8. Results of controlled experiments for photothermal depolymerization.

Entry	Deviation	M_n (kDa)	$Đ$	styrene (%)	dimer (%)	trimer (%)	toluene (%)	AMS (%)[a]	leftover PS (%)	mass recov. (%)
1	Distance doubled	34.2	5.72	47.3	6.8	17.4	0.8	0.5	22.2	95.0
2	white LED (50 W)	51.1	6.69	11.9	1.6	5.4	0.3	0.3	78.7	98.2

All % with regard to styrene repeating units
[a]Alpha-methylstyrene (AMS)

*Reducing the light intensity resulted in a decreased yield of styrene. All these experiments reveal that high-intensity light and light absorptivity of photothermal agents are crucial to generate sufficient thermal energy for fast polystyrene depolymerization.

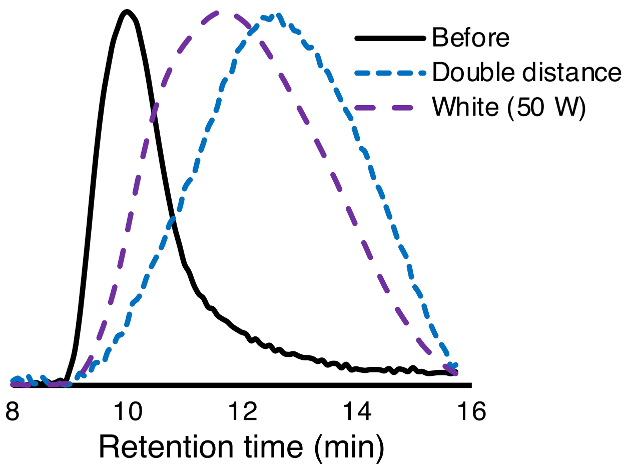

Figure S53. GPC of controlled experiments after photothermal depolymerization.

Procedure for Pure PS & CB Mixture for Photothermal Depolymerization

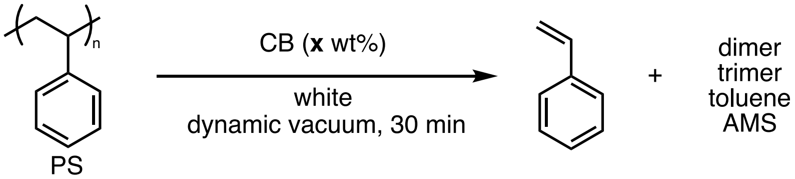

The procedure was slightly modified from the procedure for photothermal depolymerization under dynamic vacuum. The desired amount of CB and pure PS were thoroughly grounded in a mortal with a pestle. The mixture (50 mg) was used, and depolymerization time was 30 min. The depolymerization results were summarized, and GPC of the depolymerized samples were shown below.

Table S9. Results of Pure PS & CB mixture for photothermal depolymerization.

Entry	**x** wt%	M_n (kDa)	*Đ*	styrene (%)	dimer (%)	trimer (%)	toluene (%)	AMS (%)[a]	leftover PS (%)	mass recov. (%)
1	0	85.6	6.86	0	0	0	0	0	>99	>99
2	1.1	15.5	2.71	39.7	5.6	13.5	0.5	0.3	31.6	91.2
3	3.1	16.0	2.44	44.1	6.2	13.2	0.8	0.3	27.9	92.5
4	5.4	23.8	9.61	55.2	9.0	17.4	1.1	0.5	7.1	90.3

All % with regard to styrene repeating units
[a]Alpha-methylstyrene (AMS)

*When screening various PS-CB$_x$ in Fig. 2B, these samples had different M_n and dispersities, which prevents a fair comparison of depolymerization efficacy among different carbon black loadings. In contrast, powder mixed samples with different amounts of carbon black had the same M_n and dispersity and, thus, is a better comparison of depolymerization rate. It turned out that whether the molecular weight of polystyrene is different or same, 5.4 wt % CB yielded the highest amount of styrene, indicating photothermal depolymerization depends more on the amount of photothermal agent not on the size of polystyrene.

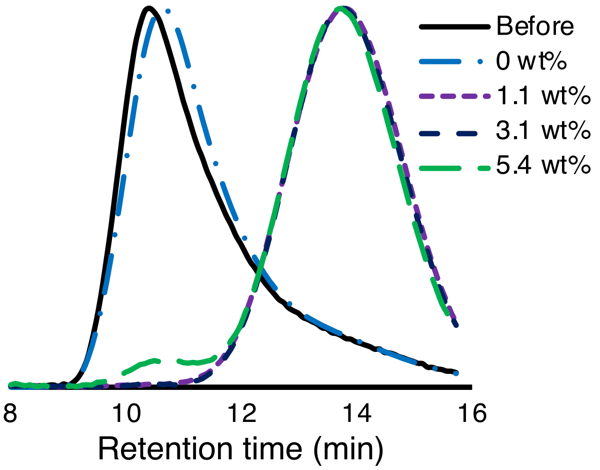

Figure S54. GPC of Pure PS & CB mixture after photothermal depolymerization.

Procedure for Time Course Study for Photothermal Depolymerization

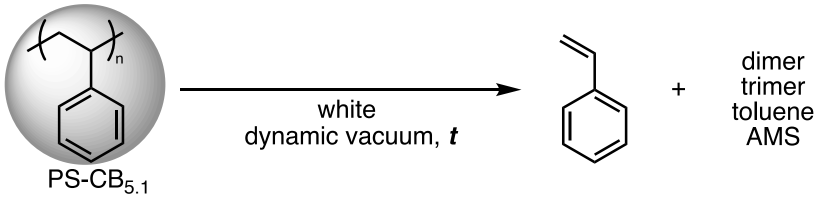

The procedure was slightly modified from the procedure for photothermal depolymerization under dynamic vacuum. PS-$CB_{5.5}$ (50 mg, carbon black = 5.5 wt %) was used, and depolymerization time was varied from 5 min to 60 min. The depolymerization results were summarized, and GPC of the depolymerized samples were shown below.

Table S10. Results of time course study for photothermal depolymerization.

Entry	***t*** (min)	M_n (kDa)	*Đ*	mass recov. (%)
	5	27.0	8.02	95.0
1	5	29.6	7.28	95.2
	5	30.7	7.15	95.0
	10	23.7	5.90	90.7
2	10	27.7	5.91	91.0
	10	28.4	6.47	92.6
	15	22.3	5.57	92.6
3	15	19.0	5.00	93.1
	15	19.2	4.18	92.6
	30	18.5	4.15	88.9
4	30	19.3	4.97	91.0
	30	17.6	3.97	92.0
	60	18.3	4.33	91.0
5	60	18.5	4.51	91.0
	60	22.4	5.74	89.9

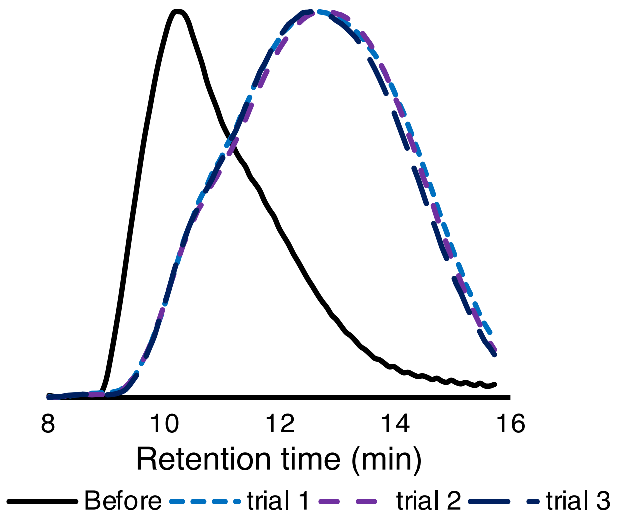

Figure S55. GPC of PS-CB$_{5.5}$ after 5 min photothermal depolymerization.

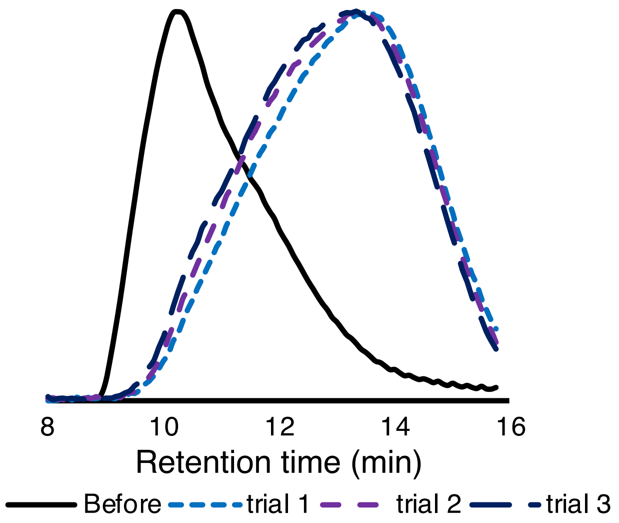

Figure S56. GPC of PS-CB$_{5.5}$ after 10 min photothermal depolymerization.

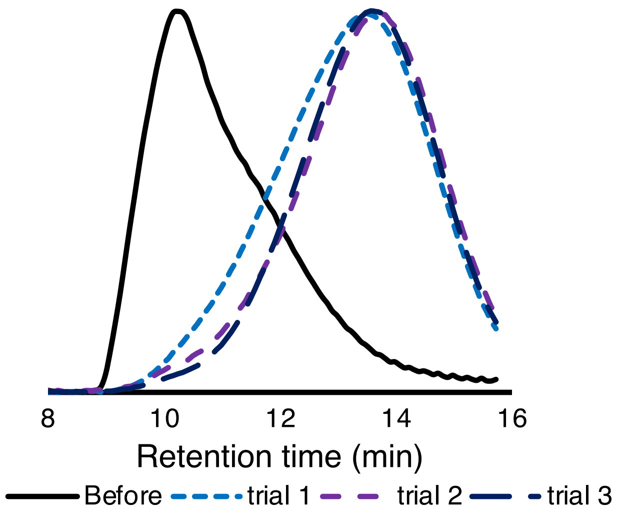

Figure S57. GPC of PS-$CB_{5.5}$ after 15 min photothermal depolymerization.

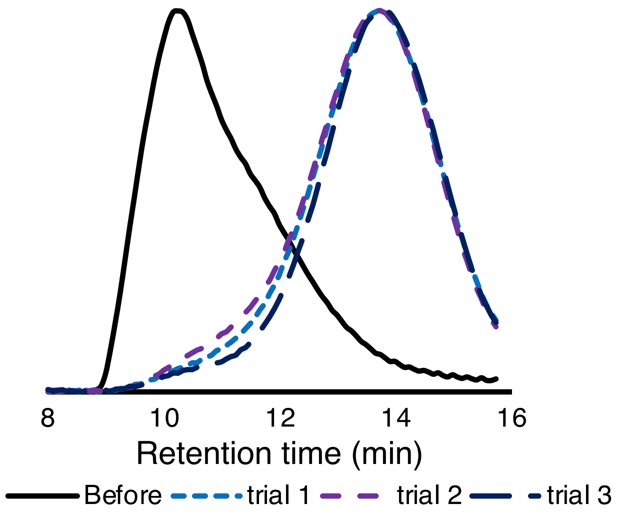

Figure S58. GPC of PS-$CB_{5.5}$ after 30 min photothermal depolymerization.

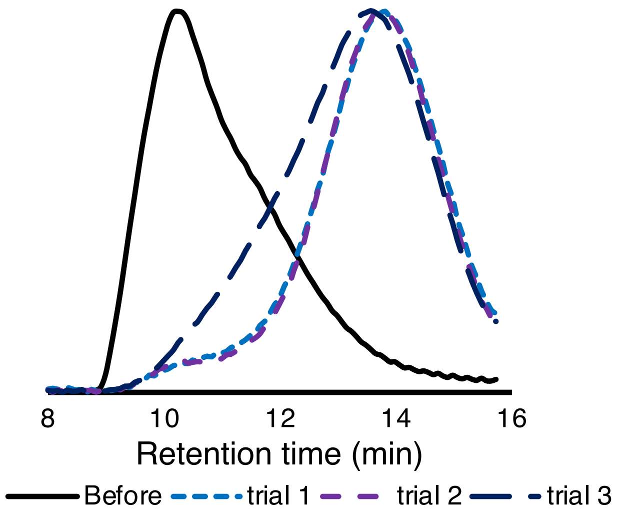

Figure S59. GPC of PS-CB$_{5.5}$ after 60 min photothermal depolymerization.

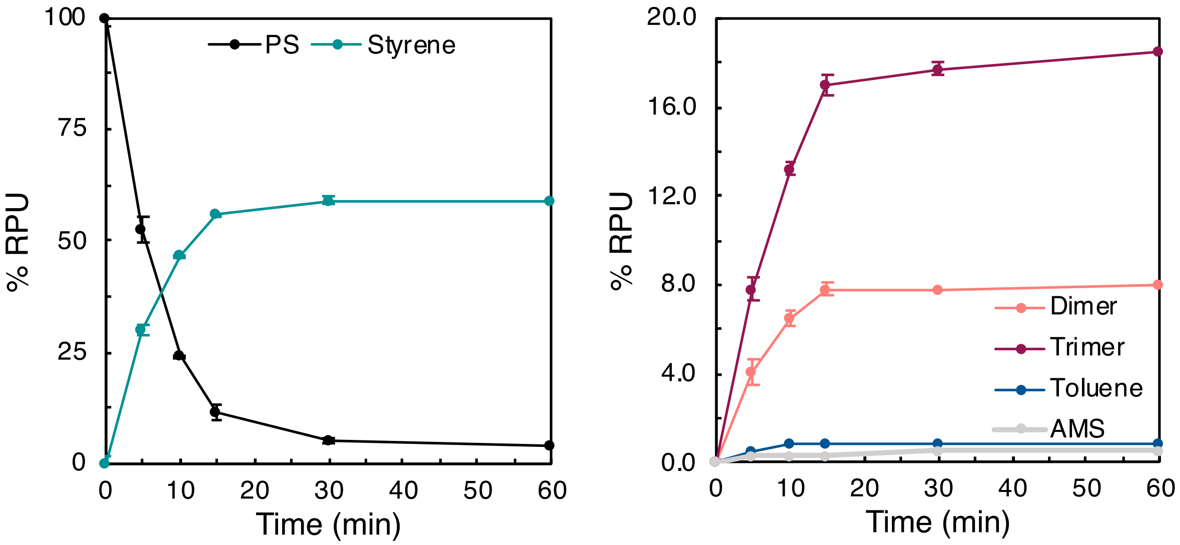

Figure S60. Scatter plot for the results of kinetic study for photothermal depolymerization.
*Time course studies were conducted to evaluate the evolution of small molecule generation throughout multiple time points. All small molecules are rapidly produced up to 15 min and started plateauing at 30 min. Polystyrene showed the exactly opposite trend, at which its amount disappeared very fast up to 15 min and became stabilized at 30 min. Each experiment was triplicated and showed the excellent reproducibility.

Procedure for TEMPO-PS for Photothermal Depolymerization

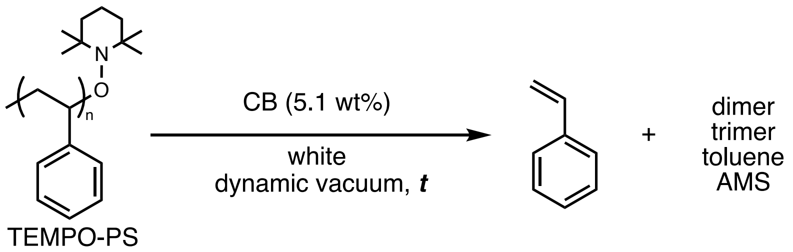

The procedure was slightly modified from the procedure for photothermal depolymerization under dynamic vacuum. The desired amount of CB and TEMPO-lated PS were thoroughly ground using a mortar and pestle. The mixture (50 mg, carbon black = 5.1 wt %) was used. The depolymerization results were summarized, and GPC of the depolymerized samples were shown below. The un-normalized GPC trace is included in the manuscript.

Table S11. Results of TEMPO-PS for photothermal depolymerization.

Entry	***t*** (min)	M_n (kDa)	*Đ*	styrene (%)	dimer (%)	trimer (%)	toluene (%)	AMS (%)[a]	leftover PS (%)	mass recov. (%)
1	5	14.1	1.38	23.2	3.6	6.3	0.3	0.3	64.5	98.2
2	10	14.1	1.37	28.4	4.2	8.7	0.5	0.3	54.8	96.9
3	15	13.9	1.38	31.3	4.2	9.6	0.5	0.3	49.3	95.2
4	30	13.9	1.38	37.7	4.8	12.0	0.5	0.5	39.0	94.5
5	60	13.7	1.39	38.7	4.2	12.6	0.5	0.5	34.8	91.3

All % with regard to styrene repeating units
[a]Alpha-methylstyrene (AMS)

*We discovered that low molecular weight polystyrene ($M_n \leq 20$ kDa) depolymerization resulted in more diminished yield of styrene (~ 40%), indicating that polystyrene chain length should be large enough ($M_n \geq 80$ kDa) to be depolymerized efficiently. These data suggest a random chain scission mechanism followed by chain-end unzipping, rather than chain end depolymerization.

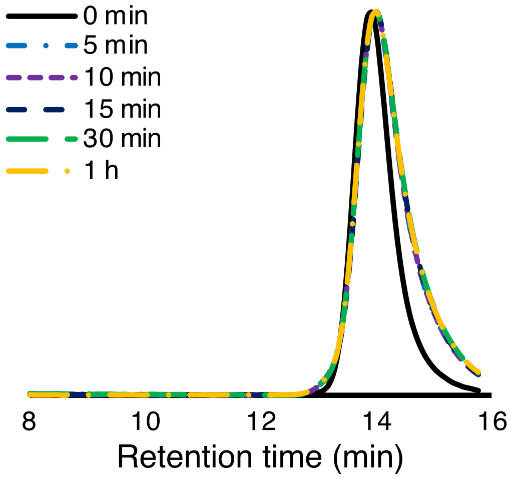

Figure S61. GPC of TEMPO-PS after photothermal depolymerization.

Procedure for DeTEMPO-PS for Photothermal Depolymerization

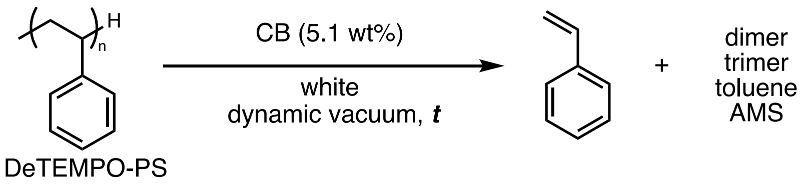

The procedure was slightly modified from the procedure for photothermal depolymerization under dynamic vacuum. The desired amount of CB and DeTEMPO-lated PS were thoroughly grounded in a mortal with a pestle. The mixture (50 mg, carbon black = 5.1 wt %) was used. The depolymerization results were summarized, and GPC of the depolymerized samples were shown below. The un-normalized GPC trace is included in the manuscript.

Table S12. Results of DeTEMPO-PS for photothermal depolymerization.

Entry	***t*** (min)	M_n (kDa)	*Đ*	styrene (%)	dimer (%)	trimer (%)	toluene (%)	AMS (%)[a]	leftover PS (%)	mass recov. (%)
1	5	15.4	1.37	24.8	3.7	6.3	0.5	0.3	63.0	98.5
2	10	15.0	1.38	29.2	4.2	8.7	0.5	0.3	50.8	93.8
3	15	14.8	1.40	31.3	4.7	9.5	0.5	0.3	48.5	94.8
4	30	15.1	1.38	38.2	4.7	11.9	0.5	0.5	38.7	94.6
5	60	15.2	1.38	38.5	4.2	11.9	0.5	0.5	34.2	89.8

All % with regard to styrene repeating units
[a]Alpha-methylstyrene (AMS)

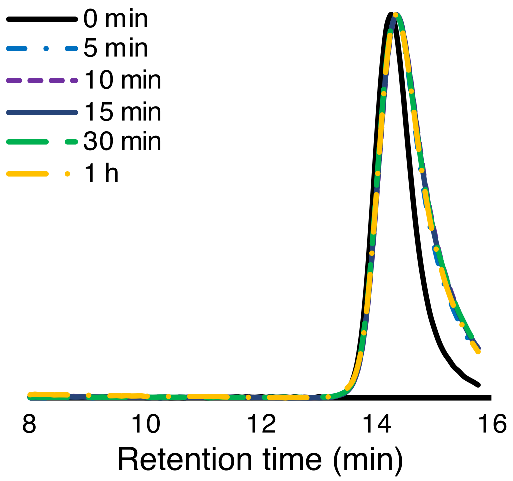

Figure S62. GPC of DeTEMPO-PS after photothermal depolymerization.

Procedure for Larger Scale Photothermal Depolymerization

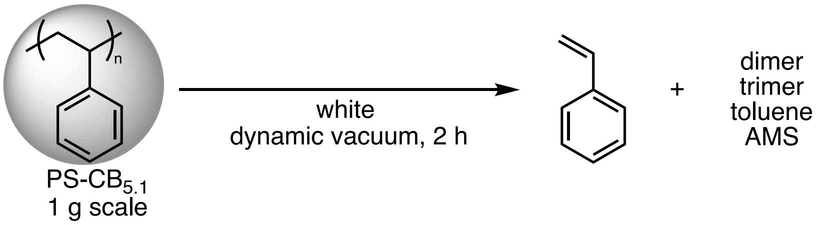

The procedure was slightly modified from the procedure for photothermal depolymerization under dynamic vacuum. PS-$CB_{5.1}$ (1 g, carbon black = 5.1 wt %) was used in a scintillation vial, and depolymerization time was 2 h. The depolymerization results were summarized, and GPC of the depolymerized samples were shown below.

Table S13. Results of photothermal depolymerization with 1 g scale.

Entry	M_n (kDa)	$Đ$	styrene (%)	dimer (%)	trimer (%)	toluene (%)	AMS (%)[a]	leftover PS (%)	mass recov. (%)
1	51.5	6.72	42.4	4.8	9.6	0.8	0.5	29.0	87.1

All % with regard to styrene repeating units
[a]Alpha-methylstyrene (AMS)

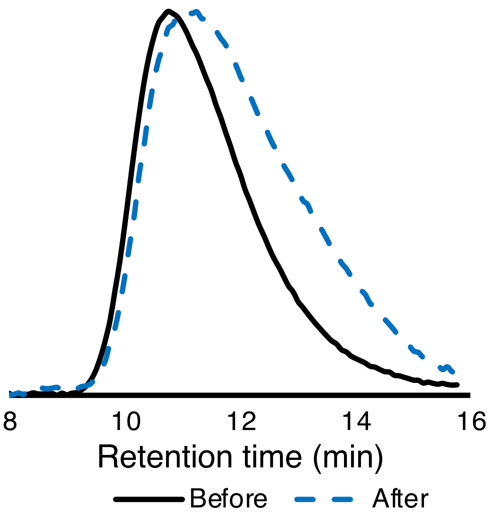

Figure S63. GPC of PS-$CB_{5.1}$ (1 g) after photothermal depolymerization.

Procedure for Repurposing Leftover PS-CB After Depolymerization

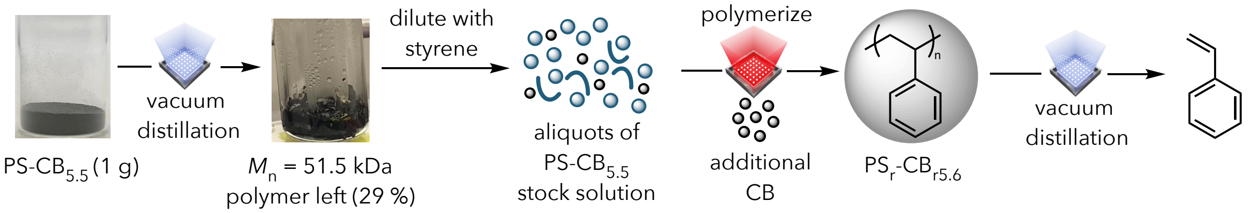

(Sample Preparation) PS-$CB_{5.5}$ (1 g, carbon black = 5.5 wt %, 55 mg) was photothermally depolymerized with the same condition as above (Procedure for High Scale Photothermal Depolymerization). In the reaction vial, styrene (0.1 mol, 11.5 mL, 10.35 g) was added and waited until leftover polystyrene was fully dissolved. (Assuming the mass of carbon black was not changed throughout the reactions, 55 mg of carbon black would be suspended in the stock solution).

(Synthesis) The procedure was slightly modified from the general procedure of polystyrene synthesis via photothermal effect with carbon black. Instead of pure styrene, the stock solution (10.0 mmol, 1.15 mL, 1.035g, carbon black = 5.5 mg) was used. The polymerization result and GPC of polystyrene were shown below. T_d and T_g of polystyrene were obtained from TGA and DSC. The data are listed below and spectrum was shown below.

Table S14. Results of repurposing leftover PS-CB.

Entry	M_n (kDa)	*Đ*	Yield (%)[a]	CB_{incorp} (wt %)	PS_r-CB_{rx}	T_d (˚C)	T_g (˚C)
1	106.4	4.43	82	0.6	PS_r-$CB_{r0.6}$	340.7	105.8
2[b]	139.5	5.60	85	5.6	PS_r-$CB_{r5.5}$	346.1	104.8

[a]Calculated by isolated product mass / (initial styrene mass + initial CB mass),
[b]Extra CB (46.3 mg) was added

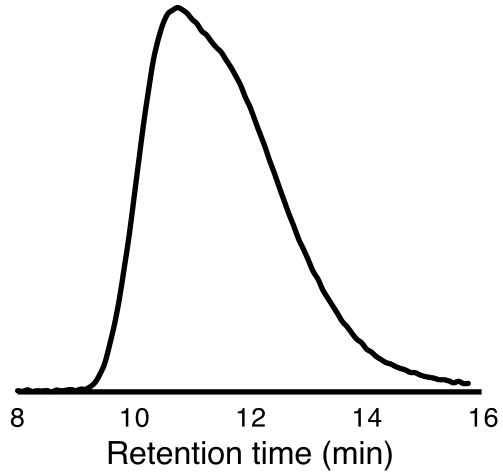

Figure S64. GPC of PS_r-$CB_{r0.6}$.

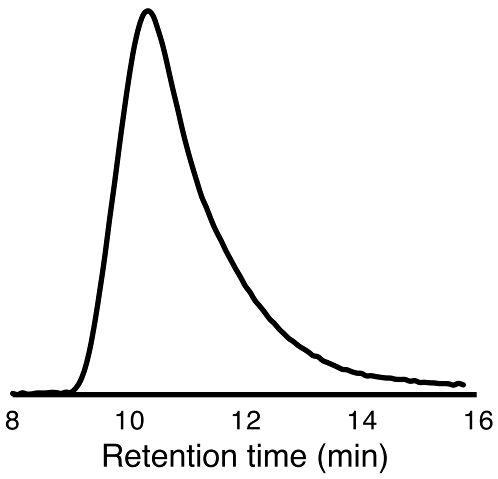

Figure S65. GPC of repurposed PS_r-$CB_{r5.6}$.

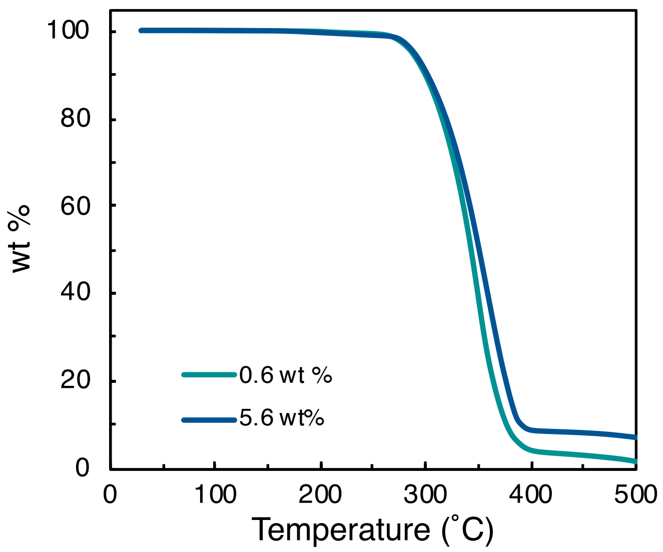

Figure S66. TGA of repurposed PS_r-CB_{rx}.

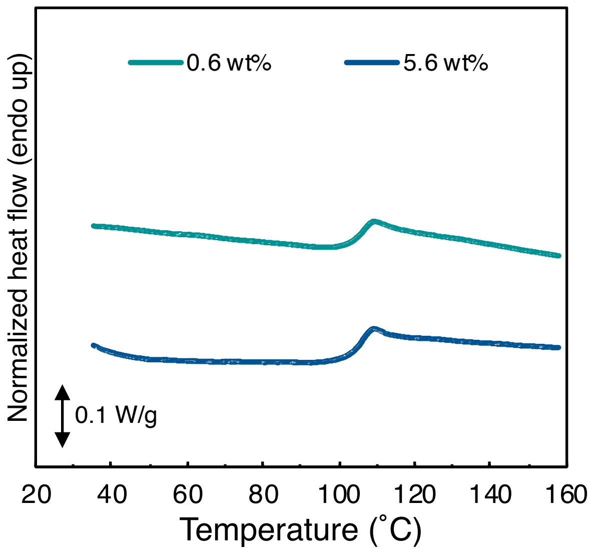

Figure S67. DSC of repurposed PS_r-CB_{rx}.

Procedure for Depolymerizing Repurposed PS$_r$-CB$_{rx}$

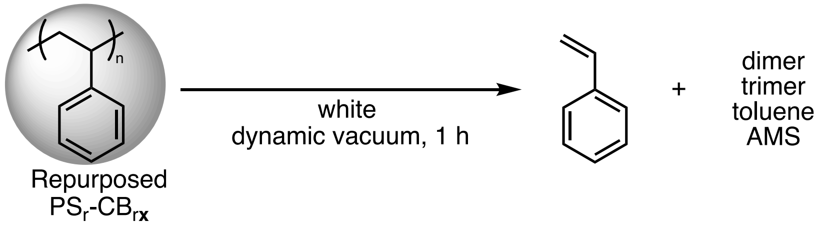

The procedure was slightly modified from the procedure for photothermal depolymerization under dynamic vacuum. Repurposed PS$_r$-CB$_{rx}$ (50 mg, carbon black = **x** wt %) was used, and depolymerization time was an hour. The depolymerization results were summarized, and GPC of the depolymerized samples were shown below.

Table S15. Results of photothermal depolymerization with repurposed PS$_r$-CB$_{rx}$.

Entry	**x** wt %	M_n (kDa)	$Đ$	styrene (%)	dimer (%)	trimer (%)	toluene (%)	AMS (%)[a]	leftover PS (%)	mass recov. (%)
1	0.6	16.5	3.62	33.2	6.0	15.0	0.8	0.3	37.2	92.5
2	5.6	20.6	6.65	58.3	8.0	19.2	0.8	0.5	4.5	91.3

All % with regard to styrene repeating units
[a]Alpha-methylstyrene (AMS)

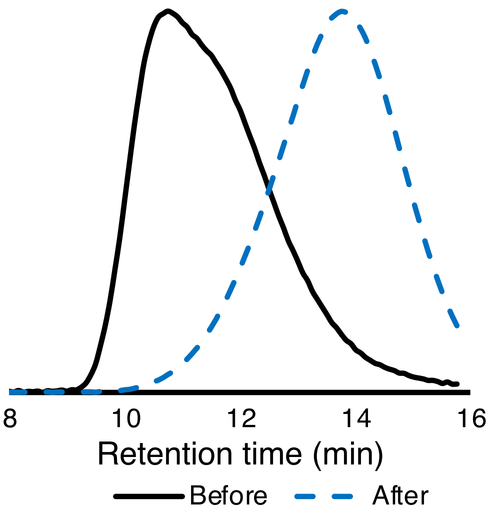

Figure S69. GPC of PS$_r$-CB$_{r0.6}$ after photothermal depolymerization.

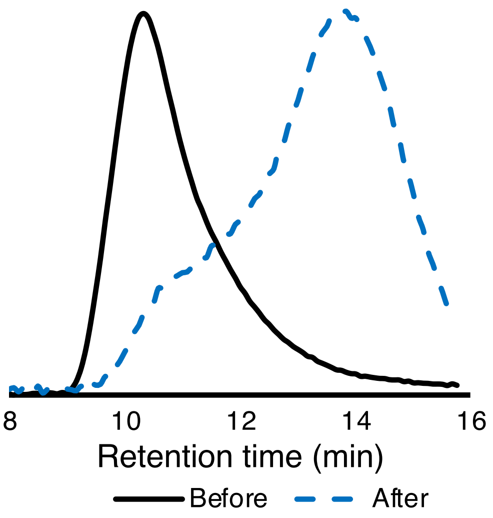

Figure S70. GPC of repurposed PS_r-$CB_{r5.6}$ after photothermal depolymerization.

Procedure for Photothermal Depolymerization with Recycled Carbon Black

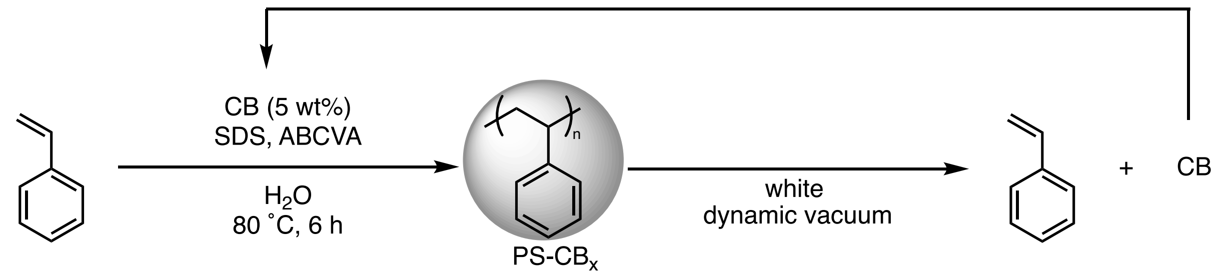

(Synthesis) The procedure was modified from the literature.[2] Deionized H_2O (148 mL) was added into a large round-bottom flask, sealed with a cap equipped with a rubber septum, and sparged with N_2 for 25 min. While waiting, styrene was degassed under N_2 for 20 min. ABCVA (0.148 mmol, 44.3 mg, 1 equiv.), SDS (11.2 mmol, 3.07 g, 13.2 equiv.), carbon black (765 mg, 5 wt %) were added into the scintillation vial. While stirring the aqueous mixture, degassed styrene (148 mmol, 17.0 mL, 15.5 g, 1000 equiv.) was added. The reaction mixture was stirred at 80 °C for 6 hours. The sample was removed from the heat source and cooled to room temperature. The polymer was precipitated using cold MeOH (1 L). The polymer precipitates were collected through vacuum filtration (repeat filtration one more time if necessary), followed by washing with a copious amount of methanol. The polymer was dried under high vacuum overnight. The polymerization result and GPC of polystyrene were shown below. T_d and T_g of polystyrene were obtained from TGA and DSC. The data are listed below and spectrum was shown below.

(Depolymerization) The procedure was slightly modified from the procedure for photothermal depolymerization under dynamic vacuum. PS-CB (50 mg) from the previous reaction was used. The depolymerization results were summarized, and GPC of the depolymerized samples were shown below.

(Carbon black recovery) A series of PS-CB (0.8 – 1.2 g) samples from the previous reaction were photothermally depolymerized (see Procedure for Larger Scale Photothermal Depolymerization) until all PS-CB was used. After photothermal depolymerization of all PS-CB samples, DCM was added to reaction vials with PS-CB and waited for a few hours until all polymers were dissolved. The reaction mixture was centrifuged for 15 min with 4400 rpm. The solution was decanted, and CB residues were collected in a clean vial. DCM was added to CB residues and waited for several minutes until small amounts of polymers were dissolved. CB was filtered via vacuum filtration, collected, and dried in a vacuum oven at 70 ˚C overnight. A small amount of CB was used for an NMR analysis to ensure that there was no polymer left. The recycled CB was ground and used for another round of synthesis and depolymerization. The recycled carbon black was used as 5 wt % relative to the mass of styrene for synthesis.

Table S16. Results of polymerization with recycled CB.

CB cycle #	Sample	M_n (kDa)	$Đ$	Yield (%)[a]	CB_{incorp} (wt %)	T_d (˚C)	T_g (˚C)
1	PS-CB_1	122.5	6.92	96	4.9	357.8	107.0
2	PS-CB_2	109.7	3.56	89	5.4	354.3	107.1
3	PS-CB_3	120.7	5.84	94	5.1	353.2	107.9
4	PS-CB_4	171.6	4.55	81	5.8	357.2	105.9
5	PS-CB_5	106.2	4.98	96	5.2	342.0	106.7

[a]Calculated by isolated product mass / (initial styrene mass + initial CB mass)

Table S17. Results of photothermal depolymerization with recycled CB.

Entry	Sample	M_n (kDa)	$Đ$	styrene (%)	dimer (%)	trimer (%)	toluene (%)	AMS (%)[a]	leftover PS (%)	mass recov. (%)
1	PS-CB_1	28.1	5.76	55.2	7.4	15.0	0.8	0.3	10.3	89.0
2	PS-CB_2	17.3	3.56	51.8	6.8	17.4	0.8	0.3	10.3	87.4
3	PS-CB_3	18.6	4.33	55.3	7.4	16.5	0.8	0.5	9.9	90.4
4	PS-CB_4	17.8	3.10	54.9	6.4	17.4	1.1	0.5	8.5	88.8
5	PS-CB_5	15.8	2.74	54.3	6.4	18.3	0.8	0.5	15.6	95.6

All % with regard to styrene repeating units
[a]Alpha-methylstyrene (AMS)

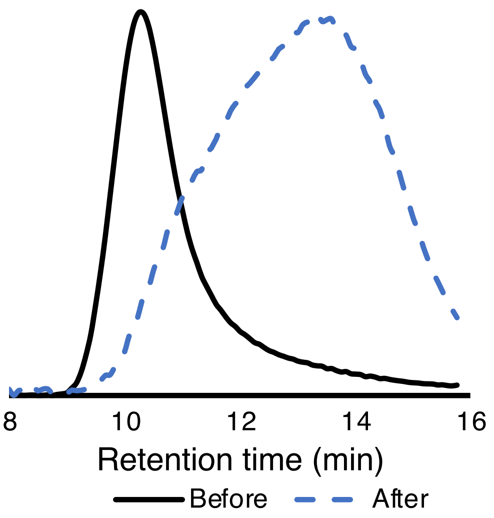

Figure S71. GPC of PS-CB_1 after photothermal depolymerization.

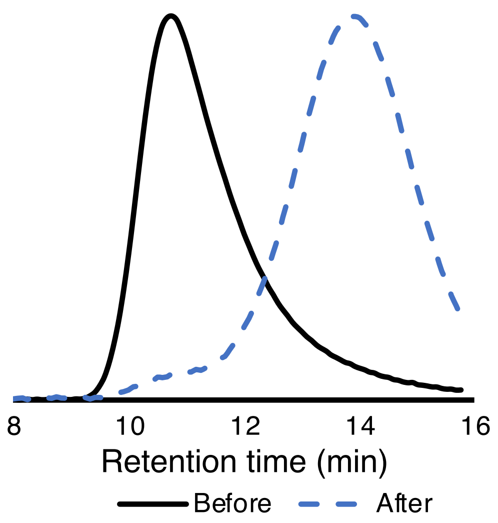

Figure S72. GPC of PS-CB_2 after photothermal depolymerization.

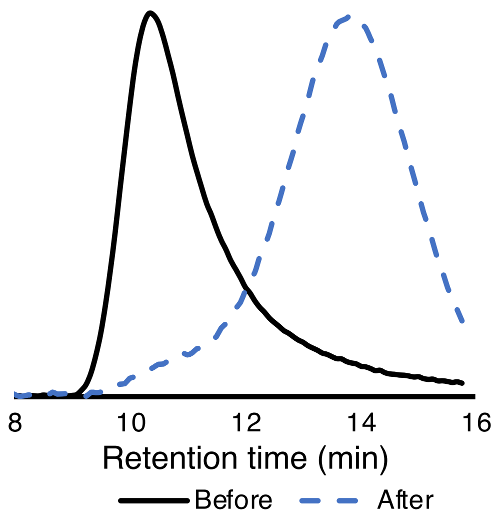

Figure S73. GPC of PS-CB_3 after photothermal depolymerization.

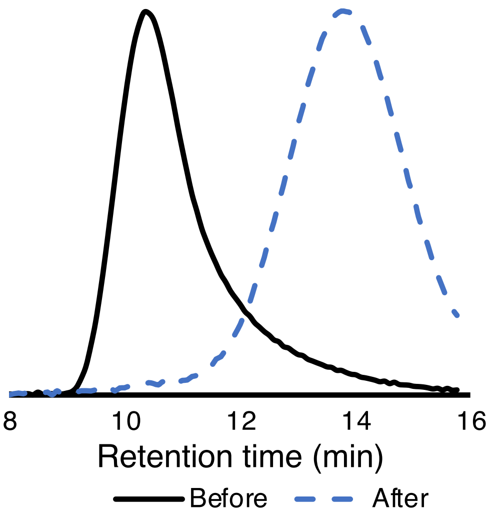

Figure S74. GPC of PS-CB_4 after photothermal depolymerization.

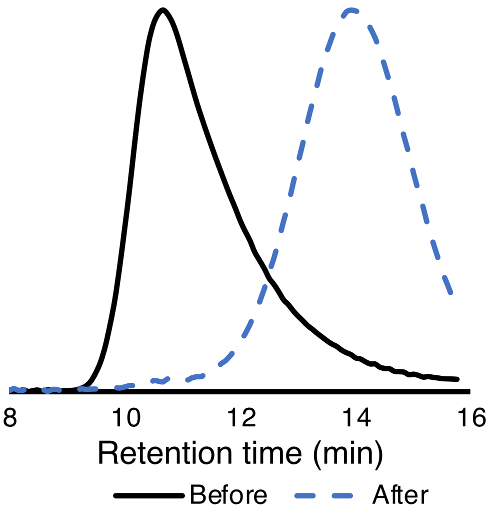

Figure S75. GPC of PS-CB_5 after photothermal depolymerization.

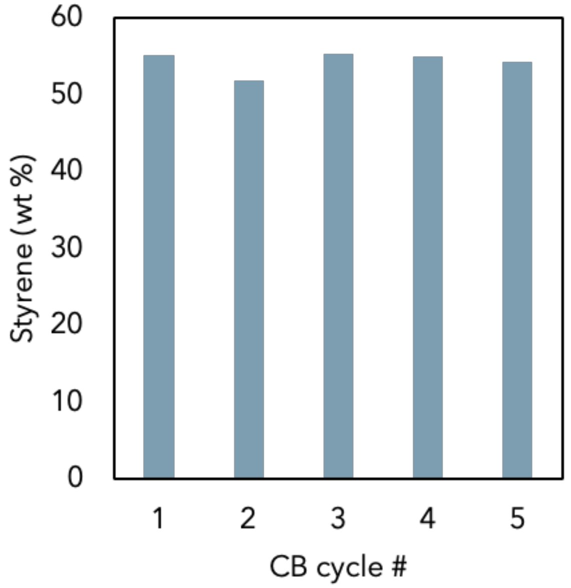

Figure S76. Isolated styrene yield after photothermal depolymerization at each CB cycle.

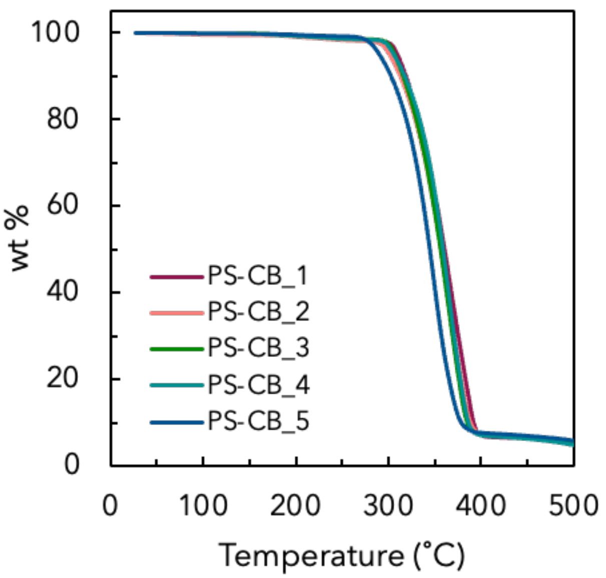

Figure S77. TGA of PS-CB_x (x is the number of times CB is used).

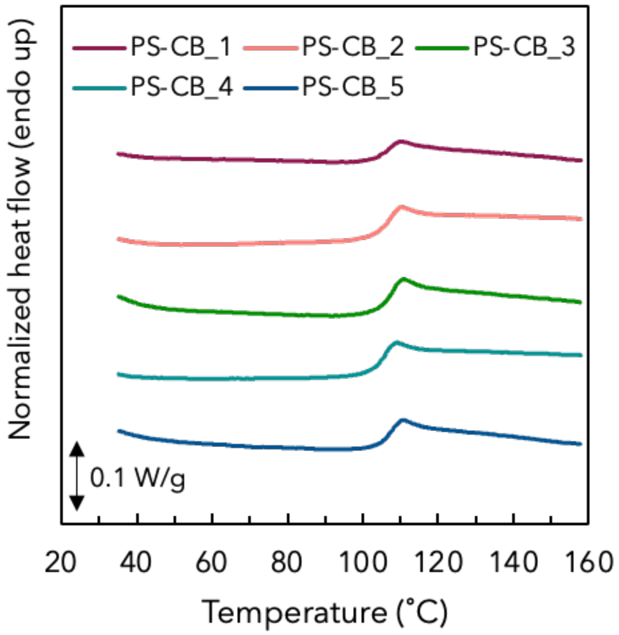

Figure S78. DSC of PS-CB_x (x is the number of times CB is used).

Procedure for Photothermal Depolymerization with Aluminum Foil Control

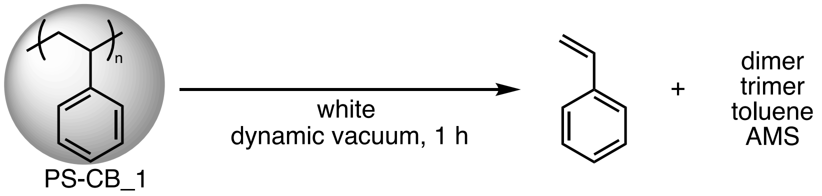

The procedure was slightly modified from the procedure for photothermal depolymerization under dynamic vacuum. PS-CB_1 (50 mg, recycled carbon black = 4.9 wt %) was used, and depolymerization time was an hour. Aluminum foil was wrapped around the reaction vial throughout the duration of the reaction. The depolymerization results were summarized, and GPC of the depolymerized samples were shown below.

Table S18. Results of photothermal depolymerization with aluminum foil control.

Entry	M_n (kDa)	$Đ$	styrene (%)	dimer (%)	trimer (%)	toluene (%)	AMS (%)[a]	leftover PS (%)	mass recov. (%)
1	250.0	2.83	0	0	0	0	0	> 99	> 99

[a]Alpha-methylstyrene (AMS)

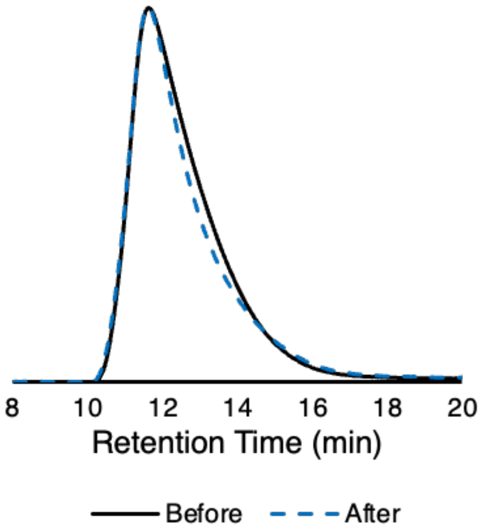

Figure S79. GPC of PS-CB_1 after photothermal depolymerization with aluminum foil control. *Measurements taken on Agilent GPC.

Procedure for Photothermal Depolymerization with Recovered Styrene

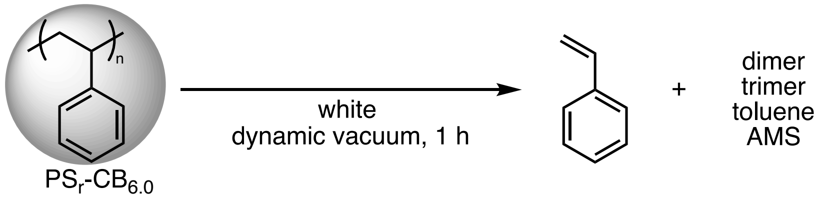

(Procedure for recovering styrene) A few reactions for 1-gram photothermal depolymerization of PS-CB were conducted (see Procedure for Large Scale Photothermal Depolymerization). Styrene was collected and passed through a plug of basic alumina before using for the subsequent polymerization.

(Synthesis) The procedure was the same as the general procedure of polystyrene synthesis via photothermal effect with carbon black, except using recovered styrene. GPC of polystyrene was shown below. T_d and T_g of polystyrene were obtained from TGA and DSC. TGA and DSC spectra were shown below. (Yield: 80 %, M_n = 85.3 kDa, $Đ$ = 3.13, CB_{incorp} = 6.0 wt %, T_d = 360.0 °C, T_g = 105.5 °C).

(Depolymerization) The procedure was slightly modified from the procedure for photothermal depolymerization under dynamic vacuum. PS_r-$CB_{6.0}$ (50 mg, carbon black = 6.0 wt %) was used, and depolymerization time was an hour. The depolymerization results were summarized, and GPC of the depolymerized samples were shown below.

Table S19. Results of photothermal depolymerization with recovered styrene.

Entry	M_n (kDa)	$Đ$	styrene (%)	dimer (%)	trimer (%)	toluene (%)	AMS (%)[a]	leftover PS (%)	mass recov. (%)
1	18.6	3.06	58.2	7.4	18.3	0.8	1.3	4.5	90.5

All % with regard to styrene repeating units
[a]Alpha-methylstyrene (AMS)

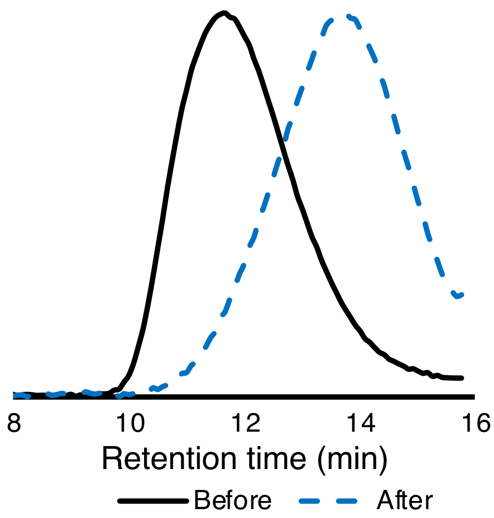

Figure S80. GPC of PS_r-$CB_{6.0}$ after photothermal depolymerization.

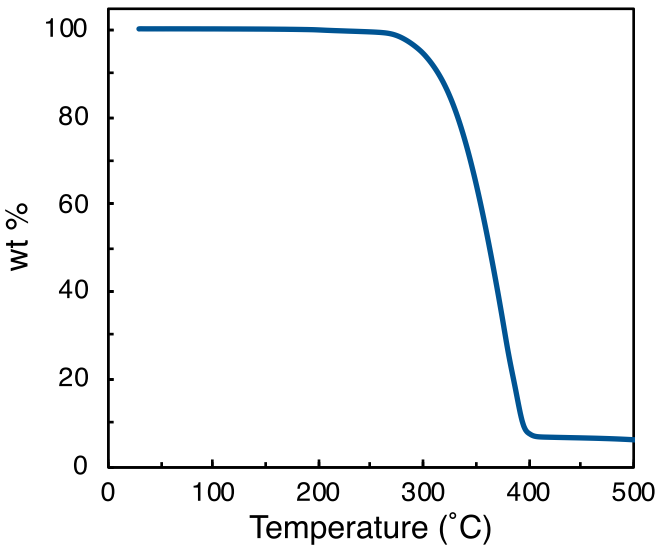

Figure S81. TGA of PS_r-$CB_{6.0}$.

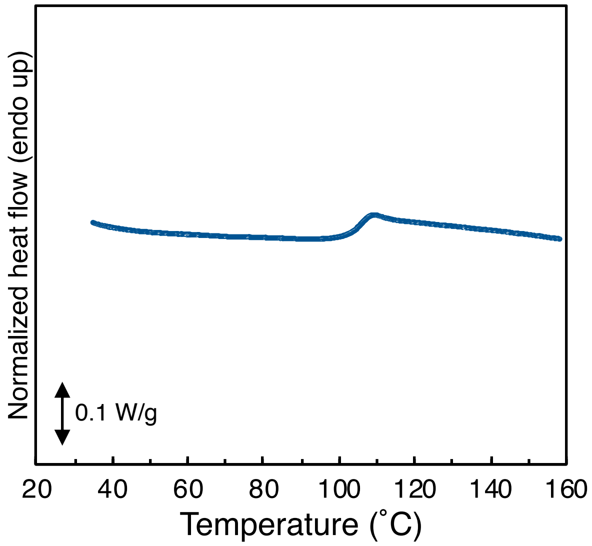

Figure S82. DSC of PS_r-$CB_{6.0}$.

Procedure for HIPS-CB Photothermal Depolymerization

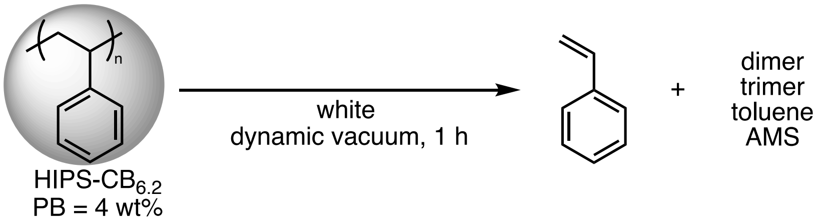

The procedure was slightly modified from the procedure for photothermal depolymerization under dynamic vacuum. HIPS-$CB_{6.2}$ (50 mg, carbon black = 6.2 wt %) was used, and depolymerization time was an hour. The depolymerization results were summarized, and GPC of the depolymerized samples were shown below.

Table S20. Results of photothermal depolymerization with HIPS-$CB_{6.2}$.

Entry	M_n (kDa)	$Đ$	styrene (%)	dimer (%)	trimer (%)	toluene (%)	AMS (%)[a]	leftover PS (%)	mass recov. (%)
1	17.9	3.03	50.8	5.0	16.8	1.7	1.4	15.3	91.0

All % with regard to styrene repeating units
[a]Alpha-methylstyrene (AMS)

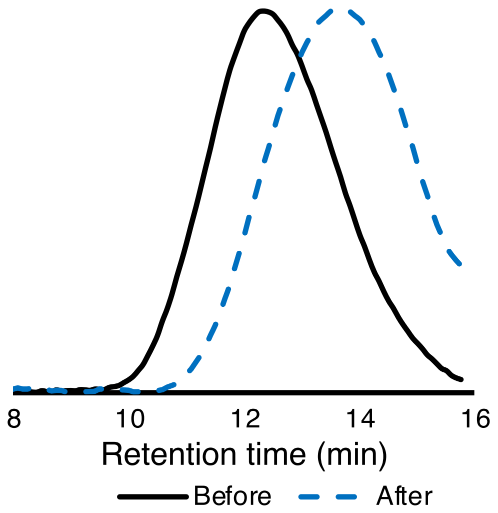

Figure S83. GPC of HIPS-$CB_{6.2}$ after photothermal depolymerization.

Procedure for PS-*co*-PAN-CB Photothermal Depolymerization

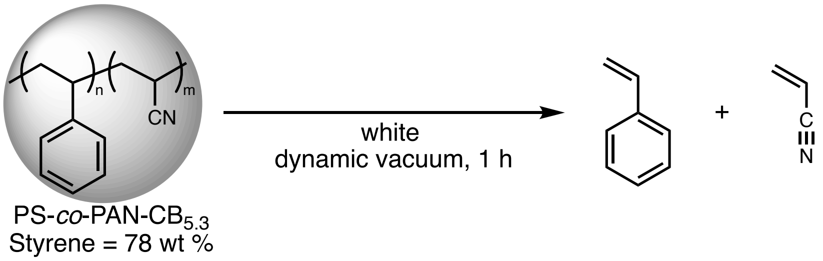

The procedure was slightly modified from the procedure for photothermal depolymerization under dynamic vacuum. PS-*co*-PAN-$CB_{5.3}$ (50 mg, carbon black = 5.3 wt %) was used, and depolymerization time was an hour. Liquid nitrogen was used instead of the dry ice acetone bath for the receiving flask. The depolymerization results were summarized, and GPC of the depolymerized samples were shown below.

Table S21. Results of photothermal depolymerization with PS-*co*-PAN-$CB_{5.3}$.

Entry	M_n (kDa)	$Đ$	styrene (%)[a]	toluene (%)[a]	AMS (%)[a,b]	acrylonitrile (%)[c]
1	11.2	8.74	36.3	1.0	0.7	21.2

[a]Calculated based on just styrene units of the copolymer
[b]Alpha-methylstyrene (AMS)
[c]Calculated based on just acrylonitrile units of the copolymer

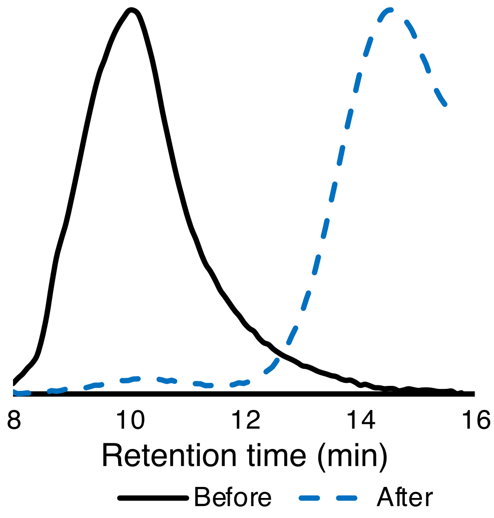

Figure S84. GPC of PS-*co*-PAN-$CB_{5.3}$ after photothermal depolymerization.

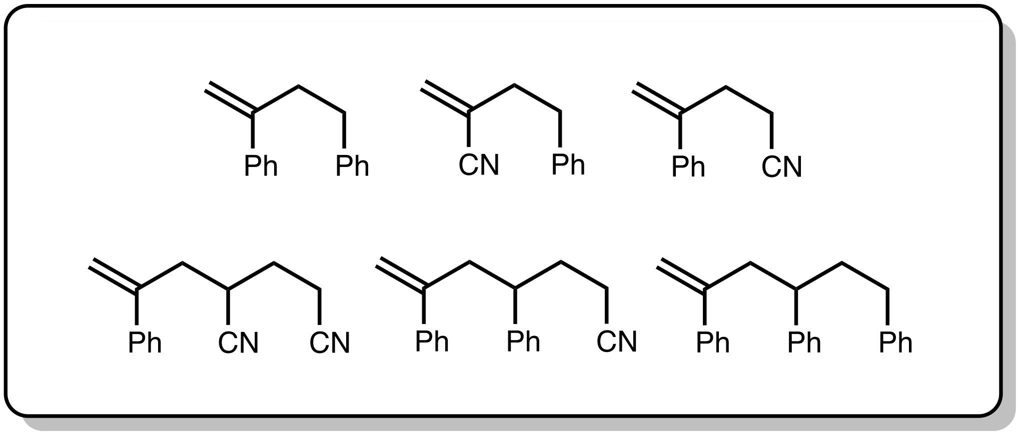

Figure S85. Plausible structures based on GC-MS and ^{1}H NMR studies.

Procedure for PS-*co*-PMA Photothermal Depolymerization

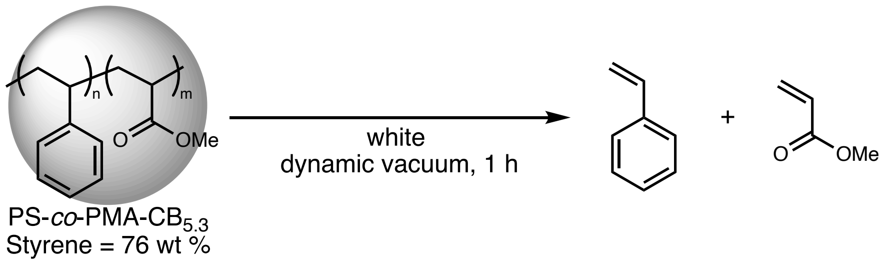

The procedure was slightly modified from the procedure for photothermal depolymerization under dynamic vacuum. PS-*co*-PMA-$CB_{5.3}$ (50 mg, carbon black = 5.3 wt %) was used, and depolymerization time was an hour. Liquid nitrogen was used instead of the dry ice acetone bath for the receiving flask. The depolymerization results were summarized, and GPC of the depolymerized samples were shown below.

Table S22. Results of photothermal depolymerization with PS-*co*-PMA-$CB_{5.3}$.

Entry	M_n (kDa)	$Đ$	styrene (%)[a]	toluene (%)[a]	AMS (%)[a,b]	MA (%)[c]
1	14.4	3.69	40.0	0.7	0.7	17.2

[a]Calculated based on just styrene units of the copolymer
[b]Alpha-methylstyrene (AMS)
[c]Calculated based on just MA units of the copolymer

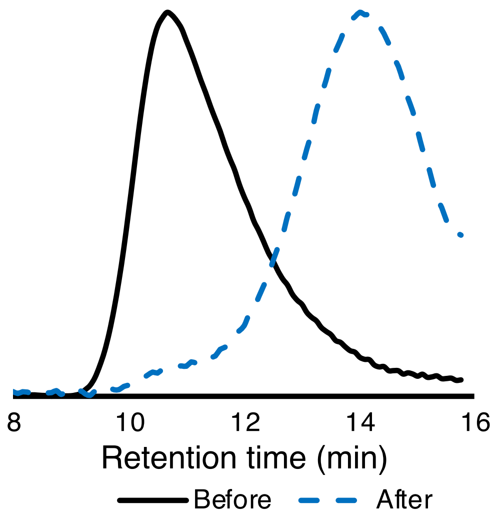

Figure S86. GPC of PS-*co*-PMA-$CB_{5.3}$ after photothermal depolymerization.

Procedure for PS-*co*-PI-CB Photothermal Depolymerization

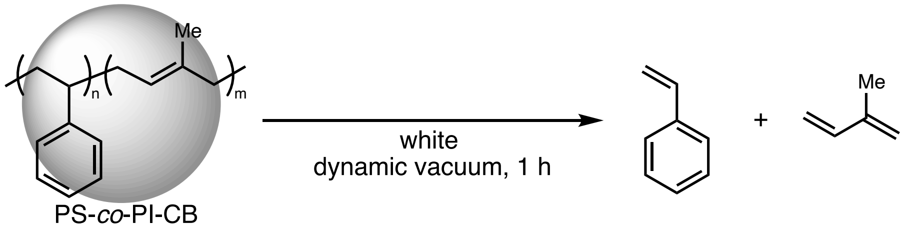

The procedure was slightly modified from the procedure for photothermal depolymerization under dynamic vacuum. PS-*co*-PI-CB (50 mg) was used, and depolymerization time was an hour. Liquid nitrogen was used instead of the dry ice acetone bath for the receiving flask. The depolymerization results were summarized, and GPC of the depolymerized samples were shown below.

Table S23. Results of photothermal depolymerization with PS-*co*-PI-CB.

Entry	Sample	M_n (kDa)	$Đ$	styrene (%)[a]	toluene (%)[a]	AMS (%)[a,b]	isoprene (%)[c]	limonene (%)[c,d]
1	PS-*co*-PI-CB$_{5.2}$ (styr 30 wt %)	1.2	2.06	14.1	0.9	0.9	4.2	2.2
2	PS-*co*-PI-CB$_{5.1}$ (styr 71 wt %)	1.2	3.05	21.1	1.1	0.7	3.0	1.2
3	hydrogenated PS-*co*-PI-CB$_{6.6}$	2.1	2.86	20.0	1.9	1.2	0	0

[a]Calculated based on just styrene units of the copolymer
[b]Alpha-methylstyrene (AMS)
[c]Calculated based on just isoprene units of the copolymer
[d]Dimerization of two isoprene

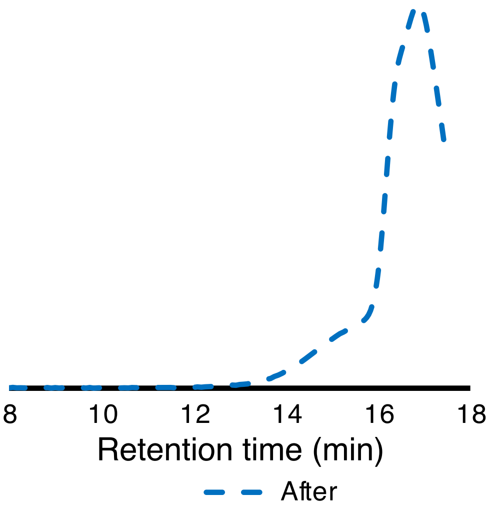

Figure S87. GPC of PS-*co*-PI-CB$_{5.2}$ (styrene 30 wt %) after photothermal depolymerization.

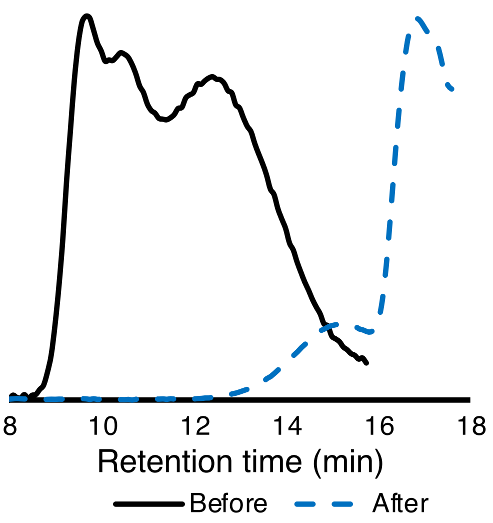

Figure S88. GPC of PS-*co*-PI-$CB_{5.1}$ (styrene 71 wt %) after photothermal depolymerization.

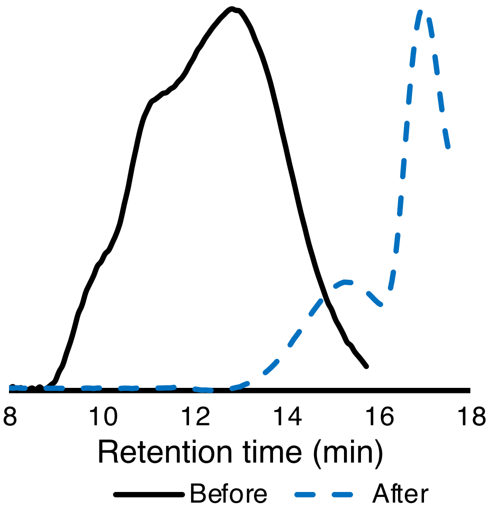

Figure S89. GPC of hydrogenated PS-co-PI-$CB_{6.6}$ after photothermal depolymerization.

Procedure for Determining the Percent Purity of PS in Post-consumer Waste PS Samples

In a 1-dram vial, ~50-100 mg of post-consumer waste PS sample were dissolved in 2 mL of $CDCl_3$. 1,3,5-Trimethoxybenzene were added as an internal standard. ^{1}H NMR spectra of all commercial PS samples were shown below. GPC were taken for soluble parts of all samples (GPC traces see Fig. S107-108), and the PS percent purity and molecular weight information below.

Table S24. Sample purity and molecular weight information of all commercial PS samples.

Entry	Sample Name	Sample Mass (mg)[a]	Standard Mass (mg)[b]	PS Purity (%)	Additives (%)[c]	M_n (kDa)	Đ
1	Black PS Foam Tray	38.5	10.1	90.1	9.9	106.3	2.53
2	Film Container	92.6	20.2	96.2	3.8	93.4	2.52
3	Sushi Box	96.8	20.2	55.6	44.4	89.0	2.33
4	Flower Pot	103.4	20.2	86.9	13.1	105.5	1.94
5	LED Container	106.6	20.2	72.4	27.6	63.7	2.61
6	Black Coffee Cup Lid	104.8	20.2	86.1	13.9	94.3	2.30
7	Cake Tray	99.7	20.2	97.1	2.9	82.2	2.50
8	3D-Printer HIPS	91.3	20.2	90.4	9.6	83.7	2.08
9	White Coffee Cup Lid	87.7	20.2	89.0	11.0	98.9	2.16
10	White PS Foam	60.2	10.1	>99	<1	102.3	2.84
11	Clear Container Lid	95.3	20.2	>99	<1	119.6	2.37
12	Clear Cup	102.8	20.2	93.8	6.2	73.0	2.60
13	Pure Polystyrene[d]	-	-	>99	0	83.7	2.41
14	Red PS Cup	115.4	20.2	81.1	<1	73.5	2.76
15	Yellow PS Foam Tray	100.1	20.2	>99	<1	119.0	2.28

[a]Dissolved overnight in $CDCl_3$. [b]1,3,5-trimethoxybenzene used as NMR standard. [c]Additives amount calculated with 100% - PS purity. [d]M_w = 192 kDa, purchased from Sigma Aldrich; assuming 100% purity.

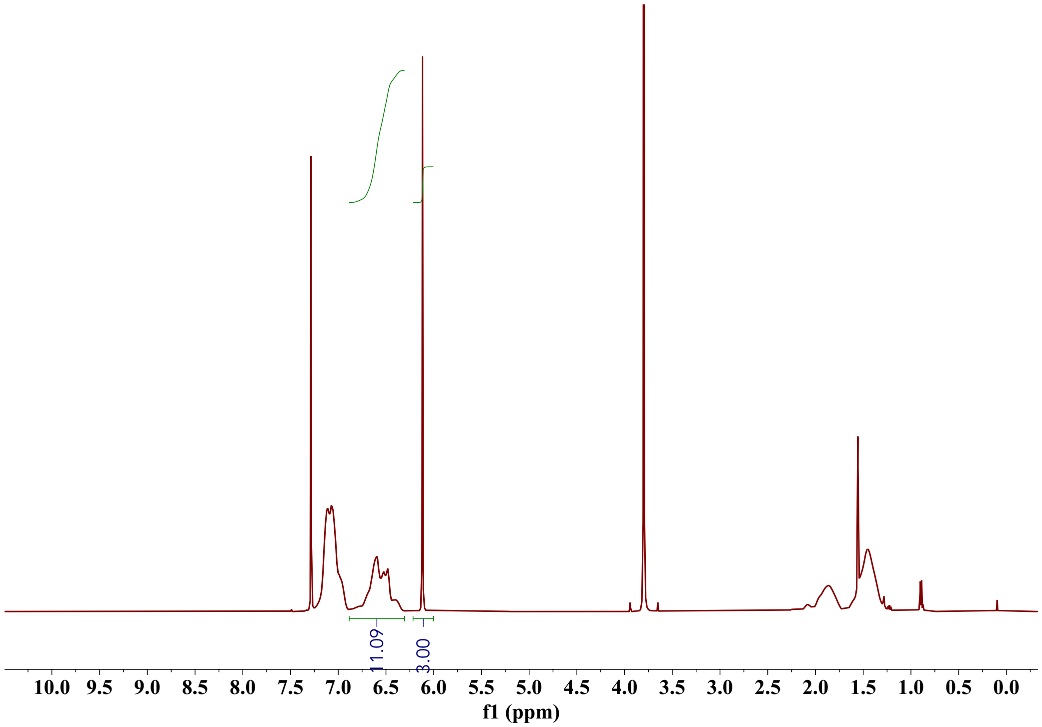

Figure S90. ^{1}H NMR of black PS foam tray.

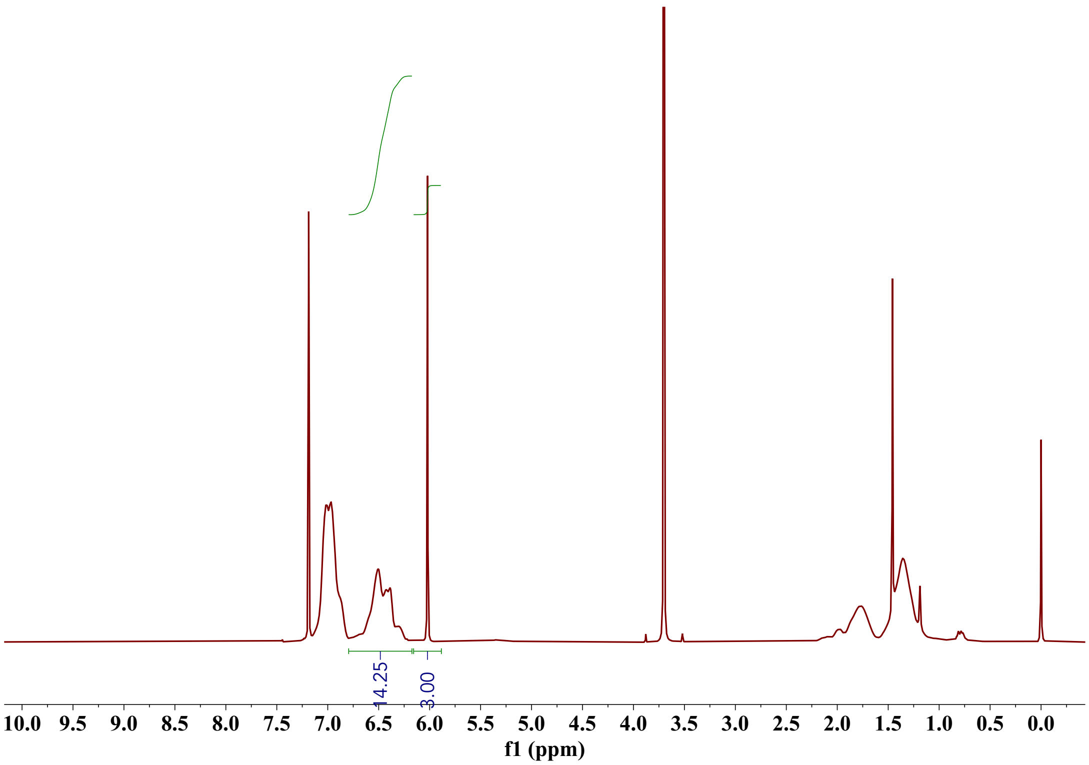

Figure S91. ^{1}H NMR of film container.

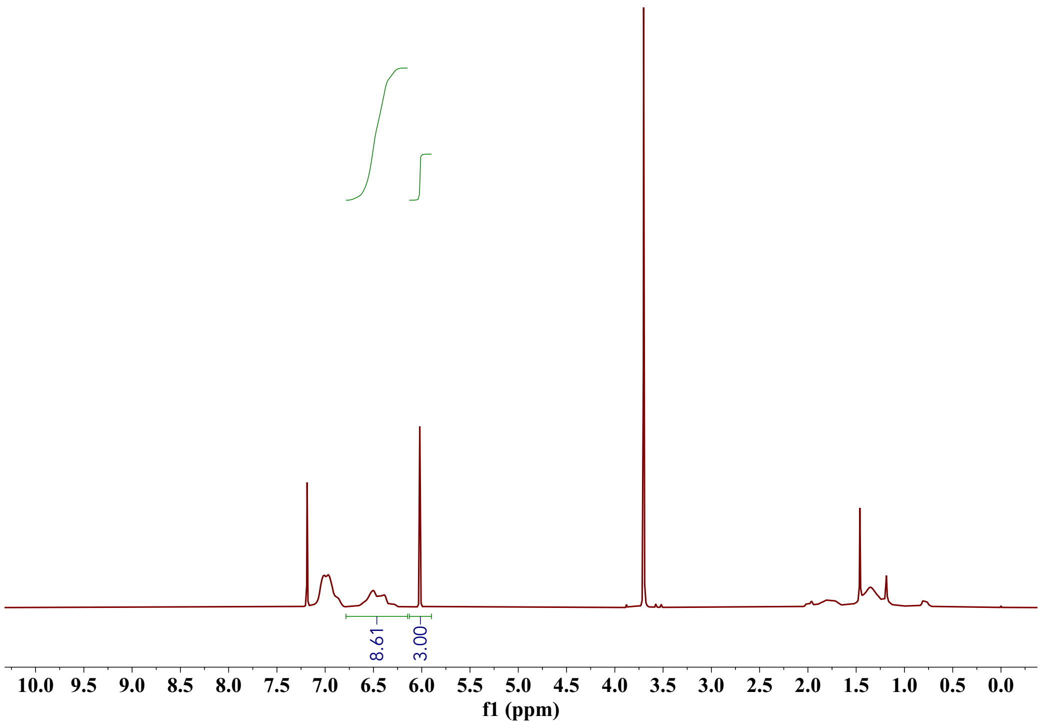

Figure S92. ^{1}H NMR of sushi box.

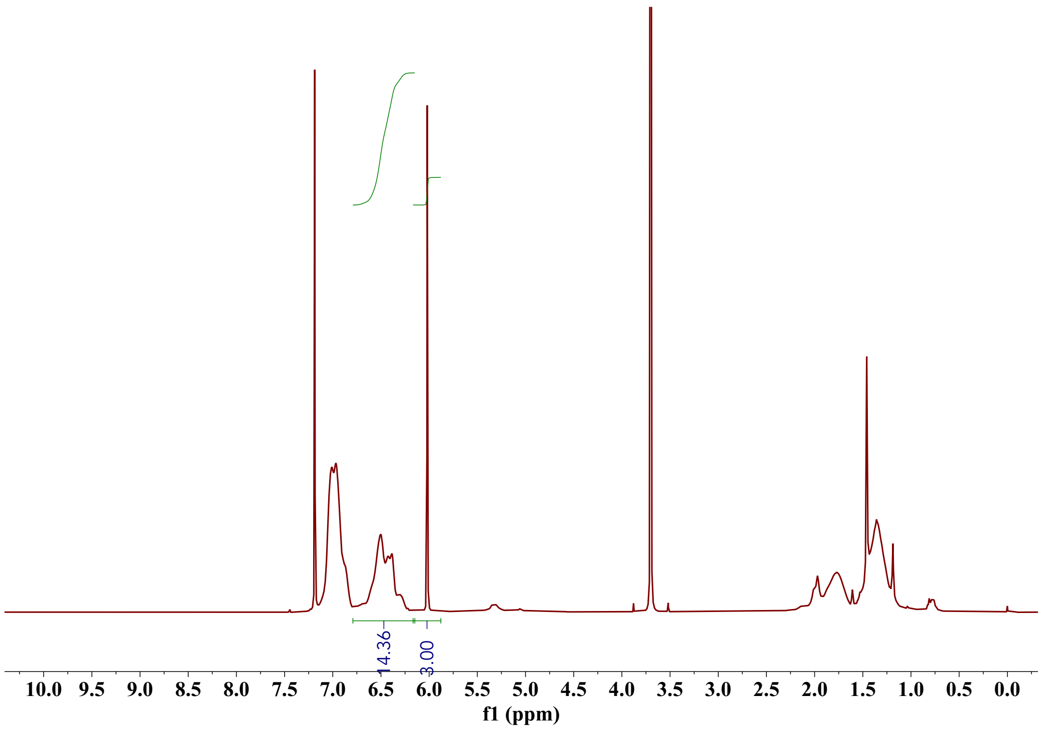

Figure S93. ^{1}H NMR of flower pot.

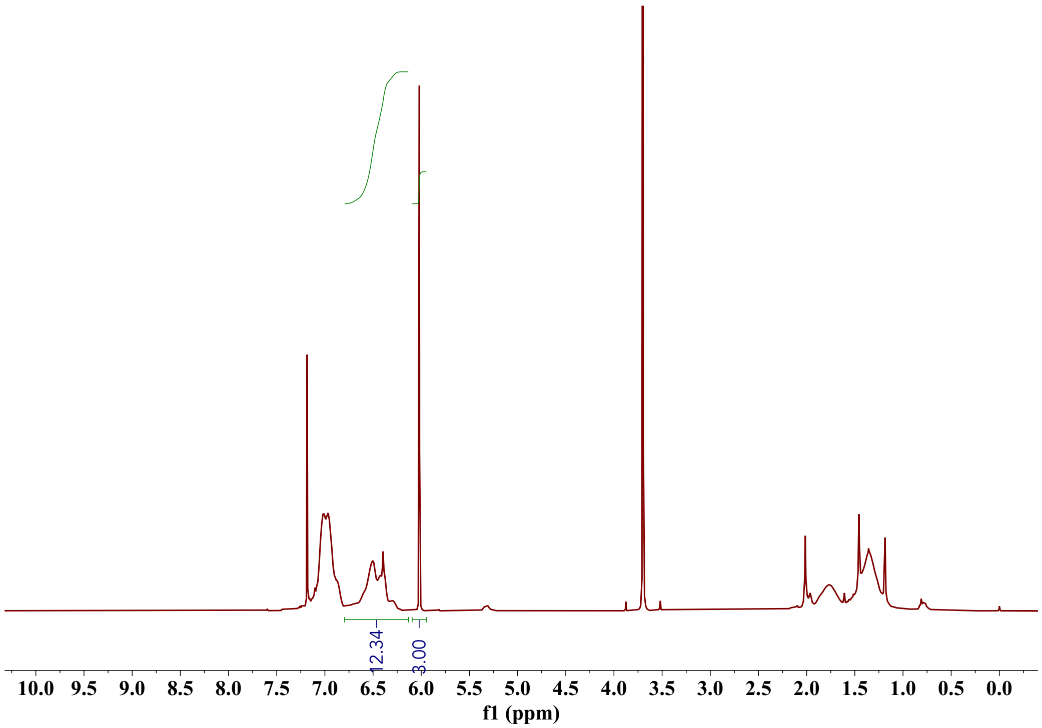

Figure S94. ^{1}H NMR of LED container.

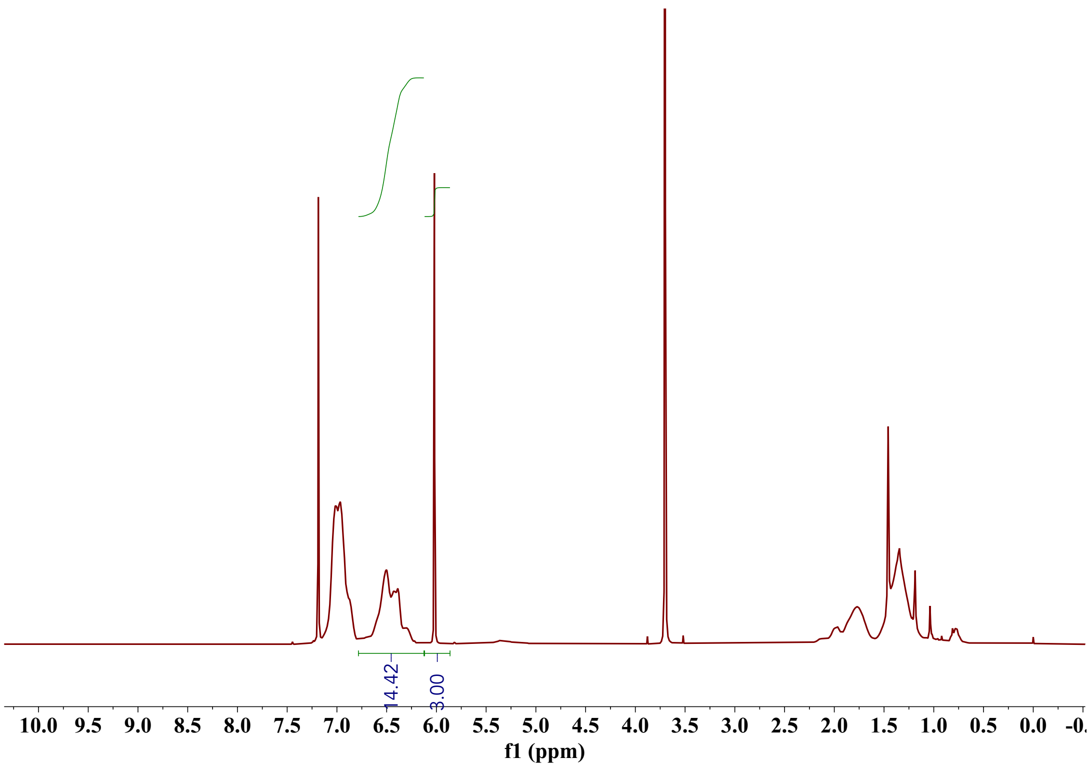

Figure S95. ^{1}H NMR of black coffee cup lid.

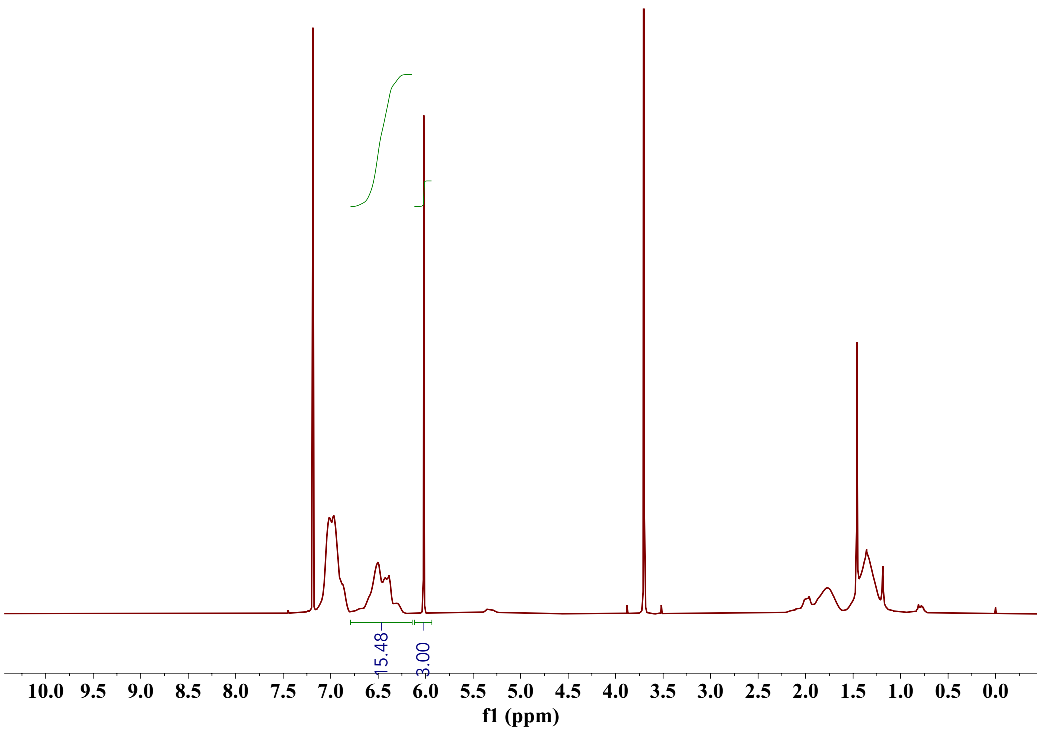

Figure S96. ^{1}H NMR of cake tray.

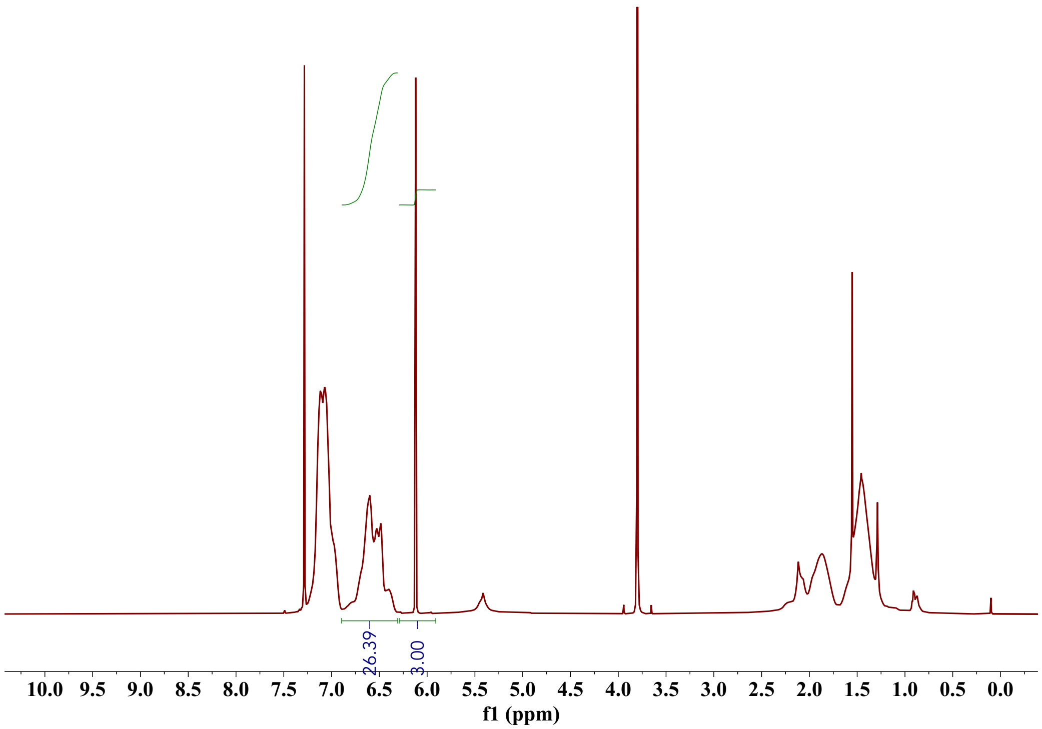

Figure S97. ^{1}H NMR of 3D-printer HIPS.

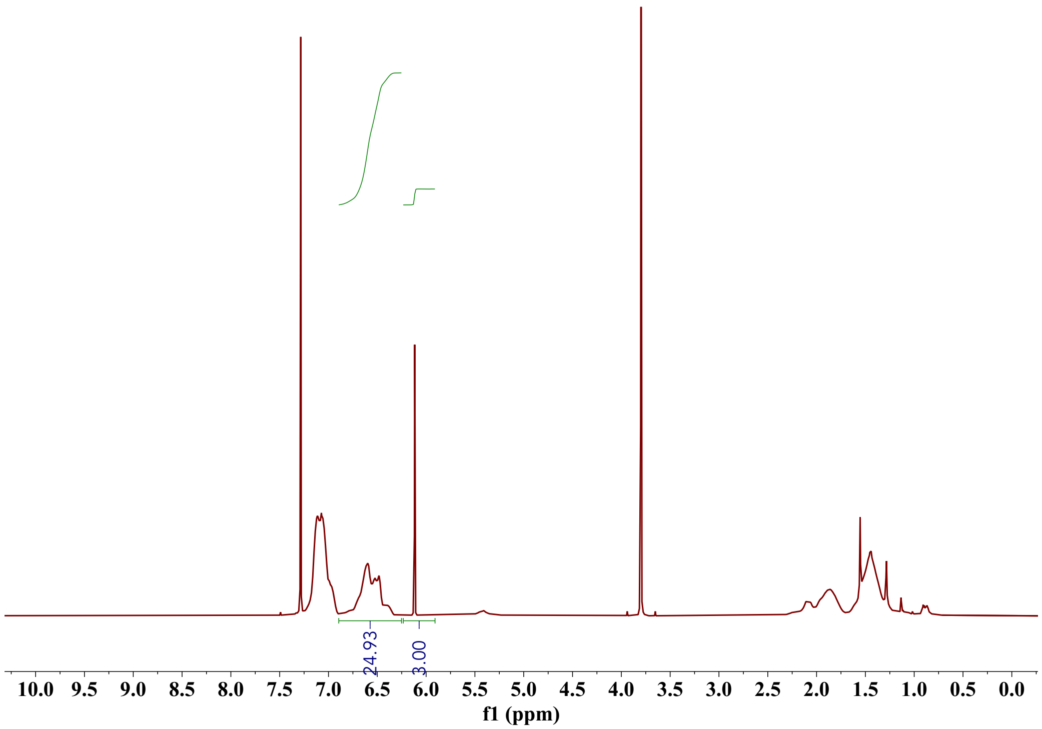

Figure S98. ^{1}H NMR of white coffee lid.

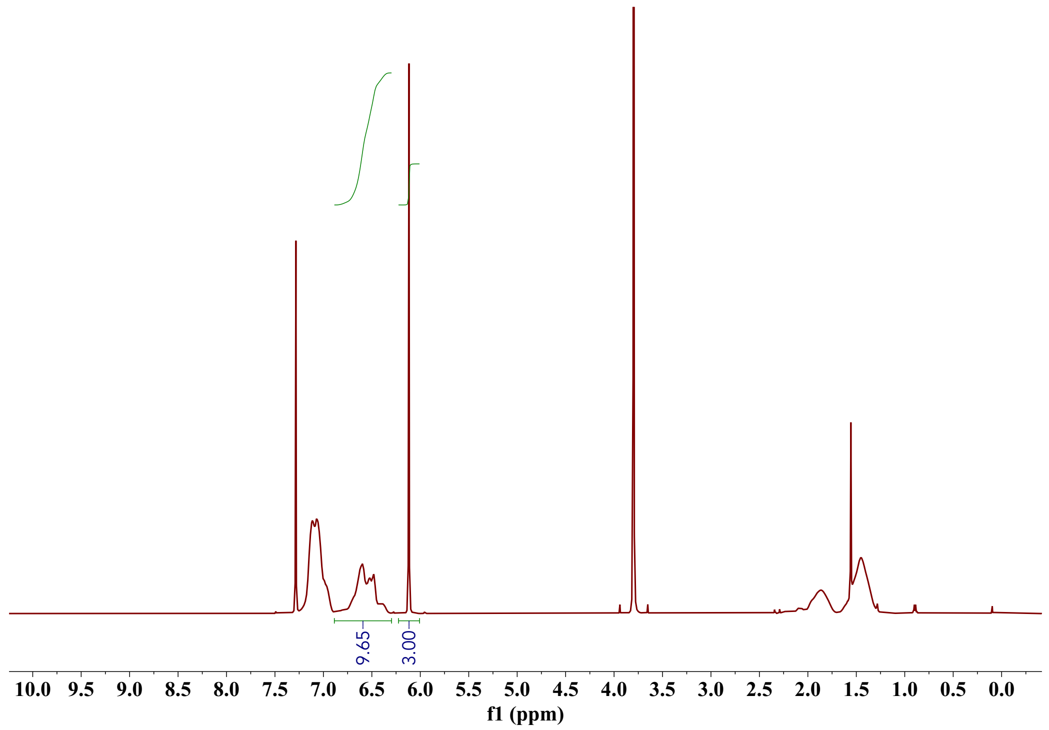

Figure S99. ^{1}H NMR of white PS foam.

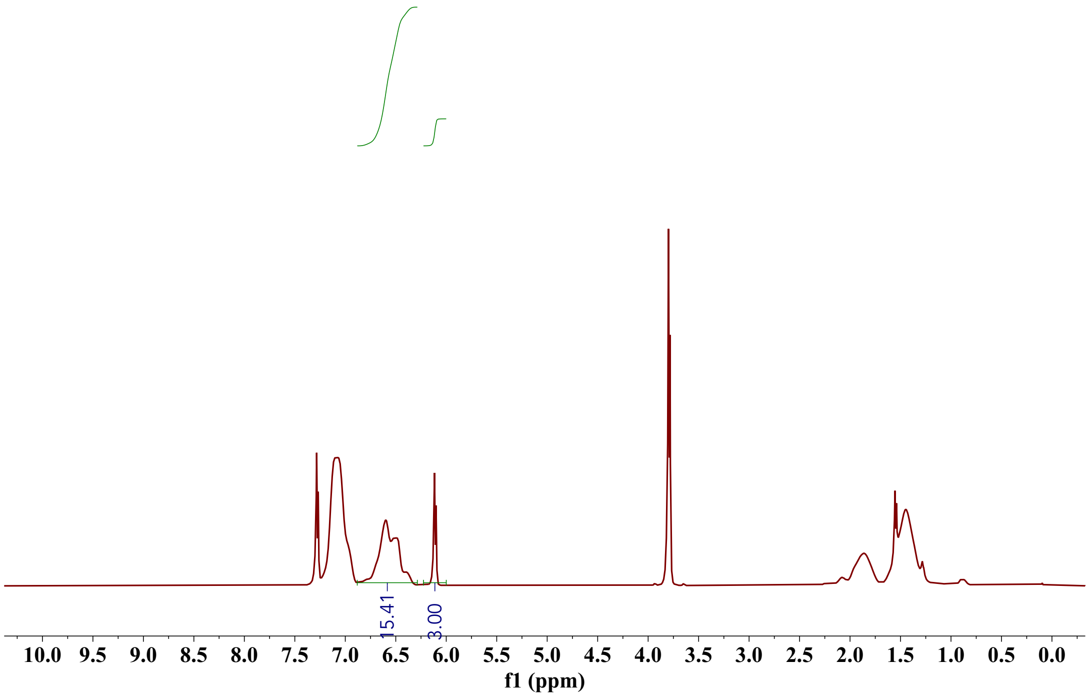

Figure S100. ^{1}H NMR of clear PS container lid.

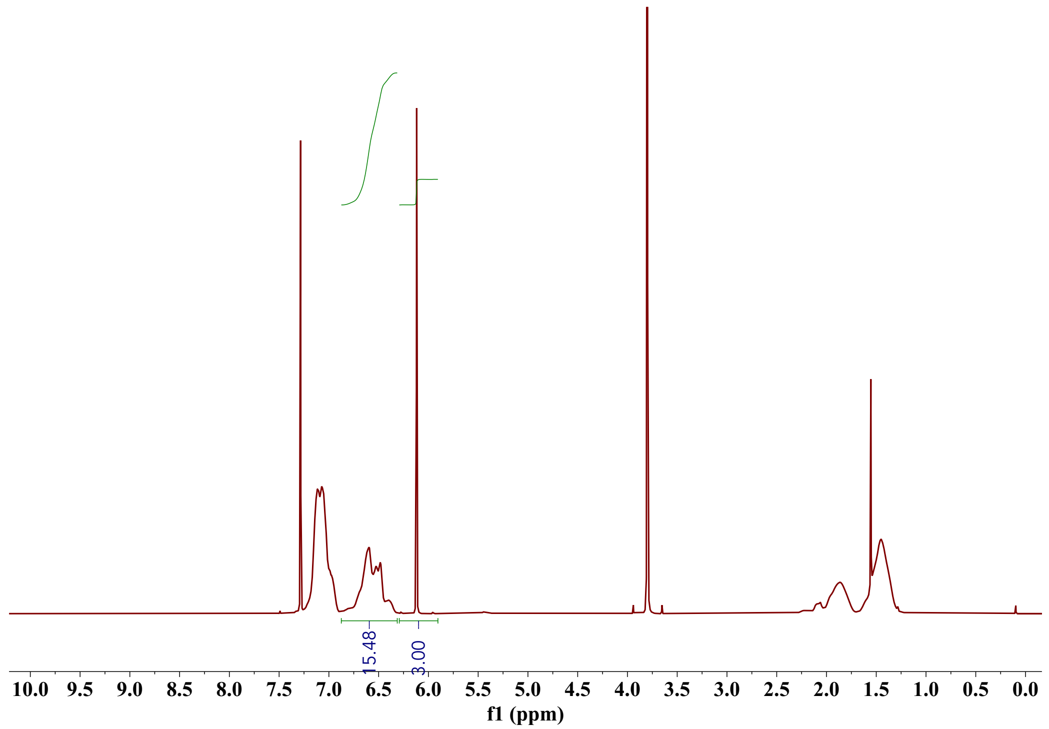

Figure S101. ^{1}H NMR of clear PS cup.

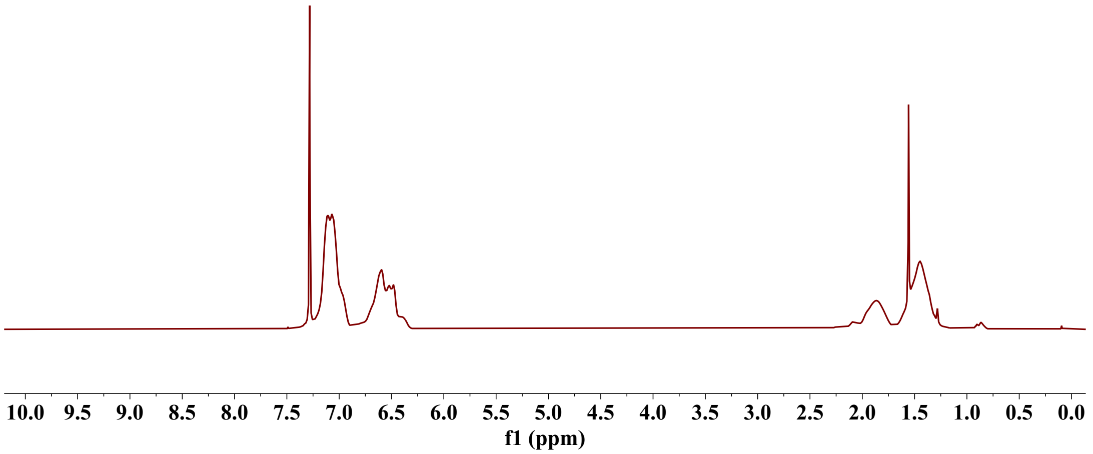

Figure S102. ^{1}H NMR of pure commercial PS from Aldrich (M_w = 192 kDa).

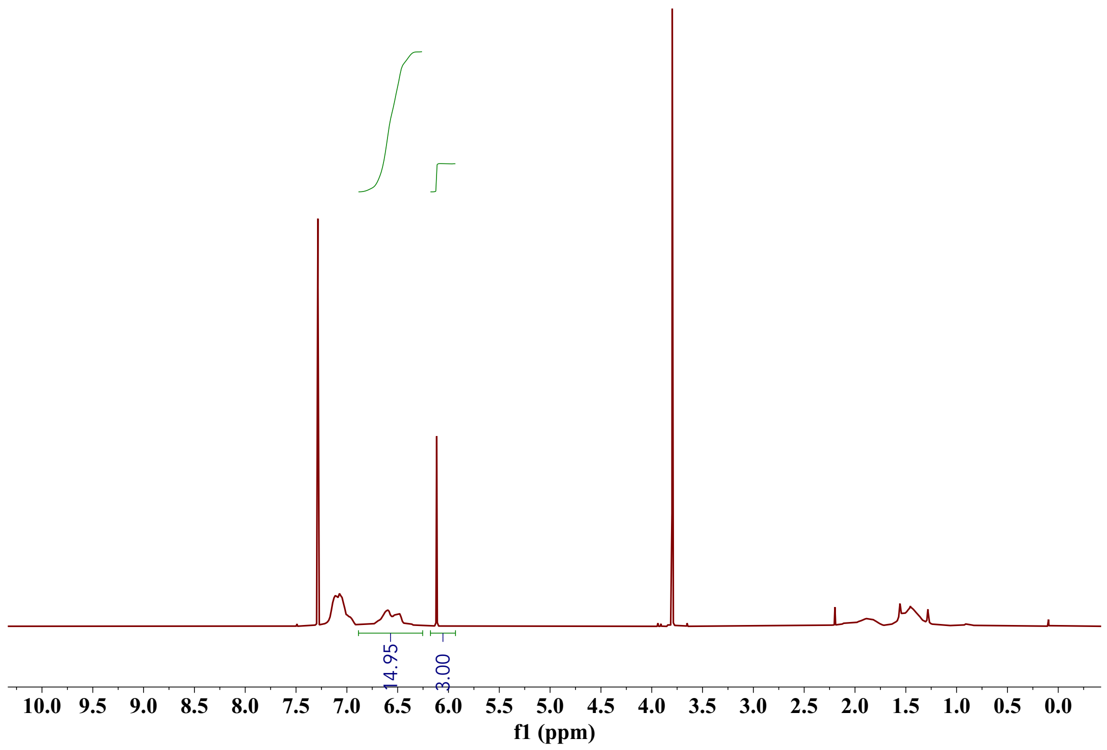

Figure S103. ^{1}H NMR of red PS cup.

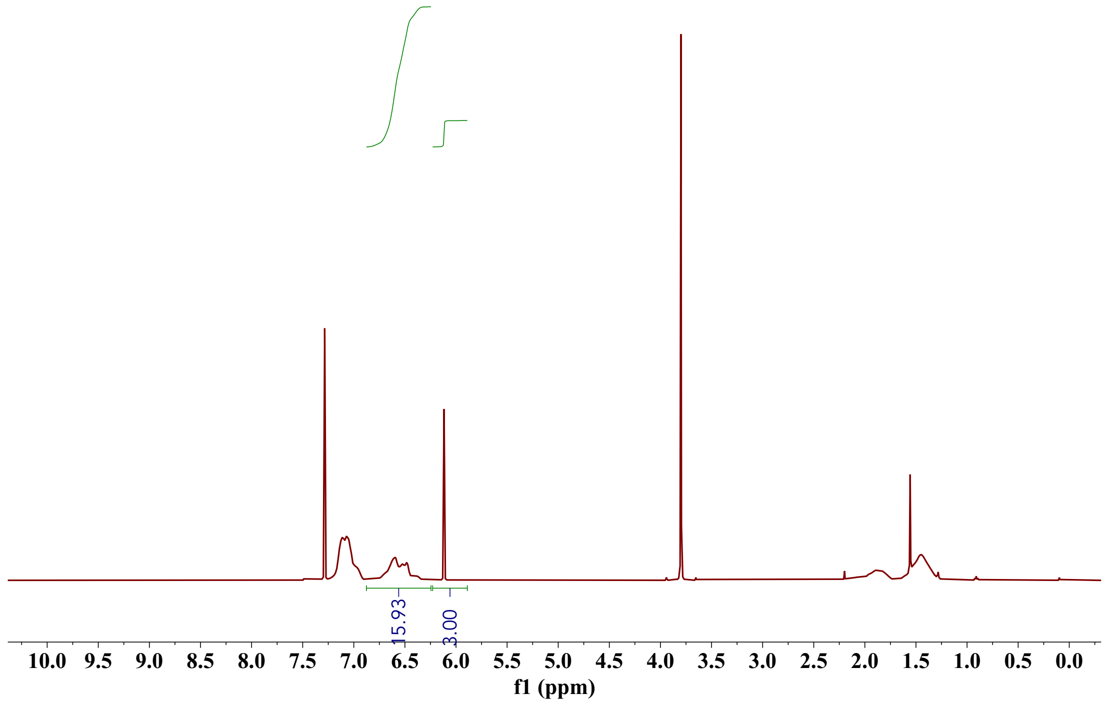

Figure S104. ^{1}H NMR of yellow PS foam tray.

Procedure for Further Characterization of Post-consumer Waste PS Samples

GPC was taken to measure the molecular weight and dispersity of each post-consumer black and non-black PS.

TGA was taken to estimate the maximum possible amount of carbon black in post-consumer black and non-black PS.

To characterize the black solids in black commercial samples, XPS analysis was done on the black PS foam tray/ film container/ black coffee cup lid. To prepare the samples, black PS was dissolved in dichloromethane/acetone, diluted until the solution was not viscous, and centrifuged at 4400 rpm for 10 minutes. The supernatant liquid was decanted and the black solids at the bottom of centrifuge tubes were collected, centrifuged with dichloromethane at 4400 rpm for 5 minutes. This "decant-centrifuge" cycle was repeated with methanol, and the resulting black solids were dried under 80 °C in a vacuum oven overnight. The powder was used directly for XPS analysis.

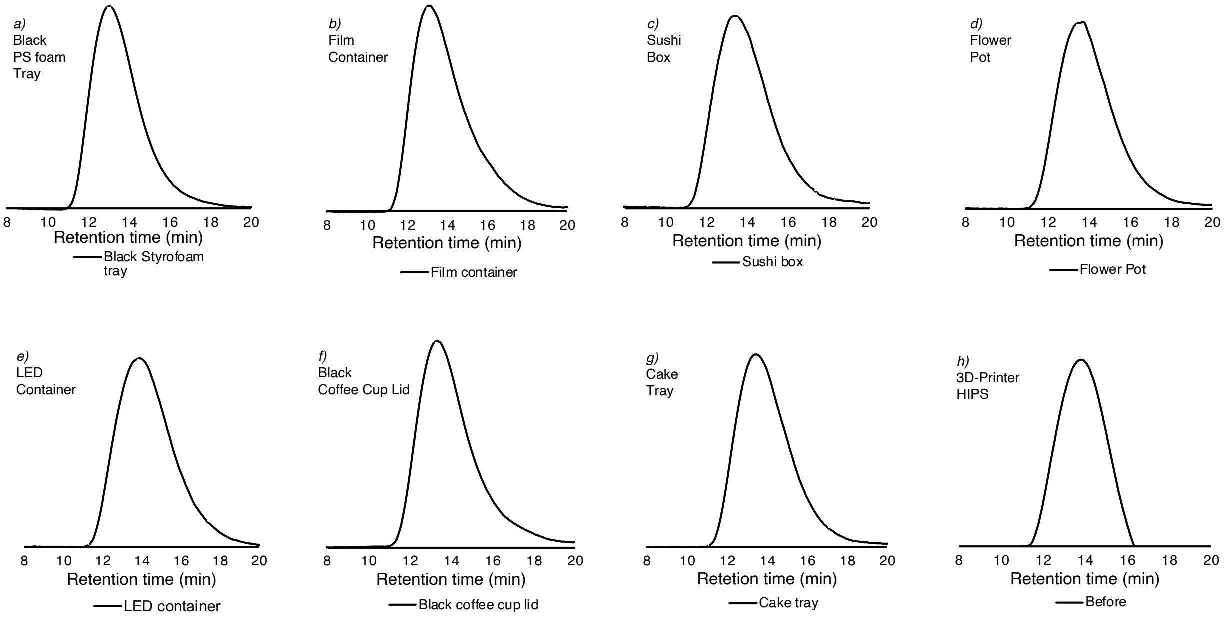

Figure S105. GPC of post-consumer waste black polystyrene samples.

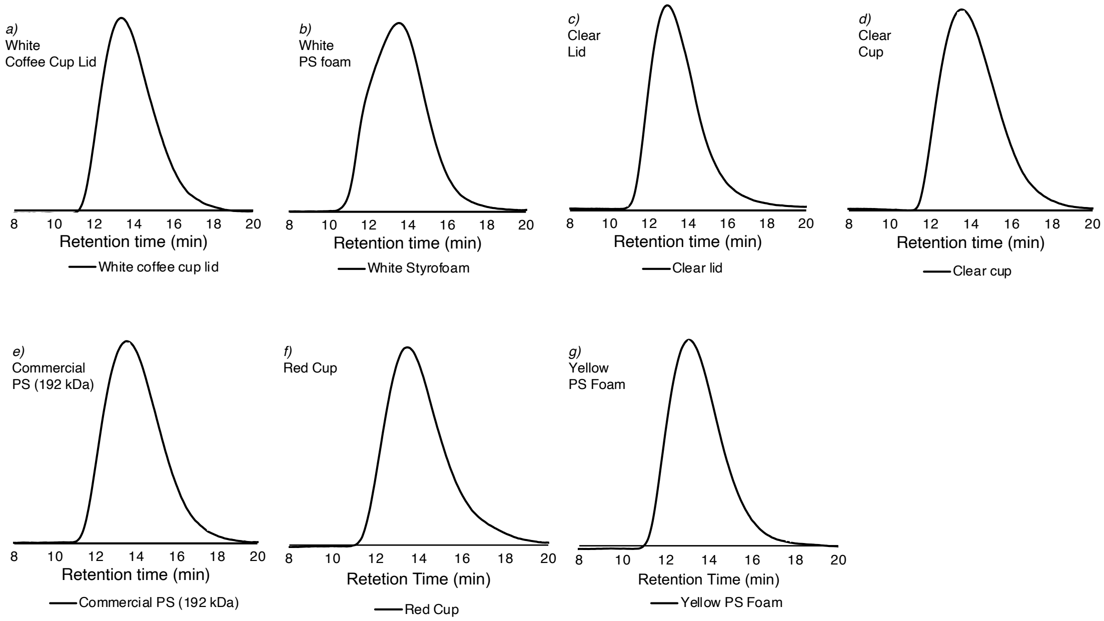

Figure S106. GPC of post-consumer waste non-black polystyrene samples.

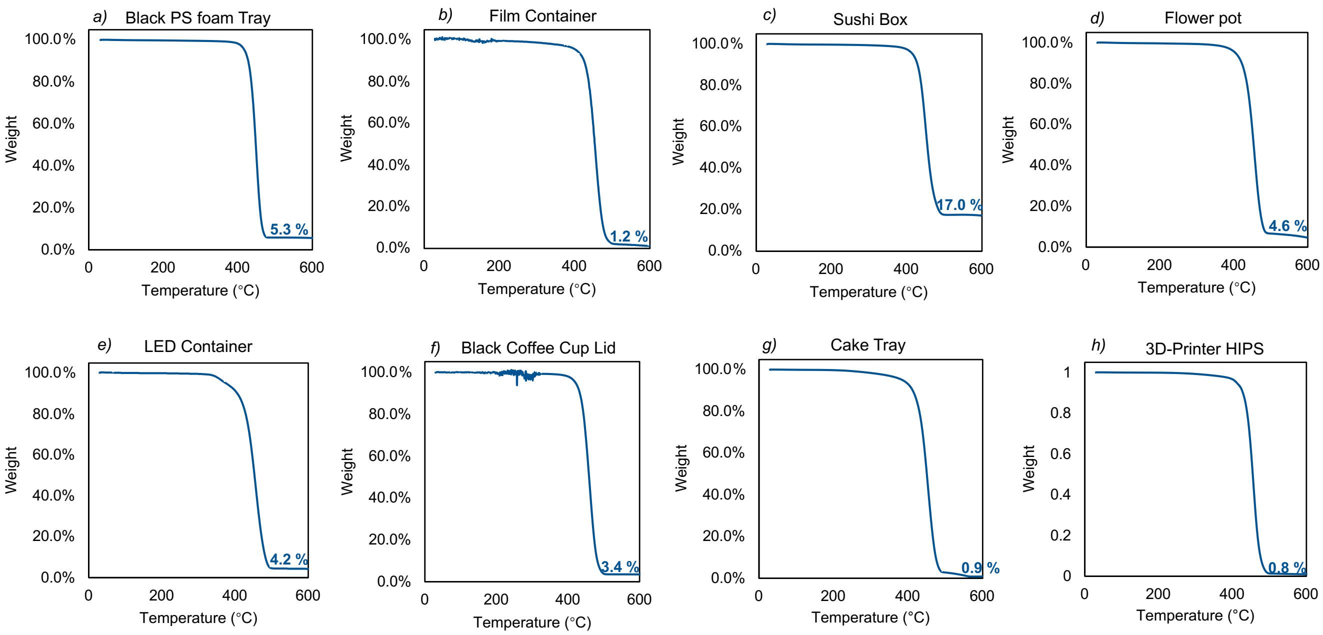

Figure S107. TGA of post-consumer waste black PS samples.

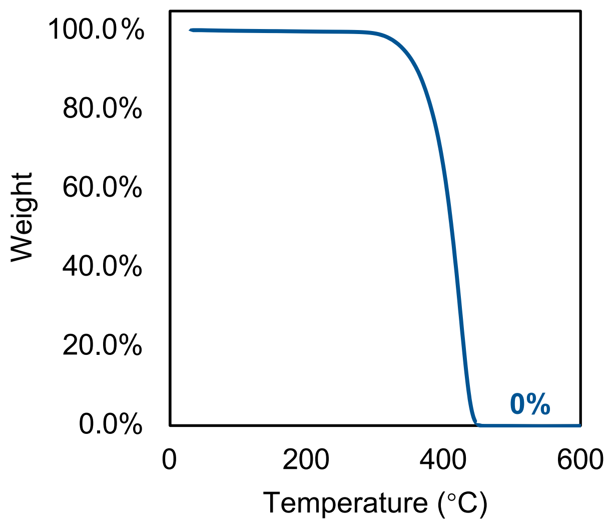

Figure S108. TGA of post-consumer waste white PS foam.

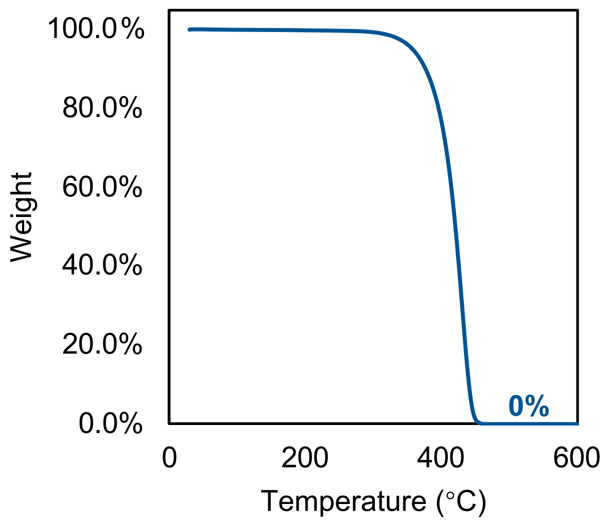

Figure S109. TGA of pure commercial PS from Aldrich (M_w = 192 kDa).

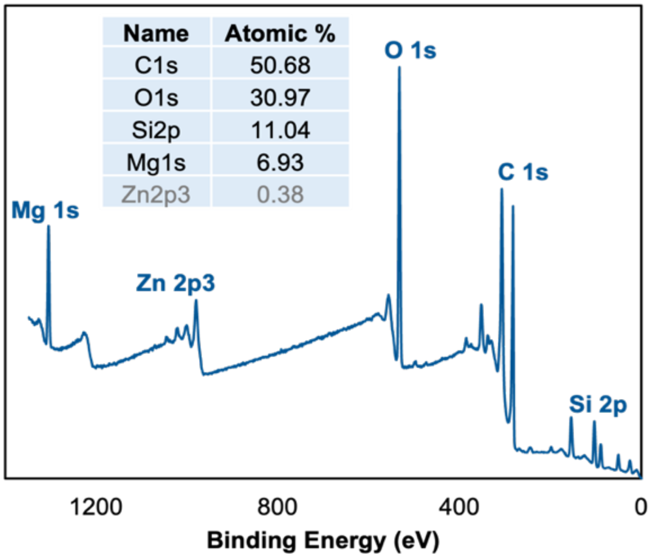

Figure S110. XPS of isolated black solid mixture from black PS foam tray.
*Magnesium oxide, magnesium hydroxide, magnesium chloride, silica, and alkali silicate are all common flame-retardants in PS.[9,10]

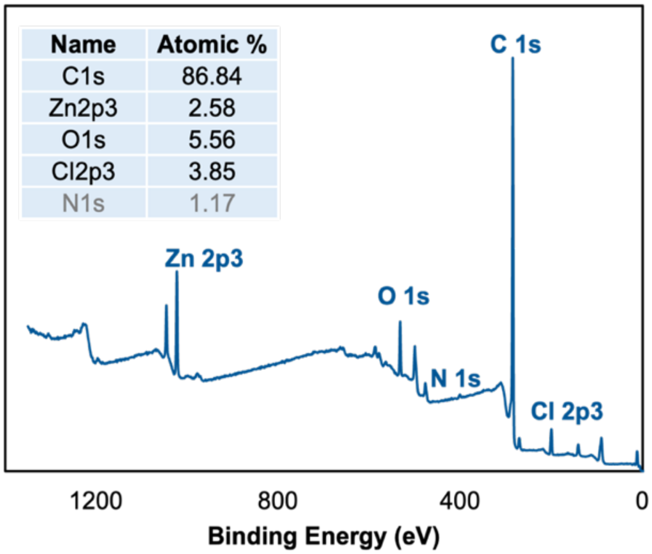

Figure S111. XPS of isolated black solid mixture from film container.
*Zinc stearate is a common additive in polystyrene.[10]

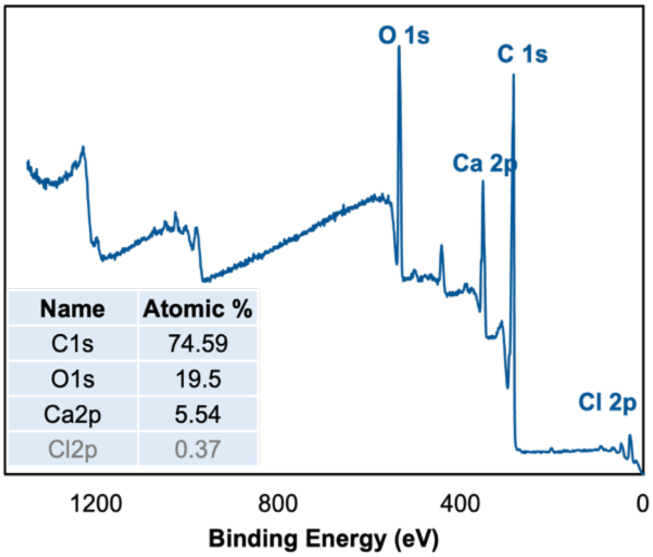

Figure S112. XPS of isolated black solid mixture from black coffee lid.
*Calcium stearate is a common additive in polystyrene.[11]

Procedure for Post-Consumer Waste Black PS Photothermal Depolymerization

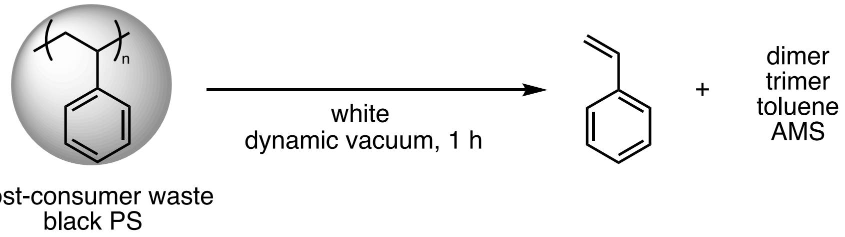

The procedure was slightly modified from the procedure for photothermal depolymerization under dynamic vacuum. All black post-consumer waste PS samples were washed with soap water, dried, and cut into about 1x1 mm squares prior to use. The black PS pieces (50 mg) was used, and depolymerization time was an hour. The depolymerization results were summarized, and GPC of the depolymerized samples were shown below.

Table S25. Results of photothermal depolymerization with post-consumer waste black PS.

Entry	**Black PS**	PS Purity (%)	Sample Mass (mg)	M_n (kDa)	$Đ$	styrene (%)	dimer (%)	trimer (%)	toluene (%)	AMS (%)[a]	leftover PS (%)	mass recov. (%)
1	PS Foam Tray	90.1	50.6	23.4	2.14	53.6	7.0	17.8	2.7	1.4	14.7	97.1
			49.6	29.2	3.21	53.5	7.0	17.0	0.9	1.4	14.7	94.6
			50.1	36.0	3.23	53.0	7.6	17.0	0.8	1.4	13.7	93.5
2	Film Container	96.2	50.3	32.1	2.37	39.9	5.4	13.8	1.5	1.0	24.3	85.8
			50.2	35.0	2.48	42.3	4.9	16.2	1.5	1.3	20.5	86.6
			50.1	59.7	2.35	40.3	5.4	13.0	0.9	1.3	23.1	83.9
3	Sushi Box	55.6	50.1	54.9	2.37	34.6	2.2	9.7	2.2	0.4	49.0	98.2
			50.1	58.8	2.33	33.3	1.6	7.3	0.9	0.4	46.3	89.8
			49.9	60.2	2.33	40.6	2.2	8.1	0.8	0.5	35.9	88.0
4	Flower Pot	86.9	50.2	42.9	2.40	32.7	3.8	11.3	1.1	0.6	38.0	87.6
			50.4	44.8	2.57	26.3	2.7	10.5	0.9	0.6	41.6	82.6
			49.9	47.8	2.53	27.4	2.7	9.7	0.7	0.6	46.1	87.2
5	LED Container	72.4	50.0	29.5	1.98	36.7	2.7	10.5	1.0	1.0	36.7	88.6
			49.9	29.1	1.82	40.0	2.2	10.5	1.3	1.4	29.9	85.8
			50.1	30.6	1.76	44.5	2.7	13.0	1.6	1.7	27.1	90.6
6	Coffee Cup Lid	86.1	50.0	43.5	2.57	32.9	3.2	10.5	1.2	0.9	42.8	91.4
			50.0	38.1	2.58	34.9	4.3	13.0	1.4	0.6	33.5	87.6
			50.1	44.4	2.65	37.2	3.8	13.8	0.8	0.9	30.6	87.0
7	Cake Tray	97.1	50.0	43.4	2.48	36.4	4.9	14.6	0.9	0.9	32.4	89.0
			49.6	52.5	2.60	39.0	5.9	6.5	1.0	1.0	36.5	88.9
			50.4	51.2	2.55	38.6	4.3	12.1	1.0	1.0	32.3	88.5
8	3D-Printer HIPS	90.4	50.2	34.5	2.65	38.6	5.0	15.7	1.5	1.1	29.7	91.6
			50.4	33.5	2.51	34.9	4.4	14.0	1.2	0.8	36.3	91.6
			50.4	31.6	2.50	37.1	4.9	14.0	1.4	0.8	31.3	89.6

All % with regard to styrene repeating units.
[a]Alpha-methylstyrene (AMS)

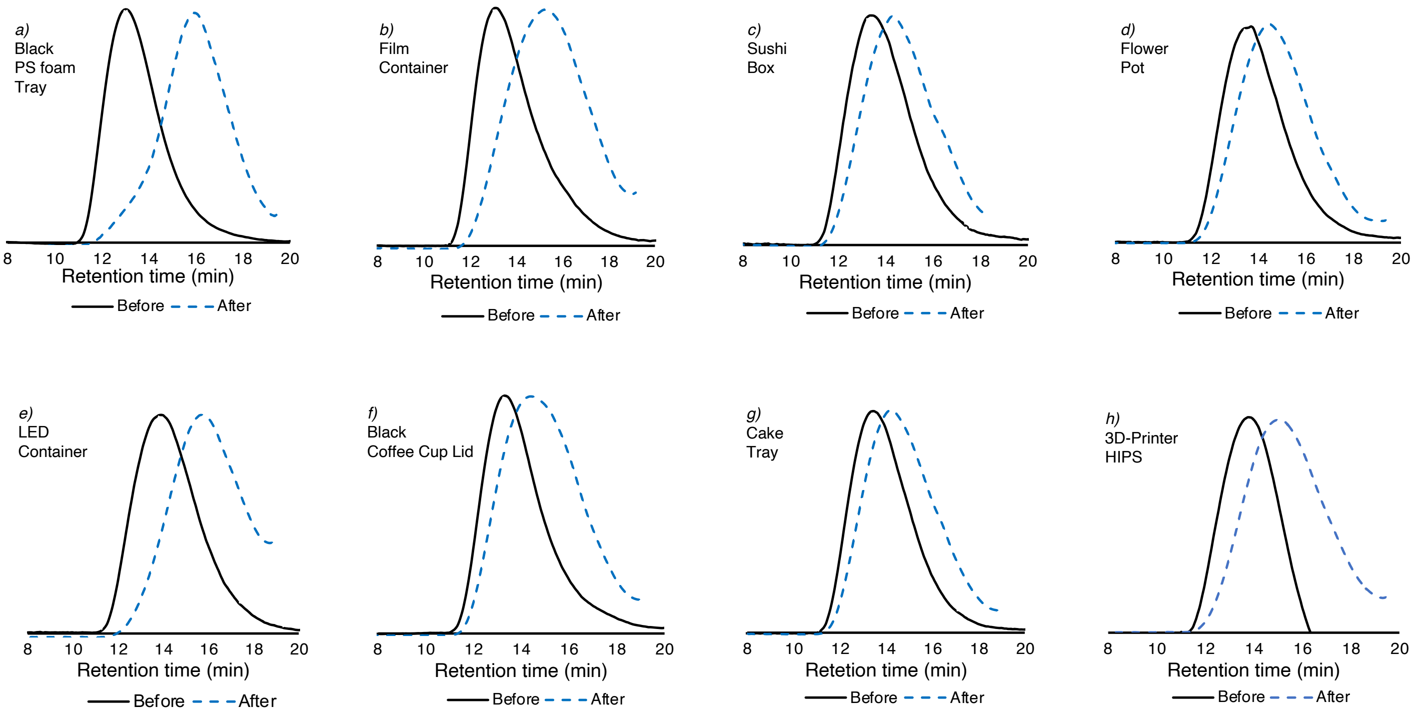

Figure S113. GPC of post-consumer waste black PS after photothermal depolymerization.

Procedure for Post-consumer Waste Non-Black PS Photothermal Depolymerization

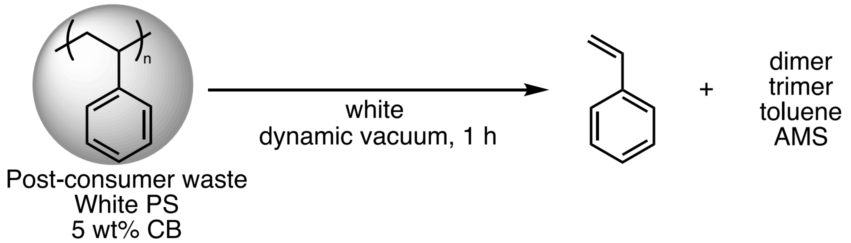

The procedure was slightly modified from the procedure for photothermal depolymerization of post-consumer waste black PS. All non-black (white or transparent) post-consumer waste PS samples were pressed using Dabpress heat press (12-TON 4x7'') under 177 °C with CB to afford 5 wt % CB in PS (950 mg non-black PS with 50 mg CB). The resulting PS-CB films were cut into 1 mm (length) x 1 mm(width) x 0.27 mm (height) pieces prior to use. The height used in the experiment were calculated by the volume of PS-CB film divided by the area of the film:

(1.05 g PS-CB film / 1.04 g/cm^3 PS density) / (π(6.9 cm/2)^2) = 0.27 mm.

The CB-added film pieces (50 mg) was used, and depolymerization time was an hour. The depolymerization results were summarized, and GPC of the depolymerized samples were shown below.

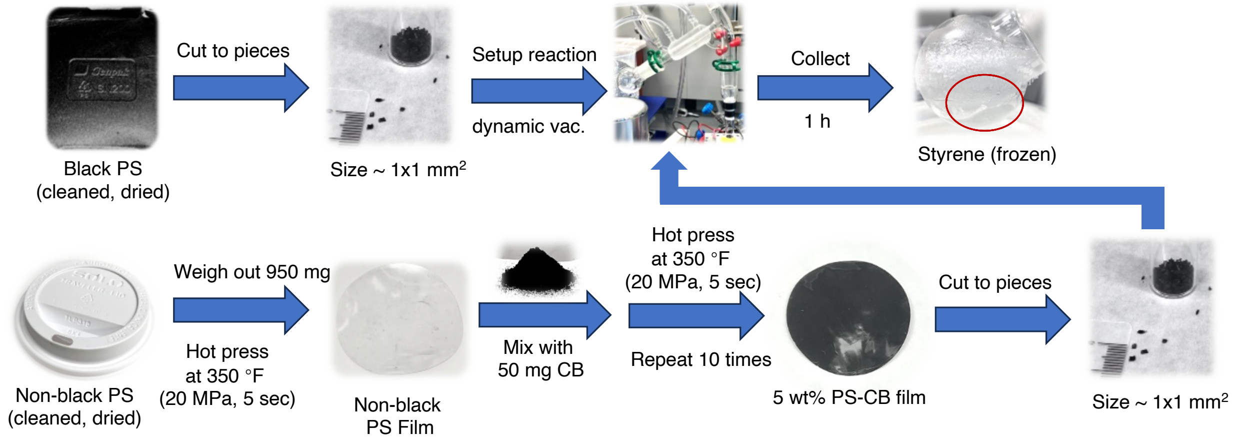

Figure S114. Visual representation of each setup for recycling post-consumer waste PS.

Table S26. Results of photothermal depolymerization with post-consumer waste non-black PS.

Entry	non-Black PS	PS Purity (%)	Sample Mass (mg)	M_n (kDa)	$Đ$	styrene (%)	dimer (%)	trimer (%)	toluene (%)	AMS (%)[a]	leftover PS (%)	mass recov. (%)
1	white coffee cup lid	89.0	50.2	39.4	2.50	28.9	3.2	8.9	0.9	0.5	56.8	>99
			50.5	45.8	2.66	28.5	3.2	7.3	0.8	0.5	59.1	>99
			50.4	41.2	2.61	28.5	3.2	9.7	0.9	0.5	49.4	92.3
2	white PS foam	>99	50.4	26.3	2.00	51.3	9.2	19.4	0.7	0.3	16.0	96.9
			50.2	25.9	1.92	50.4	9.8	18.6	0.7	0.3	20.8	>99
			50.3	26.5	2.00	51.1	8.6	19.4	0.7	0.3	19.8	>99
3	clear lid	>99	50.0	27.7	2.36	51.1	4.0	19.4	1.0	0.5	17.7	97.9
			50.1	25.1	2.00	49.5	4.3	17.0	0.9	0.5	20.1	96.6
			50.4	27.9	2.20	51.8	4.0	17.8	1.0	0.5	13.5	92.7
4	clear cup	93.8	50.5	25.5	1.95	54.8	9.2	20.2	0.6	0.5	19.4	>99
			49.9	27.9	2.17	53.0	9.2	18.6	0.8	0.3	15.1	96.9
			50.1	28.2	2.03	53.9	8.6	19.4	0.7	0.3	17.9	>99

All % with regard to styrene repeating units.
[a]Alpha-methylstyrene (AMS)

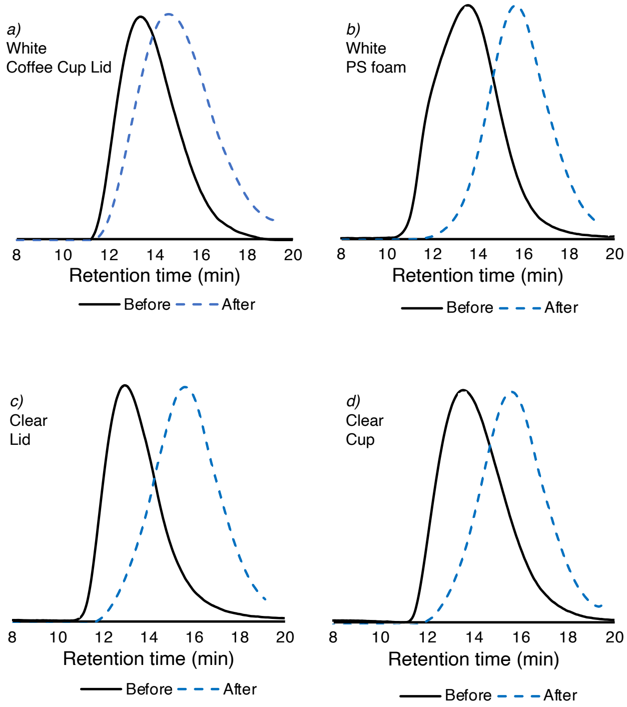

Figure S115. GPC of post-consumer waste non-black PS after photothermal depolymerization.

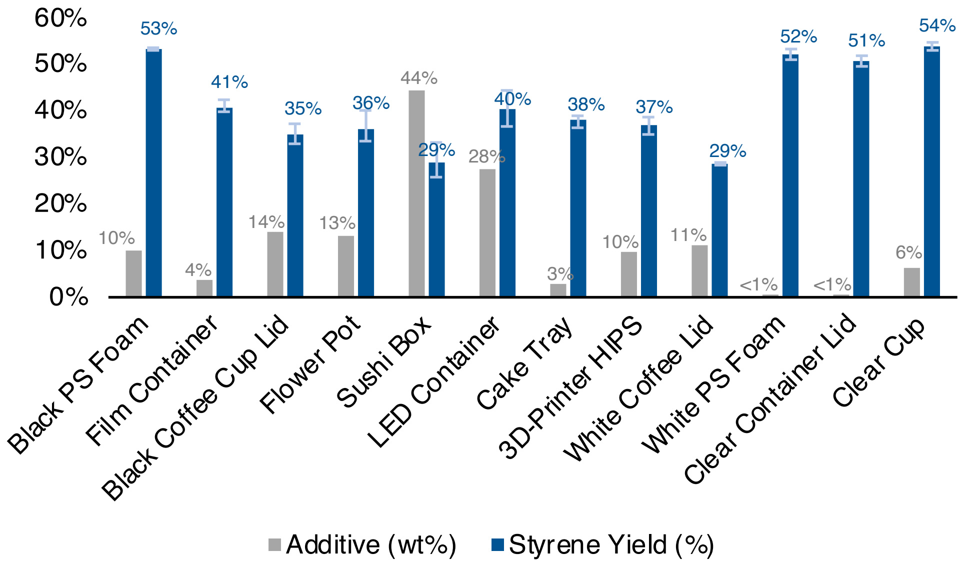

Figure S116. Bar chart of additives wt % and styrene yield of post-consumer waste PS. *Additive is defined as the non-PS/non-thermoplastic materials inherent to the plastic samples.

Procedure for Time Course Study for Commercial PS Photothermal Depolymerization

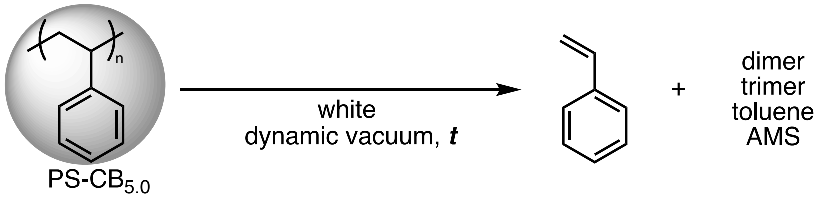

The procedure was slightly modified from the procedure for photothermal depolymerization under dynamic vacuum. Commercial PS pellets (M_w = 192 kDa, Sigma Aldrich) were powdered using a mini electronic coffee grinder (Jinyi, 110 V, 30 min), weighed out (950 mg), and ground with CB (50 mg) to an even mixture via mortar and pestle, denoted as PS-CB$_{5.0}$. Bulk temperature of the reaction vial was monitored via a K-type stainless steel thermocouple (Gain Express, temperature range -50 °C - 700 °C, resolution 0.1 °C) over an hour reaction time period.

Table S27. Results of commercial PS-CB$_{5.0}$ time course study for photothermal depolymerization.

Entry	***t*** (min)	M_n (kDa)	*Đ*	mass recov. (%)
1	5	29.2	2.32	97.5
2	10	28.3	2.18	95.7
3	15	26.7	2.17	95.8
4	30	26.2	2.01	98.0
5	60	26.0	2.32	95.0

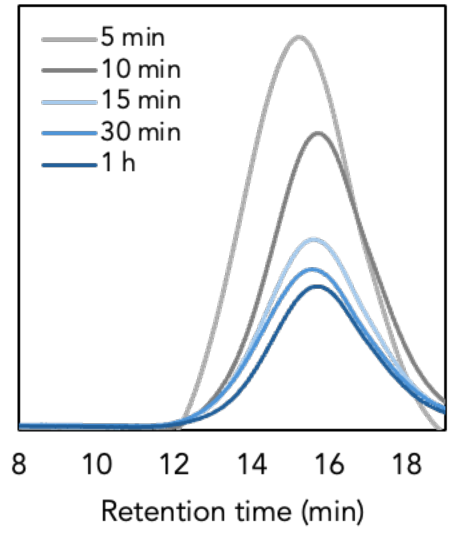

Figure S117. GPC of un-normalized commercial PS-CB$_{5.0}$ time course.

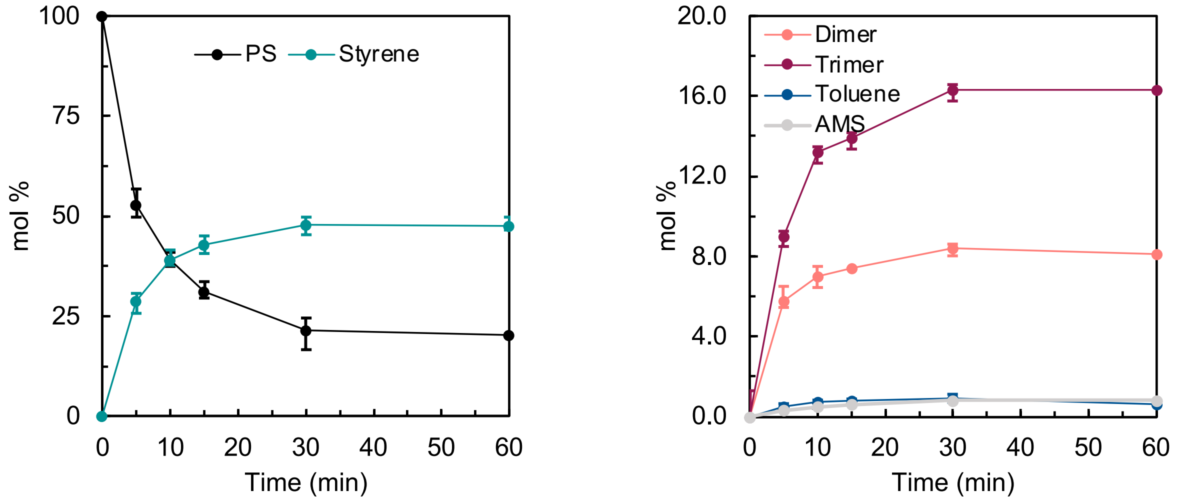

Figure S118. Scatter plot for the results of kinetic study for commercial PS-CB$_{5.0}$ photothermal depolymerization.

*We observed a trend similar to that of the lab made PS-CB samples, achieving 48% styrene in 30 min. This indicates that our system would likely be compatible with previously existing industrial depolymerization conditions.

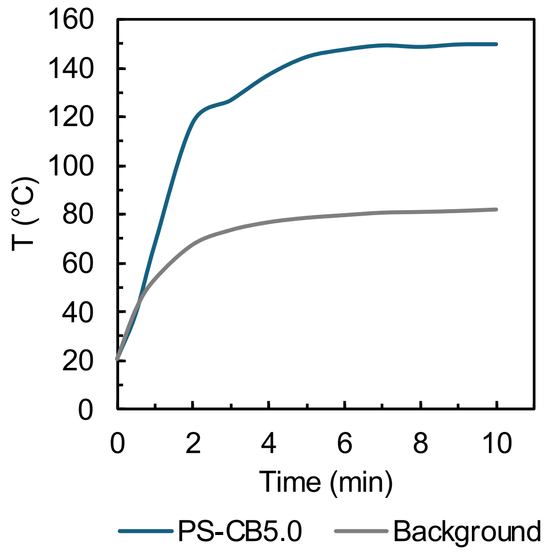

Figure S119. Bulk temperature of commercial PS-CB$_{5.0}$ photothermal depolymerization in 1 hour measured using thermocouple.

*The PS-CB$_{5.0}$ bulk temperature was measured with the stainless steel end of the thermocouple wrapped in foil and inserted in the reaction vial. The background temperature was measured without any PS-CB composite inside. The temperature stabilized around 150 °C, much lower than degradation temperature of PS, which indicates low bulk temperature of the photothermal system.

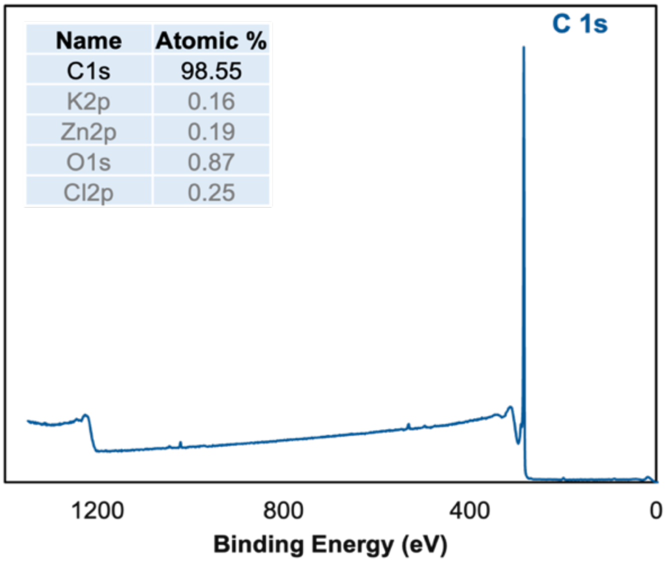

Figure S120. XPS of carbon black (from Alfa Aesar) isolated after photothermal reaction on PS-$CB_{5.0}$.
*After photothermal reactions, the left-over solids in reaction vials (including carbon black) was centrifuged at 4400 rpm in 15 mL dichloromethane for 30 minutes, and black solids were collected at the bottom of the centrifuge tubes. The collected carbon black was centrifuged with 15 mL dichloromethane (10 minutes, 4400 rpm) followed by 15 mL methanol (10 minutes, 4400 rpm) and dried under 80 °C overnight.

Procedure for Contaminated Black PS foam Photothermal Depolymerization

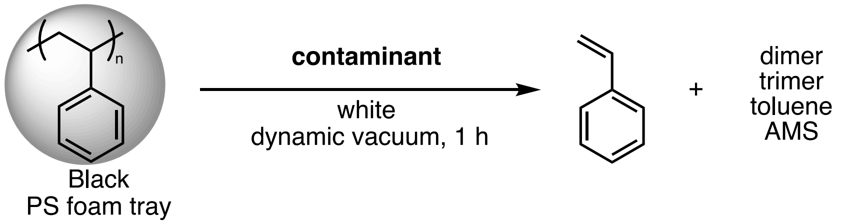

The procedure was slightly modified from the procedure for photothermal depolymerization of post-consumer waste black PS. 10 mg (20 wt %) or 50 mg (100 wt %) of contaminant (canola oil / sugar / soy sauce / orange juice) were added into 50 mg of black PS foam pieces, and depolymerization time was an hour. The depolymerization results were summarized, and GPC of the depolymerized samples were shown below.

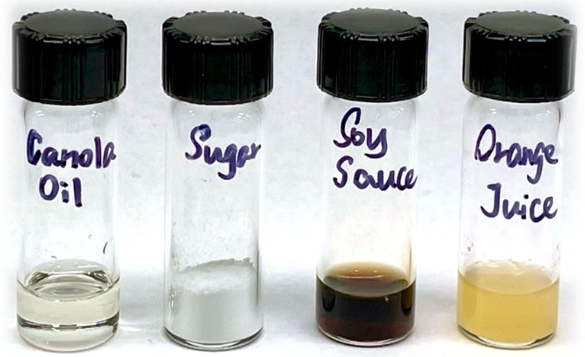

Figure S121. Picture for food contaminants used (canola oil/ sugar/ soy sauce/ orange juice).

Table S28. Results of 20 wt % contaminants study on black PS foam tray photothermal depolymerization.

Entry	**Contaminant (20 wt %)**	M_n (kDa)	*Đ*	styrene (%)	dimer (%)	trimer (%)	toluene (%)	AMS (%)[a]	leftover PS (%)	mass recov. (%)
1	canola oil	37.8	2.93	46.1	5.5	14.9	0.9	0.8	29.2	97.5
2	sugar	43.3	3.42	52.9	6.6	16.6	1.1	1.4	18.7	97.3
3	soy sauce	56.4	3.50	45.6	7.2	14.9	0.7	1.1	29.7	>99
4	orange juice	38.3	3.95	37.0	8.8	8.3	0.2	0.6	33.2	88.1

All % with regard to styrene repeating units.
[a]Alpha-methylstyrene (AMS)

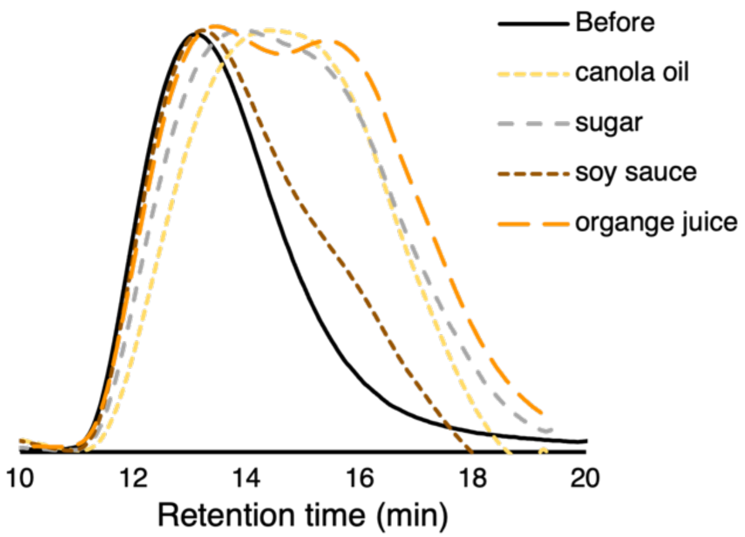

Figure S122. GPC of 20 wt % contaminants before and after photothermal depolymerization.

Table S29. Results of 100 wt % contaminants study on black PS foam tray photothermal depolymerization.

Entry	**Contaminant (100 wt %)**	M_n (kDa)	*Đ*	styrene (%)	dimer (%)	trimer (%)	toluene (%)	AMS (%)[a]	leftover PS (%)	mass recov. (%)
1	canola oil	67.8	2.61	43.4	4.4	15.8	1.1	1.1	31.7	97.5
2	sugar	41.7	2.94	45.3	5.6	15.0	0.9	1.4	24.4	92.6
3	soy sauce	58.2	3.09	23.3	2.8	9.2	0.4	0.6	68.8	>99
4	orange juice	63.3	3.02	30.5	4.4	11.7	0.5	0.8	51.6	>99

All % with regard to styrene repeating units.
[a]Alpha-methylstyrene (AMS)

Table S30. Results of *L*-ascorbic acid study on black PS foam tray photothermal depolymerization.

Entry	**Contaminant**	M_n (kDa)	*Đ*	styrene (%)	dimer (%)	trimer (%)	toluene (%)	AMS (%)[a]	leftover PS (%)	mass recov. (%)
1	5 wt % *L*-Ascrobic acid	38.4	3.45	49.5	5.0	16.7	1.0	1.4	14.7	86.9
2	100 wt % H_2O	50.5	3.52	45.0	7.2	16.7	0.9	1.4	24.6	94.5
3[b]	5 wt % *L*-Ascrobic acid (aq.)	49.9	3.54	34.2	5.0	12.5	0.4	1.4	29.7	92.7

All % with regard to styrene repeating units.
[a]Alpha-methylstyrene
[b]50 mg *L*-ascorbic acid aqueous solution taken from 20 mg/mL *L*-ascorbic acid in water stock solution

*The synergistic inhibitive effect of L-ascorbic acid and water indicated aqueous vitamin C (L-ascorbic acid) in orange juice might interact with radicals on the polystyrene backbone, therefore lowering styrene yield. Water helped solubilizing and distributing L-ascorbic acid more evenly in black PS foam.

Procedure for Multigram Scale Post-Consumer Waste PS Photothermal Depolymerization

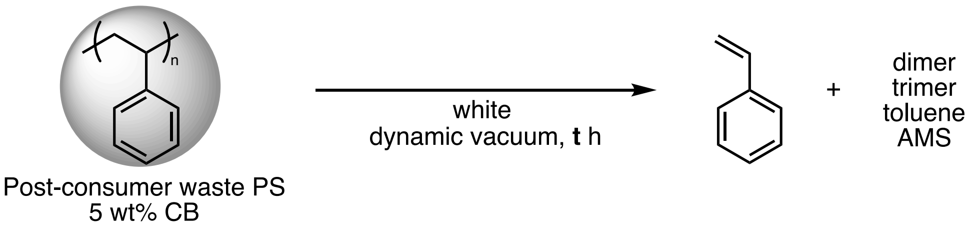

The procedure was slightly modified from the procedure for larger scale photothermal depolymerization under dynamic vacuum. Mixture of clear PS cup, clear PS lid, and white PS foam was hot pressed with carbon black (carbon black = 5 wt %). 3.0 g of the mixture was used in a scintillation vial. A second white LED was placed vertically on the side of the vial to keep the top part of the PS warm. Aluminum foil was used to cover the black adapting piece to protect it from overheating. The 3 g reaction was irradiated for 1h and collected for analysis. The depolymerization results were summarized, and NMR (121.2 mg 1,3,5-trimethoxybenzene added as standard in all portions) and GPC of the depolymerized samples were shown below.

For 6 g-scale reaction, clear polystyrene cup hot pressed with carbon black (6.0 g, carbon black = 5 wt %) was used in a 40 mL-scintillation vial. A 250 mL-round bottom flask was used as receiving flask for the 6 g reaction. A second white LED was placed vertically on the side of the vial to keep the top part of the PS warm. After irradiation for 4 h, the reaction was backfilled with N_2. The receiving flask was weighed (1.6 g product collected) and a 50 mL-round bottom flask was placed as the new receiving flask, and the 6 g reaction setup was pulled to vacuum and back-filled with N_2 for two cycles and left under dynamic vacuum. The reaction was continued for an extra 1 h (0.8 g product collected in receiving flask). The depolymerization results were summarized and GPC of the depolymerized samples were shown below.

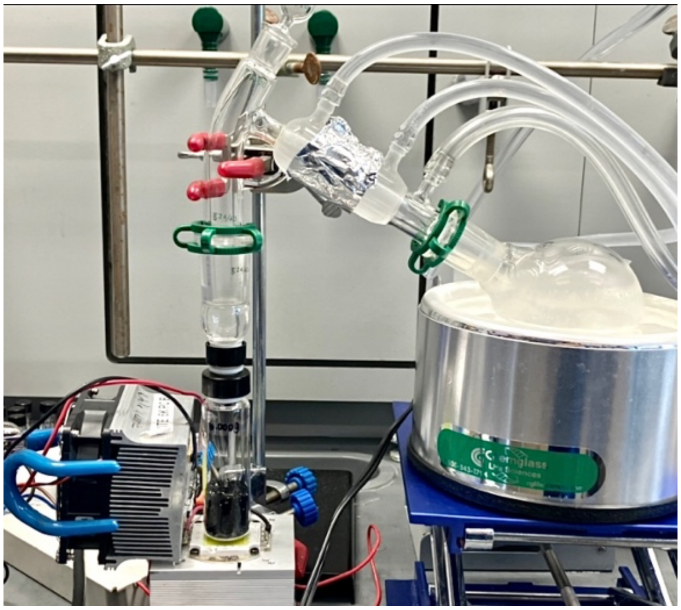

Figure S123. Reaction setup of multigram post-consumer waste PS-CB composite photothermal depolymerization.

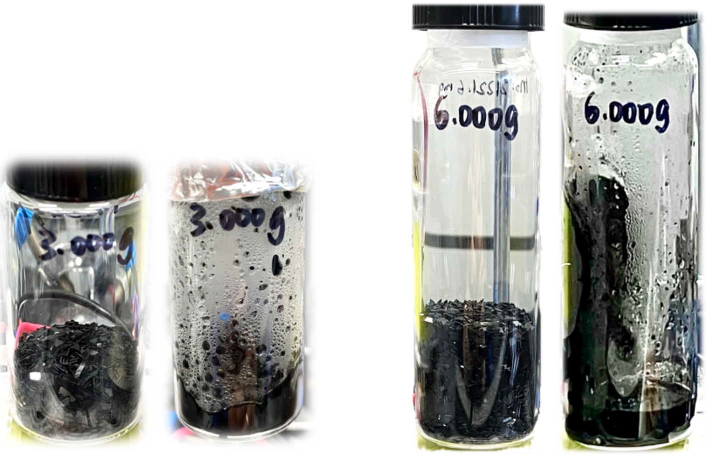

Figure S124. Reaction photos of multigram post-consumer waste PS-CB composite photothermal depolymerization (3 g before/ after, 6 g before/ after).

Table S31. Results of multigram post-consumer waste white PS photothermal depolymerization.

Scale	t	M_n (kDa)	$Đ$	styrene (%)	dimer (%)	trimer (%)	toluene (%)	AMS (%)[d]	leftover PS (%)	mass recov. (%)
3.0 g[a]	1 h	50.5	3.12	38.3	5.3	12.4	0.6	0.5	43.6	>99
6.0 g[b]	5 h[c]	44.4	3.05	43.5	8.4	15.7	0.7	0.3	34.7	>99

All % with regard to styrene repeating units.
[a]Mixture of clear and white PS used
[b]Clear cup used
[c]Increased time length due to penetration depth and small size of light chip
[d]Alpha-methylstyrene (AMS)

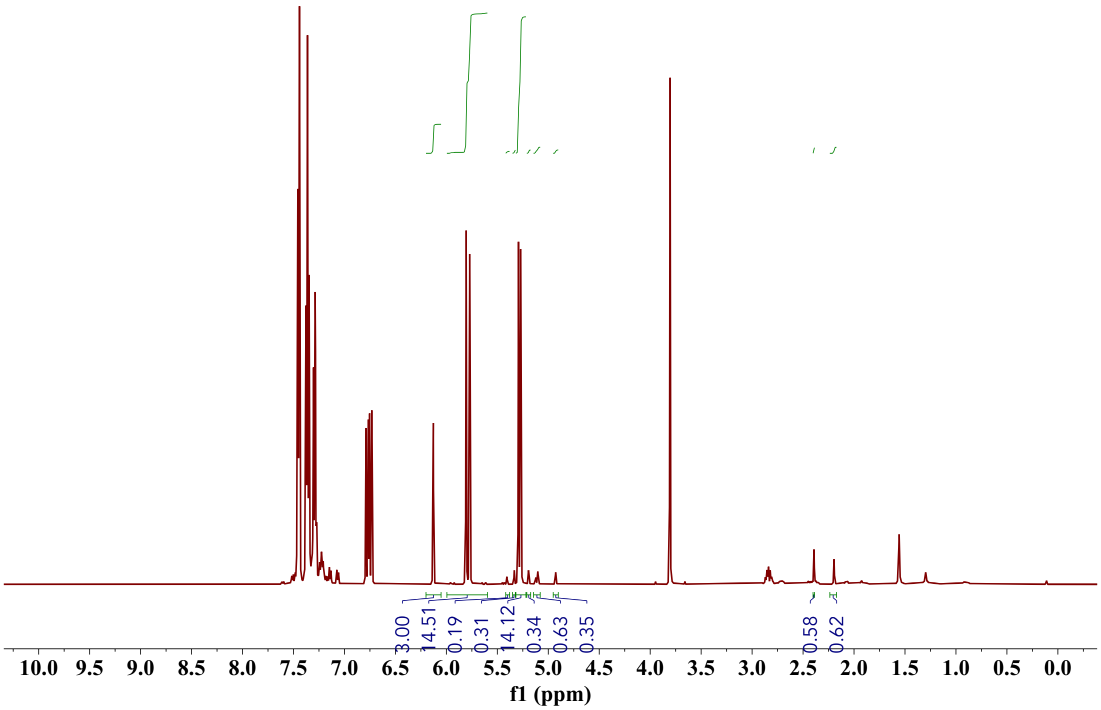

Figure S125. ^{1}H NMR of photothermal depolymerization of 3 g mixed post-consumer waste white PS-CB composite (receiving flask).

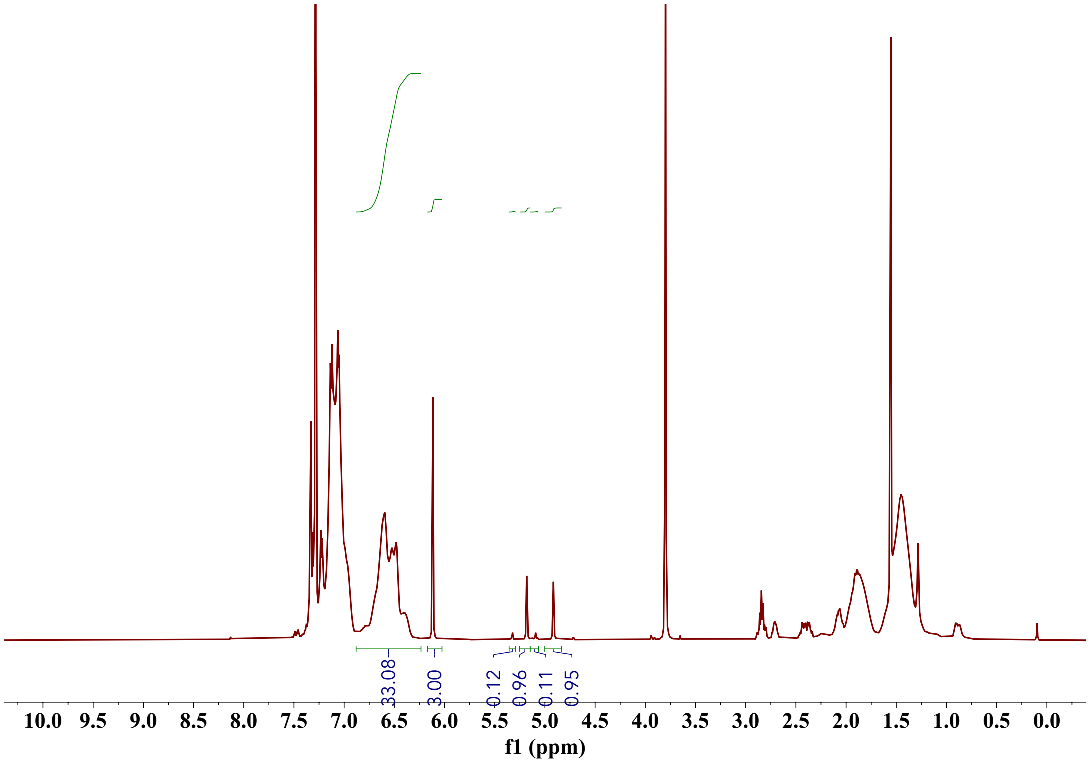

Figure S126. ^{1}H NMR of photothermal depolymerization of 3 g mixed post-consumer waste white PS-CB composite (reaction vial).

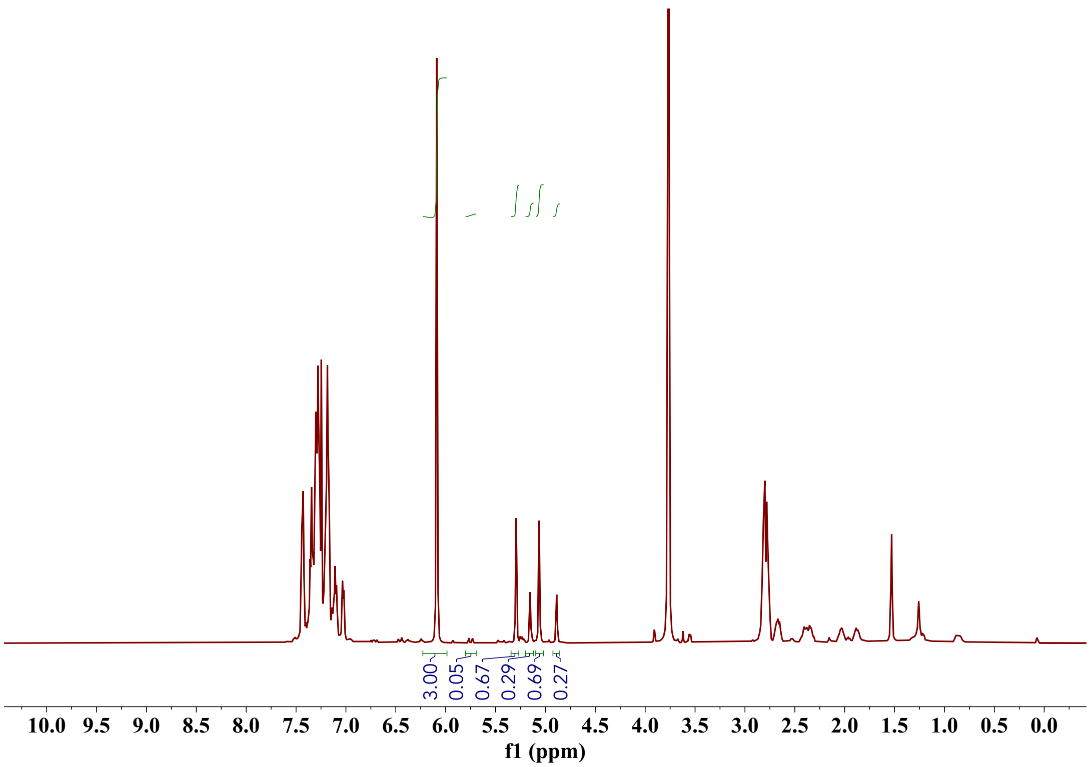

Figure S127. ^{1}H NMR of photothermal depolymerization of 3 g mixed post-consumer waste white PS-CB composite (condenser).

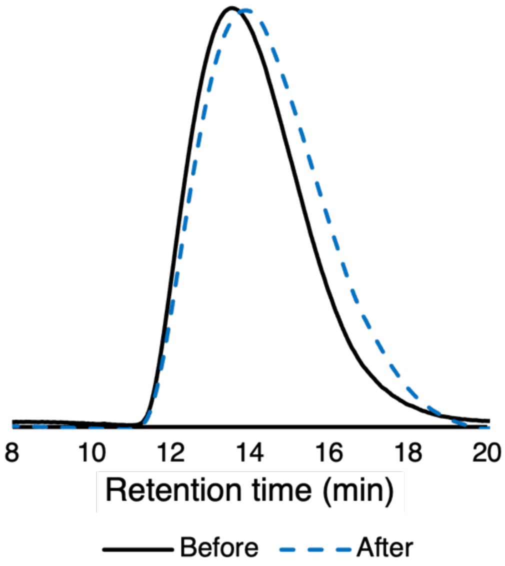

Figure S128. GPC of 3 g mixed post-consumer waste white PS-CB composite after photothermal depolymerization.

Procedure for Photothermal Depolymerization with Focused Sunlight

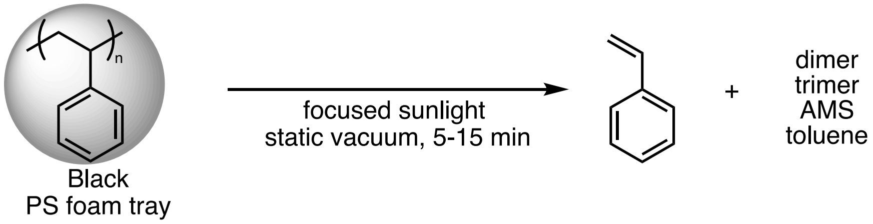

The procedure was slightly modified from the procedure for photothermal depolymerization of post-consumer waste black PS. 50 mg (for Table S24) of black PS foam pieces or pure Sigma Aldrich PS or 100 mg of mixed color PS pieces (for Table S24) were weighed out in a 1-dram vial and sealed under static vacuum. A plastic Fresnel lens (21.1 cm x 29.8 cm) were angled where the focal point is aimed at the bottom of the vial (focal area 1.77 cm^2). The intensity of focused sunlight is estimated by:

21.1 cm x 29.8 cm (Fresnel lens area) / 1.77 cm^2 x unfocused sun intensity (New Jersey, USA), where the unfocused sun intensity data is obtained from https://www.njweather.org/charts. The depolymerization results were summarized, and ^{1}H NMR and GPC of the depolymerized samples were shown below.

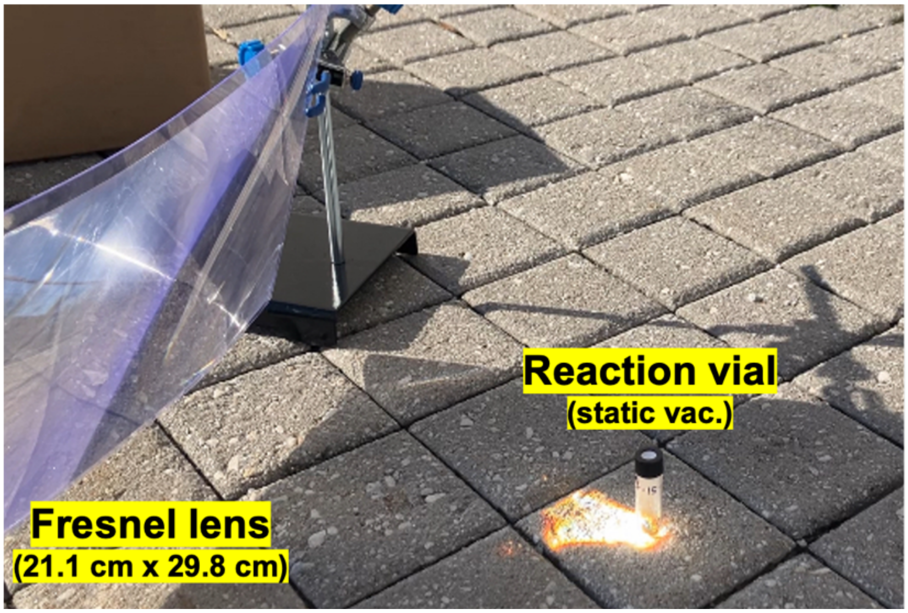

Figure S129. Picture for focused sunlight photothermal depolymerization setup.

Table S32. Results of black PS foam tray after focused sunlight photothermal depolymerization.

Entry	light intensity(W/cm²)[a]	M_n (kDa)	$Đ$	styrene (%)	dimer (%)	trimer (%)	toluene (%)	AMS (%)[b]	leftover PS (%)	mass recov. (%)
1[d]	26.2	-[c]	-	80.2	6.2	9.9	2.4	0.6	0	99.3
2	24.8	-[c]	-	78.3	6.2	12.6	1.9	0.3	0	99.1
3[d]	23.7	-[c]	-	68.9	6.6	11.7	1.5	2.2	0	91.0
4[e]	30.9	73.4	2.57	<1	0	0	0	0	>99	>99

All % with regard to styrene repeating units.
[a]Calculated with reported sunlight intensity x area of lens / 1-dram vial bottom area
[b]Alpha-methylstyrene (AMS)
[c]No polymer peak detected in GPC
[d]Run for 15 min
[e]Commercial PS (M_w = 192 kDa, $Đ$ = 2.41) used

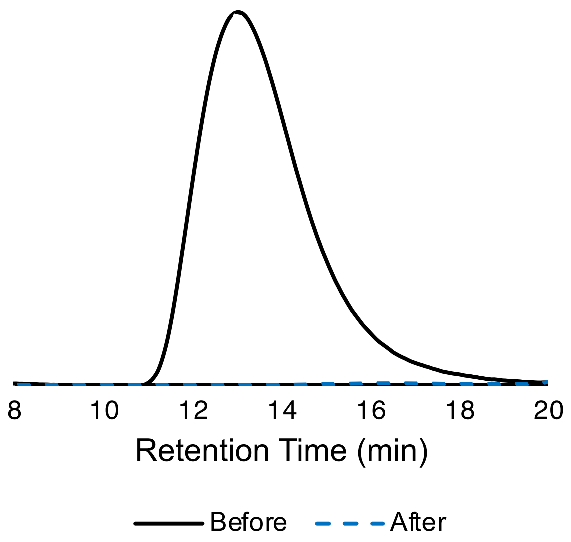

Figure S130. GPC of black PS foam tray after focused sunlight photothermal depolymerization after 5 minutes (Table S32, entry 1).

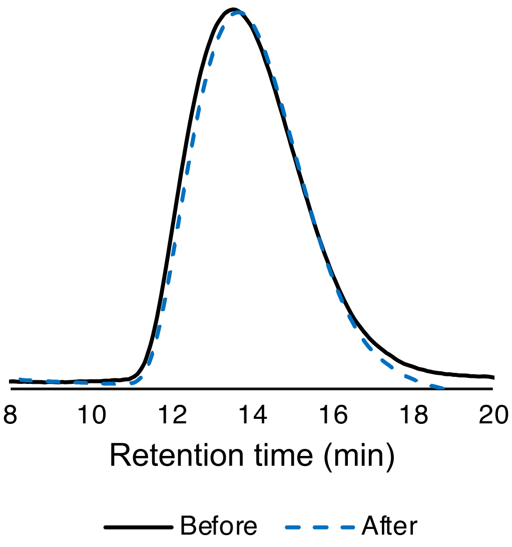

Figure S131. GPC of commercial pure PS from Aldrich (M_w = 192 kDa) after focused sunlight photothermal depolymerization (Table S32, entry 4).

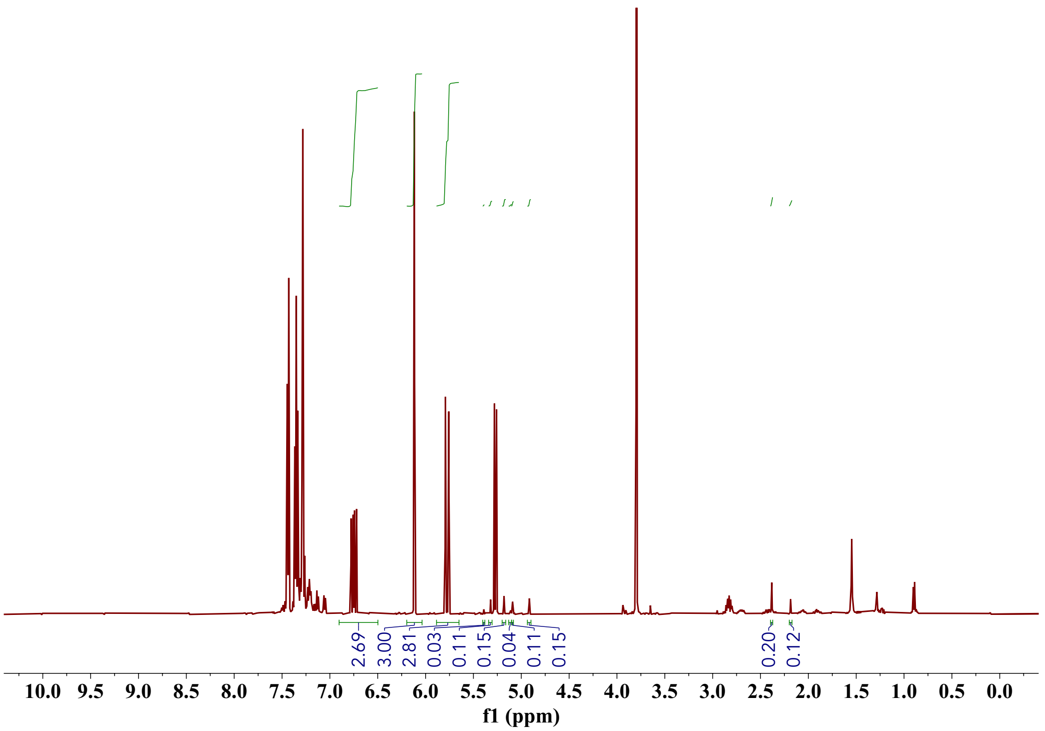

Figure S132. ^{1}H NMR of photothermal depolymerization of black PS foam tray after focused sunlight irradiation (Table S32, entry 1).

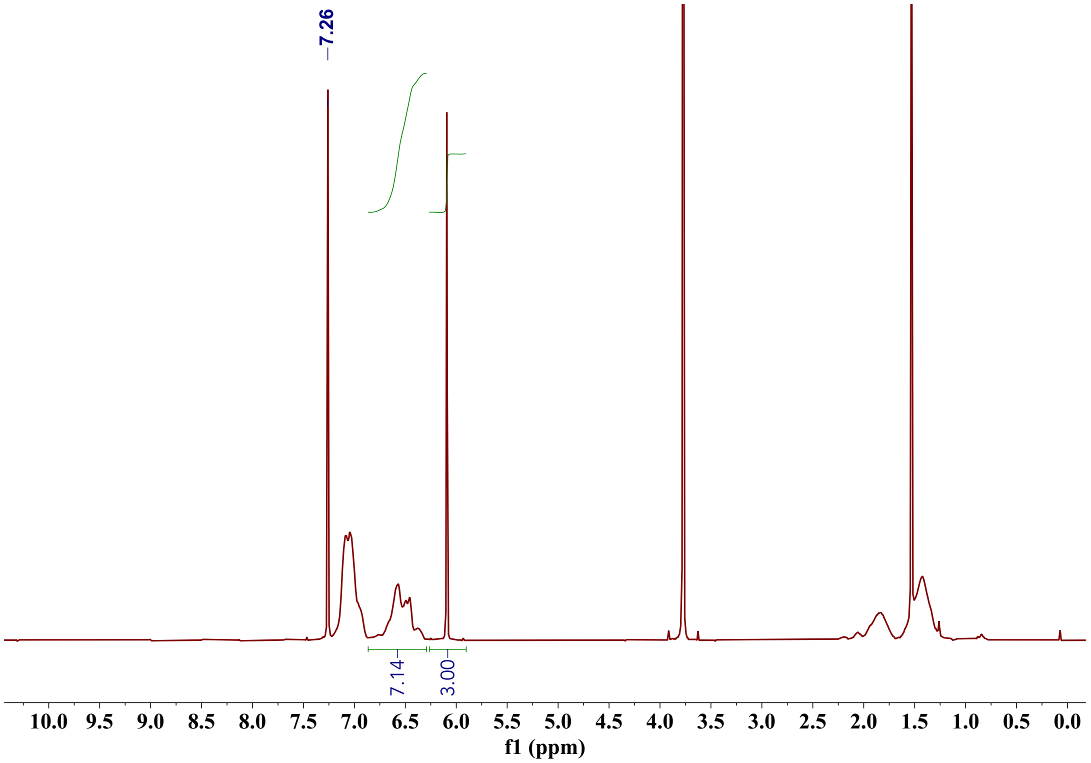

Figure S133. ^{1}H NMR of photothermal depolymerization of commercial pure polystyrene (M_w = 192 kDa) under focused sunlight irradiation (Table S32, entry 4).

Table S33. Results of mixed color post-consumer waste polystyrene after focused sunlight photothermal depolymerization.

Entry[a]	**Black PS (wt %)**	PS Purity (%)[b]	Sample Mass (mg)[c]	M_n (kDa)	$Đ$	styrene (%)	dimer (%)	trimer (%)	toluene (%)	AMS (%)[d]	leftover PS (%)	mass recov. (%)
1	0%	93.8	0, 98.1	65.6	2.60	0	0	0	0	0	> 99	>99
2	10%	93.4	10.2, 87.7	15.0	1.83	71.6	9.6	13.6	1.1	0.2	0.8	96.7
3	25%	92.8	25.1, 74.3	20.5	3.23	66.1	8.7	14.2	1.1	0.2	9.1	>99
4	25%	92.8	25.1, 76.5	17.6	2.10	70.5	8.5	12.7	1.2	0.2	1.4	94.5
5	25%	92.8	25.7, 76.4	17.0	2.19	66.2	8.5	12.7	1.1	0.2	5.3	93.8
6	50%	91.9	50.1, 50.7	18.5	1.96	70.8	6.5	10.5	1.9	0.6	0.8	91.2
7	75%	91.0	75.0, 26.1	-	-	71.0	8.2	12.2	1.8	0.5	0	94.8
8[e]	25%	91.1	100.2	33.1	3.31	66.8	8.5	9.3	1.6	0.6	3.2	>99

All % with regard to styrene repeating units.
[a]Focused sunlight intensity: 31.9 W/cm^2
[b]Calculated with Black PS wt% * 90.1% + (1-Black PS wt%) * 93.8%
[c]Mass of plastic samples listed as "Black PS foam (mg), clear cup (mg)"
[d]Alpha-methylstyrene (AMS)
[e]Multicolor PS mixture, with black PS foam, red cup, clear cup, and yellow PS foam 25 wt% each.

Procedure for Black and Clear Mixed Post-Consumer Waste PS Photothermal Depolymerization

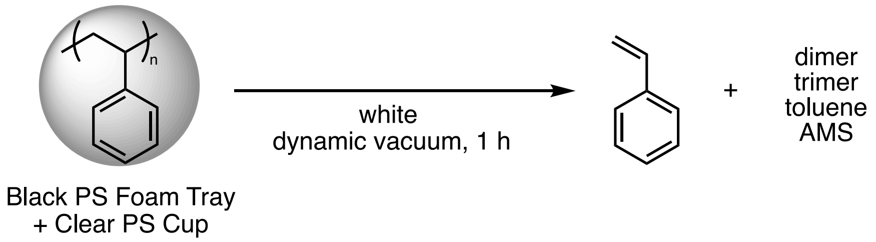

The procedure was slightly modified from the procedure for photothermal depolymerization of post-consumer waste black PS. Clear polystyrene cup was hot pressed directly (1.0 g, diameter = 7 cm) and cut to 1x1 cm pieces. 100 mg mixture of black PS foam and clear PS cup samples were used (black PS foam 10-75 wt %). The reaction setup was pulled to vacuum and back-filled with N_2 for two cycles and left under dynamic vacuum, and depolymerization time was an hour. The depolymerization results were summarized, and GPC of the depolymerized samples were shown below.

Table S34. Results of black and clear mixed post-consumer waste PS photothermal depolymerization.

Entry	**Black PS (wt %)**	PS Purity (%)[a]	Sample Mass (mg)[b]	M_n (kDa)	*Đ*	styrene (%)	dimer (%)	trimer (%)	toluene (%)	AMS (%)[c]	leftover PS (%)	mass recov. (%)
1	10%	93.4	10.0, 90.0	23.8	2.01	40.6	6.2	17.7	0.6	0.1	34.6	>99
			10.2, 90.1	25.4	2.15	40.4	6.7	14.8	0.6	0.2	33.0	95.7
			10.2, 89.9	25.0	2.00	42.6	6.4	17.7	0.6	0.2	29.1	96.6
2	25%	92.8	25.0, 75.0	24.3	2.00	42.1	7.3	16.2	0.6	0.1	31.7	98
			24.8, 74.8	22.4	2.04	43.1	7.6	17.5	0.6	0.1	28.5	97.3
			24.8, 74.8	23.1	1.86	44.1	5.1	15.4	0.6	0.2	26.6	95.7
3	50%	91.9	50.0, 50.0	24.5	2.15	43.9	6.3	14.7	0.8	0.4	28.2	94.2
			49.8, 50.1	24.4	2.19	47.6	7.4	16.4	0.8	0.4	19.6	92.1
			50.3, 49.8	28.5	2.76	43.8	6.3	14.7	0.8	0.4	27.9	93.9
4	75%	91.0	75.2, 24.6	28.4	2.81	46.0	5.8	14.9	0.7	0.2	28.9	99.0
			75.0, 25.0	31.4	2.76	49.1	6.6	18.2	1.0	0.7	12.5	88.0
			74.9, 24.7	32.4	3.44	47.2	6.9	16.6	0.9	0.7	19.1	91.4

All % with regard to styrene repeating units.
[a]Calculated with Black PS wt% * 90.1% + (1-Black PS wt%) * 93.8%
[b]Mass of plastic samples listed as "Black PS foam (mg), clear cup (mg)"
[c]Alpha-methylstyrene (AMS)

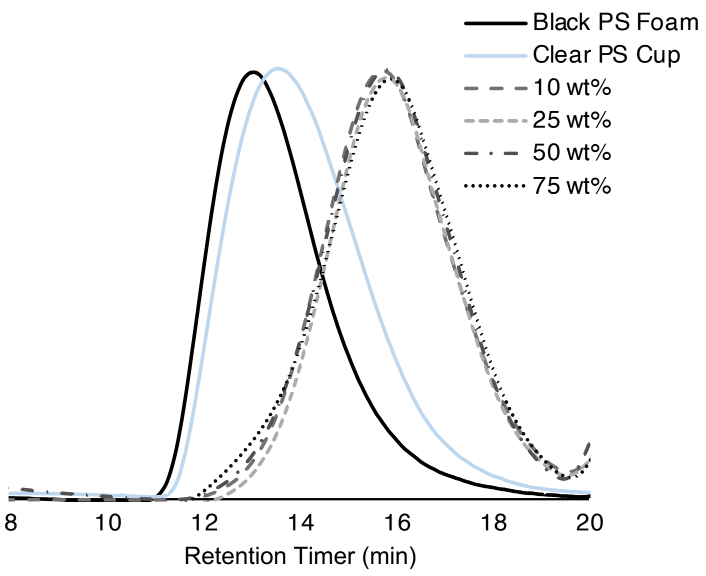

Figure S134. GPC of black and clear mixed post-consumer waste PS before and after photothermal depolymerization.

Procedure for Multicolor Post-Consumer Waste PS Photothermal Depolymerization

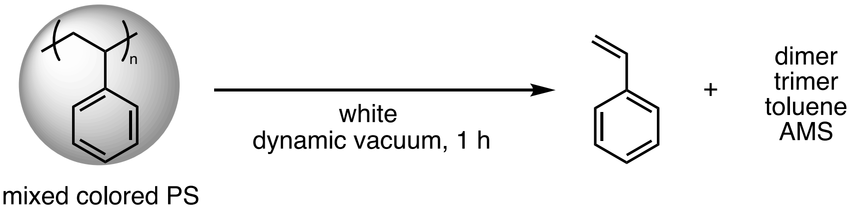

The procedure was slightly modified from the procedure for photothermal depolymerization of post-consumer waste black PS. Clear polystyrene cup was hot pressed directly (1.0 g, diameter = 7 cm) and cut to 1x1 cm pieces. 100 mg mixed colored PS samples were used (red cup, yellow PS foam, clear PS cup, and black PS foam) with 25 wt % each. The reaction setup was pulled to vacuum and back-filled with N_2 for two cycles and left under dynamic vacuum, and depolymerization time was an hour. The depolymerization results were summarized, and GPC of the depolymerized samples were shown below.

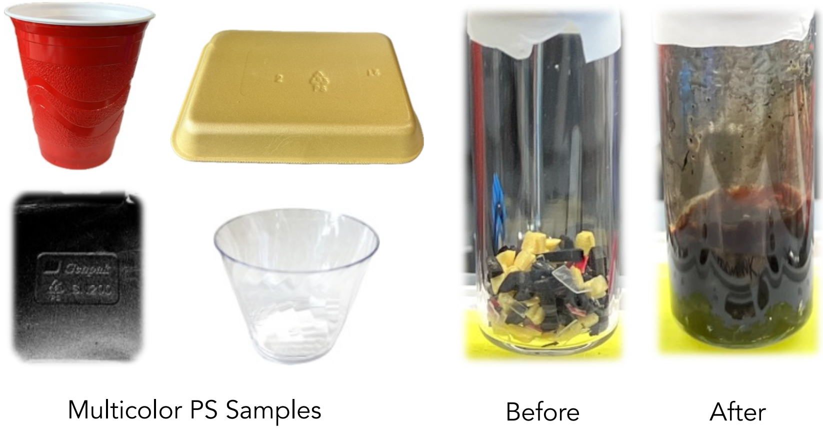

Figure S135. Reaction photos of multicolor post-consumer waste PS samples before and after photothermal depolymerization.

Table S35. Results of multicolor post-consumer waste PS photothermal depolymerization.

PS Purity (%)	Sample Mass (mg)	M_n (kDa)	$Đ$	styrene (%)	dimer (%)	trimer (%)	toluene (%)	AMS (%)[a]	leftover PS (%)	mass recov. (%)
	102.7	23.8	2.01	45.6	6.7	16.9	1.0	0.3	22.4	92.9
91.1	99.8	25.4	2.15	44.7	6.9	16.5	1.1	0.3	26.2	95.6
	99.9	25.0	2.00	43.0	6.9	16.5	1.1	0.3	28.1	95.9

All % with regard to styrene repeating units.
[a]Alpha-methylstyrene (AMS)

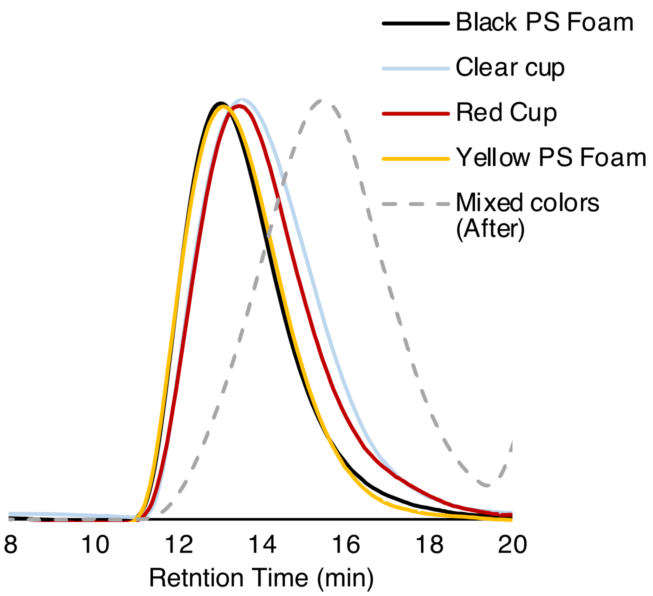

Figure S136. GPC of multicolor PS samples before and after photothermal depolymerization.

Procedure for Thermal Study for Commercial PS

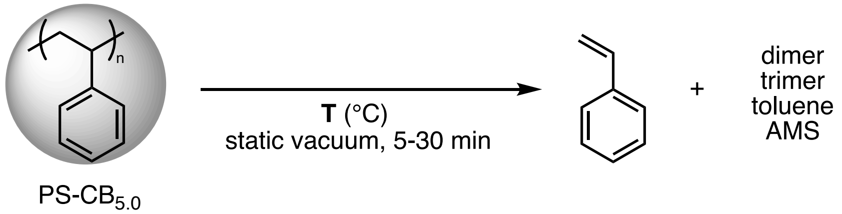

A sand bath (maximum power 1000 W) was preheated at 700 W for 1 hour and alternated between 300 W (5 min) and 700 W (5 min) to maintain the desired temperature (360 - 500 °C). 50.0 mg PS-$CB_{5.0}$ was weighed out in a 1-dram vial with PTFE septa cap. The vial was subjected to three vacuum-N_2 refill cycles and was left under static vacuum after a fourth vacuum pull. The vial was inserted in the sand bath for 5-30 min, with the reaction temperature monitored by a thermocouple inserted at the same depth of the vial. After the reaction, 8.08 mg 1,3,5-trimethoxybenzene stock solution in $CDCl_3$ was added to the reaction vial. Aliquots were taken for NMR analysis and the results of small molecules were summarized in the scatter plots below.

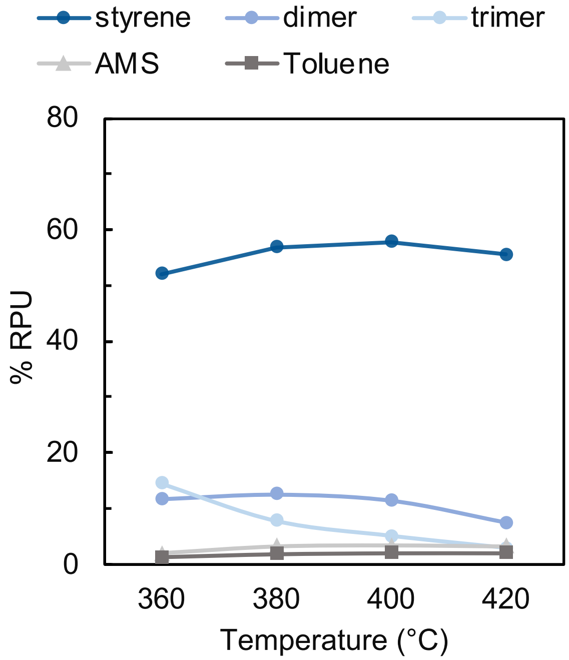

Figure S137. Scatter plot for the results of thermal depolymerization (360-420°C) under static vacuum after 30 minutes.

*Average of 2 trials. Alpha-methylstyrene (AMS) and toluene formed in similar concentrations across all temperatures, and the amount of trimer decreased under higher temperatures. This indicated likely formation of AMS and toluene from decomposition of the trimer (see Table S36 and Figure S140 for more experimental support).[12]

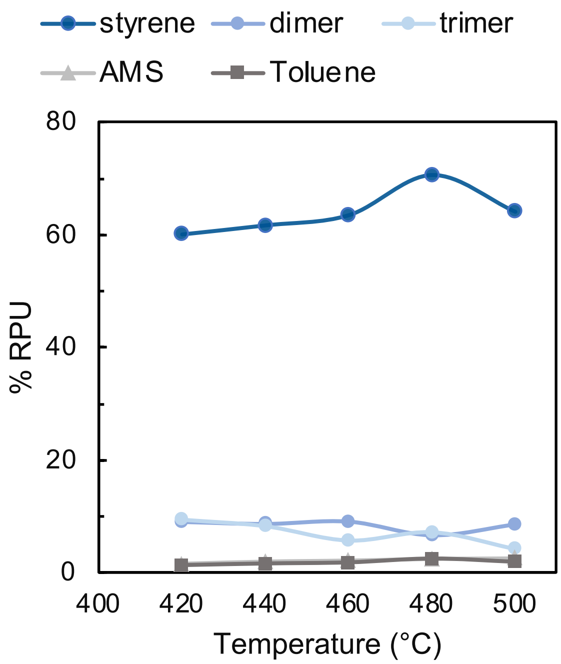

Figure S138. Scatter plot for the results of thermal depolymerization (420-500 °C) under static vacuum after 5 minutes.

*Average of 2 trials. The higher styrene yield here compared to Fig. S140 resulted from 1) higher reaction temperatures and 2) shorter reaction time (less evaporation from the static vacuum system).

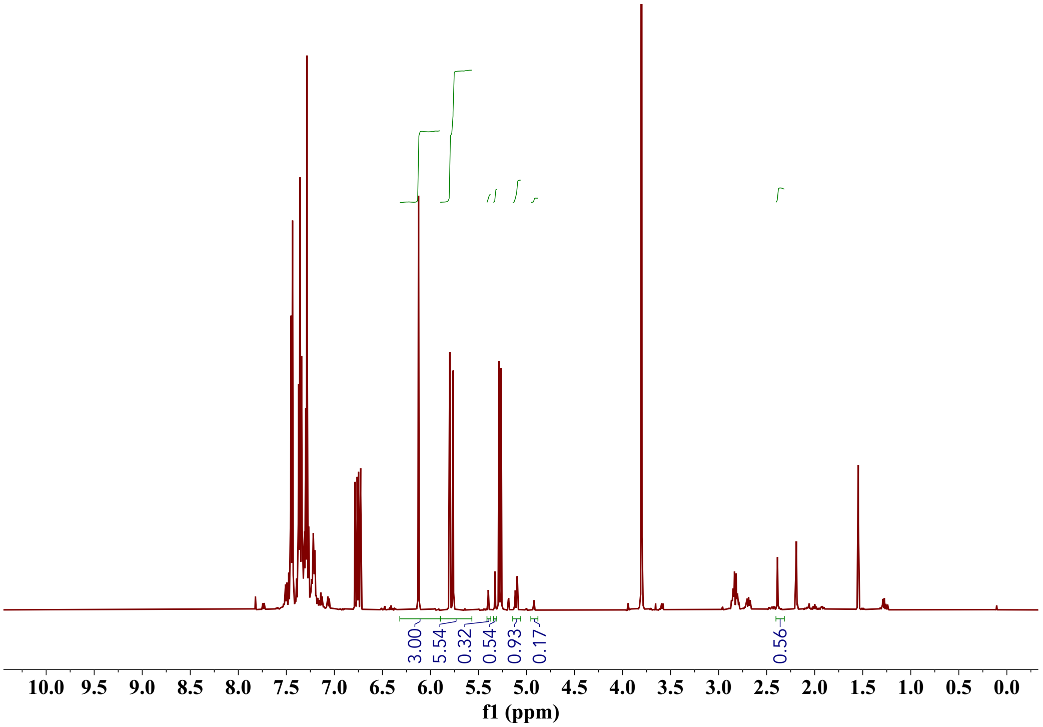

Figure S139. ^{1}H NMR of depolymerization of PS-CB$_{5.0}$ under 420 °C after 30 min.

Procedure for Photothermal Decomposition of Dimer and Trimer Mixture

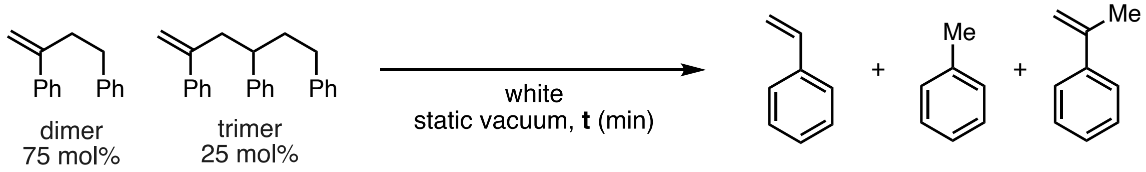

The procedure was slightly modified from the procedure for photothermal depolymerization of post-consumer waste black PS. To a 1-dram vial charged with 2 mg carbon black, 14.0 mg styrene dimer and trimer (3:1 mole ratio) mixture obtained from 6 g photothermal depolymerization were added. The vial was capped with PTFE septa cap, with the bottom frozen in liquid nitrogen and subjected to three vacuum-N_2 refill cycles. The mixture was left under a fourth pull and irradiated under intense white light under static vacuum. After the reaction, 20.2 mg 1,3,5-trimethoxybenzene stock solution in $CDCl_3$ was added to the reaction vial. Aliquots were taken for NMR analysis and the results were summarized below.

Table S36. Results of photothermal decomposition of dimer and trimer mixture.

Entry	**t** (min)	dimer (%)	trimer (%)	styrene (%)	toluene (%)	AMS (%)[a]	dimer/trimer ratio	mass recov. (%)[b]
1	0	75.0	25.0	0	0	0	3.0	> 99
2	30	74.2	21.5	2.5	-[c]	0.7	3.5	96.0
3	60	62.6	3.6	26.3	1.0	3.9	17.5	71.0[d]

[a]Alpha-methylstyrene (AMS)
[b]Mass recovery = (dimer% x 2 + trimer% x 3 + styrene% + toluene% + AMS%) / (0.75 x 2 + 0.25 x 3)
[c]Not quantified due to overlapping peaks in NMR
[d]Lost mass due to unquantified overlapping small molecules in NMR and molecules escaping reaction vial

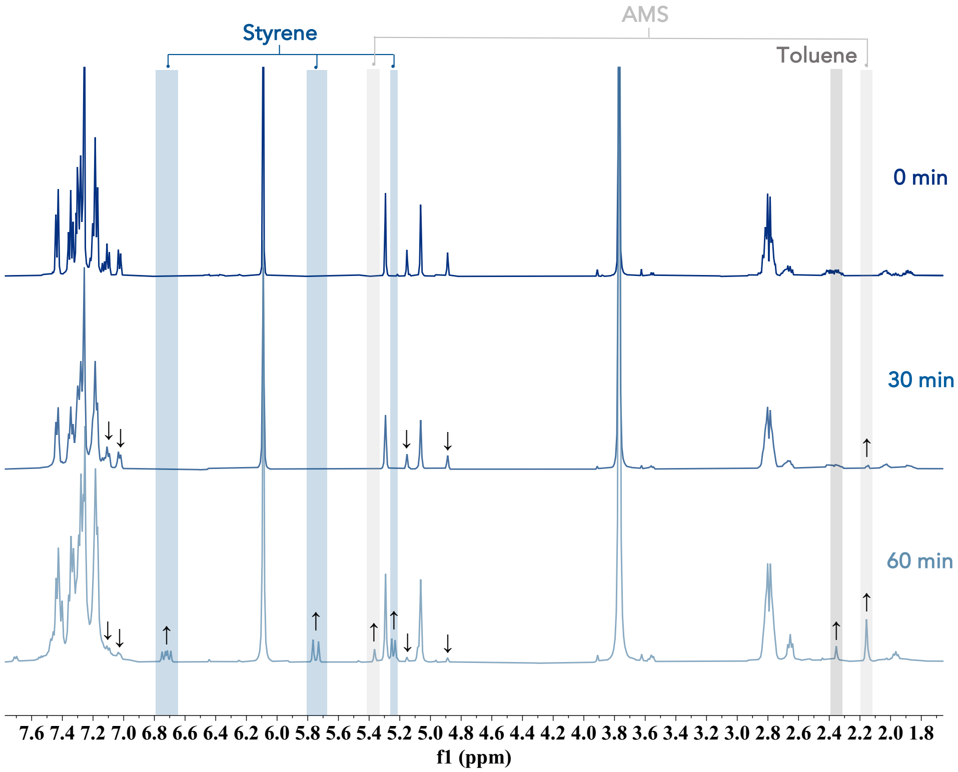

Figure S140. ^{1}H NMR of photothermal decomposition of dimer and trimer mixture.
*The arrows indicated substantial decrease (↓) of trimers and increase (↑) of other small molecule products over time. Products highlighted: styrene (blue), AMS (light grey), toluene (dark grey). This indicated styrene, AMS, and toluene could be formed from trimer cleavage. Also, the decrease of dimer after 60 min of reaction could indicate the formation of styrene from dimer cleavages. However, this result does not rule out other pathways that lead to production of those small molecules.

Procedure for Different Molecular Weight PS Photothermal Depolymerization

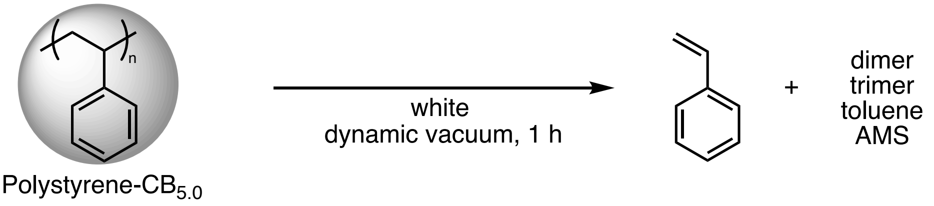

The procedure was slightly modified from the procedure for larger scale photothermal depolymerization under dynamic vacuum. 47.5 mg of polystyrene was mixed with 2.5 mg carbon black in a 1-dram vial. The mixture was irradiated for 1 h under dynamic vacuum and collected for analysis. The depolymerization results were summarized below.

Table S37. Results of photothermal decomposition of dimer and trimer mixture.

Entry	Original M_n (kDa)	Original $Đ$	PS Mass (mg)	leftover M_n (kDa)	leftover $Đ$	styrene (%)	dimer (%)	trimer (%)	toluene (%)	AMS (%)[a]	leftover PS (%)	mass recov. (%)
1	0.54	1.07	46.6	0.54	1.07	0	0	0	0	0	92.3	92.3
2	2.7	1.16	47.5	2.3	1.48	36.6	4.7	15.8	1.0	1.1	33.2	92.4
3	18.0	1.26	47.5	13.7	1.39	38.7	4.3	12.6	0.5	0.5	34.8	91.3
4	38.7	1.98	47.5	24.3	1.80	44.9	8.6	17.8	0.8	0.5	20.3	92.9
5	83.7	2.41	47.5	26.4	2.32	49.8	7.9	15.8	0.4	0.8	20.6	95.3
6	195.9	2.86	47.5	41.7	5.33	50.1	6.3	16.6	0.5	0.3	16.3	90.2

All % with regard to styrene repeating units.
[a]Alpha-methylstyrene (AMS)

Photon Flux Calculation using Chemical Actinometry

The photon flux of the LED light was measured using ferrioxalate solution actinometry.[13] The actinometry solution was prepared in a dark room where 4.416 g $K_3Fe(C_2O_4)_3$ was dissolved in 56.0 mL deionized water and 6.0 mL 0.5 M H_2SO_4. 100 uL Actinometry solution in a 1-dram vial was placed on a 14.0 W 6K white LED light and irradiated. Spectrometric solution was prepared with 500 μL phenanthroline solution (1mg/mL), 5 μL of NaOAc buffer solution (1.23g in 15 mL deionized water), 445 uL deionized water, and 5 μL of the actinometry solution after irradiation. The spectrometric solution was then diluted 400 times to a 3.0 mL total volume for UV-Vis analysis. The reaction rate of Fe(III) to Fe(II) was calculated by the slope of iron-phenanthroline ($Fe(phen)_3$) concentration vs. time, where $Fe(phen)_3$ concentration was determined via $Fe(phen)_3$ standard UV-Vis plot using absorbance at 510 nm. The number of photons absorbed by carbon black was estimated using similar procedure as above by irradiating the actinometry solution (100 uL) with 2.5 mg carbon black. The reacted actinometry solution was processed as described from above and taken for UV-Vis analysis.

The photon flux was calculated with the equation below,

$$\text{Photon Flux (Einstein/s)} = \frac{[\text{Reaction Slope (mmol/s) / 1000}] \times [\text{Reaction Volume } (mL) \text{ / 1000}]}{\text{Quantum yield (mol/Einstein)}}$$

where reaction slope = $\frac{d[Conc.\text{of Fe(phen)3 } before\ dilution]}{d\,t}$ x 4000, Quantum yield = 1.0 [15]

Using slope from Figure S143, the photon flux for high intensity white light was calculated to be (8.6961 mM/s / 1000) x (3.0 mL/1000)/ 1 = 2.61x 10^{-5} Einstein/s. Similarly, applying the slope from Figure S145, the photon flux for high intensity white light was calculated to be (4.5545 mM/s / 1000) x (3.0 mL/1000)/ 1 = 1.37 x 10^{-5} Einstein/s. Hence, we estimated the amount of photons being absorbed by carbon black per reaction being 2.61x 10^{-5} - 1.37 x 10^{-5} = 1.24 x 10^{-5} Einstein/s.

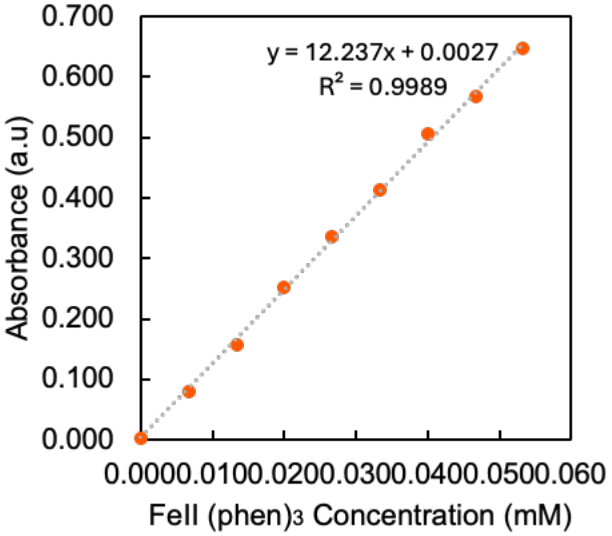

Figure S141. Iron phenanthroline ($Fe(phen)_3$) standard concentration plot.

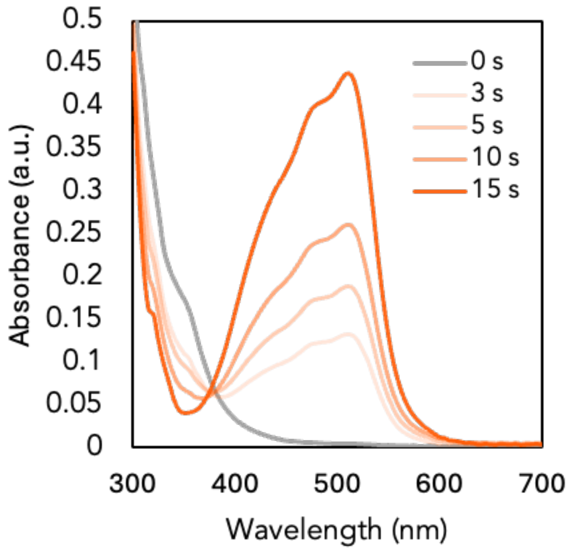

Figure S142. Stacked UV-Vis spectrum of actinometry solution upon irradiation with high-intensity white LED (2.85 W/cm^2, 6000 k) over time.

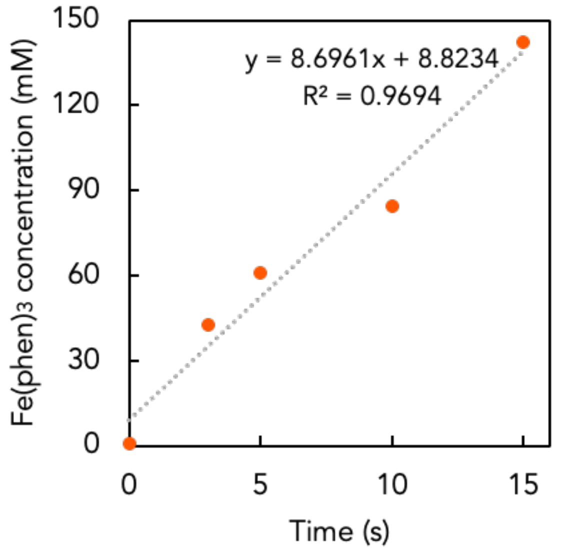

Figure S143. Fe(II) (complexed as Fe(phen)$_3$) concentration in actinometry solution upon irradiation with high-intensity white LED (2.85 W/cm^2, 6000 k) over time.

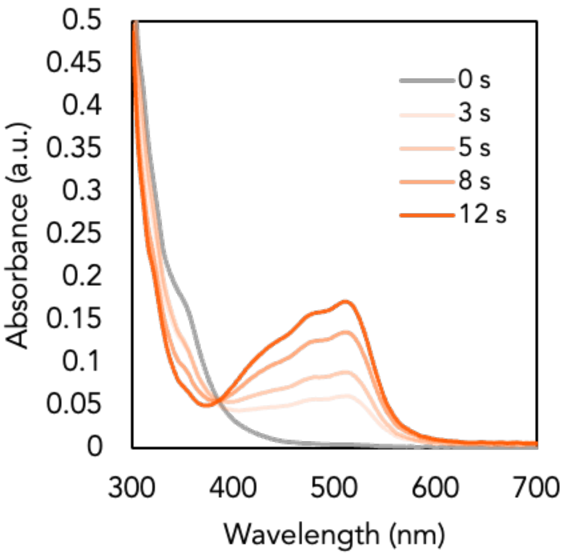

Figure S144. Stacked UV-Vis spectrum of actinometry solution with 2.5 mg carbon black upon high-intensity white LED (2.85 W/cm^2, 6000 k) irradiation over time.

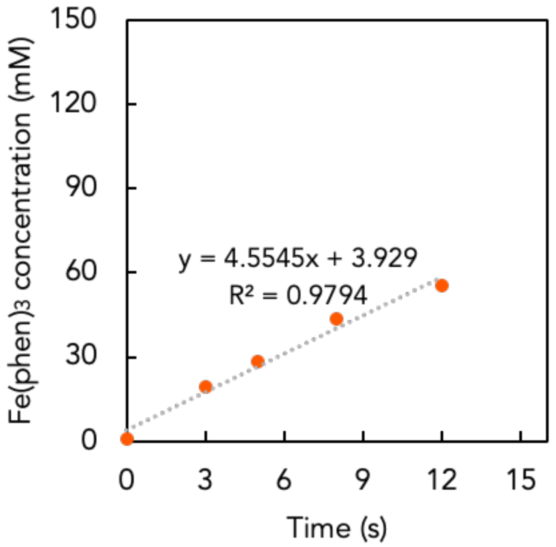

Figure S145. Fe(II) (complexed as $Fe(phen)_3$) concentration in actinometry solution with 2.5 mg carbon black upon irradiation with high-intensity white LED (2.85 W/cm^2, 6000 k) over time.

Movies

Movie S1 is a 30-times-speed recording of the first five minutes of the sunlight depolymerization of black PS foam (see Table S32, entry 3). Movie S2 is a 120-times-speed recording of the 30-minute photothermal depolymerization of commercial PS-$CB_{5.0}$ (see Table S27, entry 4).

References

1. Oh, S.; Stache, E. E. Mechanistic Insights Enable Divergent Product Selectivity in Catalyst-Controlled Photooxidative Degradation of Polystyrene. *ACS Catal.* **2023**, *13*, 10968–10975.

2. Zhao, C.; Sugimoto, R.; Naruoka, Y. A Simple Method for Synthesizing Ultra-high-molecular-weight Polystyrene through Emulsion Polymerization Using Alkyl-9-BBN as an Initiator. *Chin. J. Polym. Sci.* **2018**, *36*, 592–597.

3. Malz, H.; Komber, H.; Voigt, D.; Pionteck, J. Reactions for selective elimination of TEMPO end groups in polystyrene. *Macromol. Chem. Phys.* **1998**, *199*, 583–588.

4. Dement'ev, K. I.; Palankoev, T. A.; Alekseeva, O. A.; Babkin, I. A.; Maksimov, A. L. Thermal depolymerization of polystyrene in highly aromatic hydrocarbon medium. *J. Anal. Appl. Pyrolysis* **2019**, *142*, 104612.

5. Fan, S.; Zhang, Y.; Cui, L.; Xiong, Q.; Maqsood, T. Conversion of Polystyrene Plastic into Aviation Fuel through Microwave-Assisted Pyrolysis as Affected by Iron-Based Microwave Absorbents. *ACS Sustain. Chem. Eng.* **2023**, *11*, 1054–1066.

6. Li, R.; Zhang, Z.; Liang, X.; Shen, J.; Wang, J.; Sun, W.; Wang, D.; Jiang, J.; Li, Y. Polystyrene Waste Thermochemical Hydrogenation to Ethylbenzene by a N-Bridged Co, Ni Dual-Atom Catalyst. *J. Am. Chem. Soc.* **2023**, *145*, 16218–16227.

7. Ukei, H.; Hirose, T.; Horikawa, S.; Takai, Y.; Taka, M., Azuma, N., & Ueno, A. Catalytic degradation of polystyrene into styrene and a design of recyclable polystyrene with dispersed catalysts. *Catal. Today* **2000**, *62*, 67–75.

8. Tureau, M. S.; Epps, T. H. Effect of Partial Hydrogenation on the Phase Behavior of Poly(isoprene-*b*-styrene-*b*-methyl methacrylate) Triblock Copolymers. *Macromolecules* **2012**, ***45***, 8347–8355.

9. Lindemann, R. F. Flame-retardants for polystyrenes. *Ind. Eng. Chem.* **1969**, *61*, 70–75.

10. Colton, J. S.; Suh, N. P. The nucleation of microcellular thermoplastic foam with additives: Part II: Experimental results and discussion. *Polym. Eng. Sci*. ***1987***, *27*, 493–499.

11. Lower, E. S. Calcium stearate in resins and resinous polymers: part 2. *Pigm. Resin Technol.* **1996**, *25,* 18–23.

12. Levine, S. E.; Broadbelt, L. J. Reaction pathways to dimer in polystyrene pyrolysis: A mechanistic modeling study. Polym. Degr. Stab. **2008**, *93*, 941-951.

13. Hatchard, C. G.; Parker. A new sensitive chemical actinometer-II. Potassium ferrioxalate as a standard chemical actinometer. *Proc. R. soc. Lond. Ser. A Math. Phys. Sci.* **1956**, *235*, 518-536.
